# Robotic Centrifugal Microfluidic Automation Enables Library Preparation of Nine Samples with a Tenfold Reduction in Reagent Consumption for Decentralized Sequencing

**DOI:** 10.1021/acs.analchem.6c03501

**Published:** 2026-09-21

**Authors:** Trong Nguyen, Viola Dreyer, Yumi Kaku, Peter Juelg, Tobias Hutzenlaub, Stefan Niemann, Nils Paust, Jacob Friedrich Hess

**Affiliations:** † Hahn-Schickard, 79110 Freiburg, Germany; ‡ Laboratory for MEMS Applications, IMTEK − Department of Microsystems Engineering, 9174 University of Freiburg, 79110 Freiburg, Germany; § Research Center Borstel, Leibniz Lung Center, 23845 Borstel, Germany; ∥ German Center for Infection Research (DZIF), 20359 Hamburg, Germany

## Abstract

Next-generation sequencing (NGS) has become a crucial tool for medical diagnostics in decentralized laboratories. While sequencing and bioinformatics themselves employ standardized pipelines, wet laboratory workflows in these settings still rely on manual pipetting and sample handling. In particular, the library preparation procedures represent a critical bottleneck, accounting for up to 50% of total NGS costs and involving complex steps. Automation offers potential solutions to these challenges. However, existing automation approaches have limitations in satisfying throughput, investment costs, costs per sample, and degree of automation. Here, we implement a robotic centrifugal microfluidic platform around the Illumina Nextera XT DNA Library Preparation Kit or RoCM-LP platform, which supports library generation from microbial genomes. The RoCM-LP platform combines a centrifugal microfluidic-based cartridge with a pipetting robot, capable of producing nine libraries per run at low-medium initial investment costs ($50k–70k). The costs per sample were substantially reduced due to a tenfold decrease in reagent consumption in comparison to the manual reference workflow, and the number of manual handling steps was minimized from 45 to just three, namely reagent preparation (placing required reagents into the RoCM-LP platform), sample loading, and library retrieval. The approach achieved quality metrics comparable to those of the manual reference workflow and met all required sequencing quality thresholds across four *Mycobacterium tuberculosis* complex (MTBC) strains including drug-susceptible, BCG vaccine (Pasteur Bacillus Calmette–Guérin), multidrug-resistant (MDR), and extensively drug-resistant (XDR) strains for tuberculosis (TB) samples investigated, with an average coverage depth of 107, an average mapped read percentage of 97%, and an average coverage breadth percentage of 98%.

## Graphical abstract

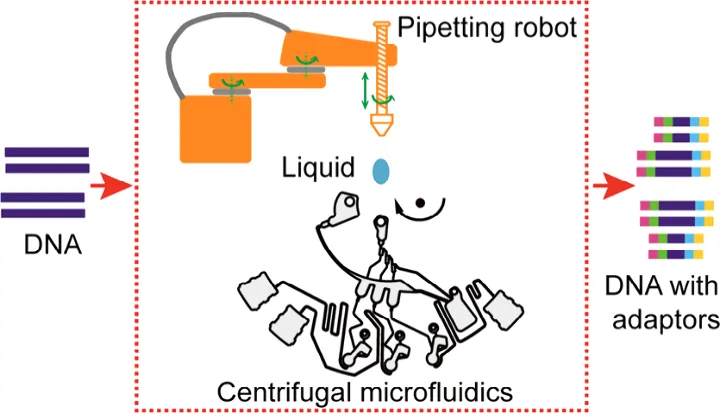

## Introduction

In recent years, next-generation sequencing (NGS) has become more important in healthcare diagnostics.^2^ It helps to identify tumors and provide the complete profiling of different cancer types for personalized therapy. In addition, it also detects novel infectious diseases for rapid diagnosis and outbreak investigation.^3^ NGS can be performed close to the patients in decentralized laboratories or in high-throughput sequencing centers.^4,5^ Typical decentralized laboratories are local NGS research centers in remote areas, or small laboratories in hospitals or ambulatory health care settings with in-house NGS capability. High-throughput sequencing centers offer the advantage of lower costs per sample but require samples to be transferred from patients to the sequencing center and high investment costs for equipment and facilities. Decentralized laboratories shorten the turnaround time from sample to analysis, reduce investment costs, and improve the reliability of generated results due to in-house standardized methods.^2,6,7^ The main barrier to decentralized laboratories is library preparation, which is costly^8^ and complex.^9^ Automation is a suitable tool to overcome these challenges and to enable NGS applications in decentralized laboratories.^10^ The suitability of an automated approach for decentralized laboratories can be evaluated based on four key requirements: sample throughput, investment costs, costs per sample, and degree of automation. First, the sample throughput should range from 5 to 6 samples per run,^2,7,8,11,12^ as decentralized laboratories process fewer samples than high-throughput sequencing centers, which process hundreds to thousands of samples per run.^13–15^ Second, the investment in equipment should be affordable and below $70k.^16–18^ Third, costs per sample must be as low as possible.^19^ Fourth, a high degree of automation is essential because, in these laboratories, there is a lack of expertise among personnel,^20^ and a risk of contamination by manual handling steps.^21^

To automate liquid handling for library preparation workflows, there are many available solutions. A comparison of these solutions is provided in Table 2. One popular solution uses pipetting platforms equipped with motor-driven robot arms, pipette heads, and pipette tips to aspirate and dispense liquid. They often apply the air-displacement working principle for pipetting, using air volume inside pipette tips to draw liquid up. The most common pipetting platforms with a focus on high sample throughput like the Agilent Bravo Platform,^22^ the Beckman Coulter Biomek i7,^23^ and the MGISP-960 ^24^ process 96–384 samples per run and have been applied in bacterial genome sequencing,^25^ transcriptome profiling,^26^ and SARS-CoV-2 characterization.^27^ However, they require substantial investment costs ($100k–200k),^28^ and their disposable consumption drives costs per sample upward when run at low sample numbers. They achieve high automation with three manual steps (reagent preparation, sample loading, library retrieval).

Positive-displacement pipetting uses capillary pistons in direct contact with the liquid, and eliminating the air cushion enables pL-level volumes that lower costs per sample. The Mosquito Gen3 platform from SPTLabTech implements this approach^29^ and processes up to 384 samples per run. However, high investment costs ($90k–140k), expensive disposable tips, and the absence of integrated thermal cycling (>4 manual steps, requiring repeated plate transfers to a thermocycler) limit its use in decentralized laboratories. A separate approach uses integrated fluidic chips with on-chip nL-scale valves: the Fluidigm Juno (MOgene) processes up to 192 samples per run,^30^ making it suitable for single-cell sequencing applications.^31^ While this platform is compact and does not require substantial investment costs ($20k–60k),^32^ its expensive microfluidic chips increase costs per sample ($300–1000 for one cartridge per run).^33^ In terms of degree of automation, the Fluidigm Juno requires only 3 manual handling steps like the high-throughput systems.

Medium-throughput air-displacement systems target the decentralized laboratories more directly. The Tecan MagicPrep NGS processes 8 samples per run.^34^ The system has been applied to clinical microbial whole-genome sequencing^35^ and respiratory viral pathogen detection.^36^ The investment cost is moderate ($50k–70k)^37^ but $400 cartridges^38^ elevate costs per sample, and an off-instrument fragmentation step pushes manual handling above six steps.^34^ The BioQule NGS system from Revvity adds integrated fluorescence readout^39,40^ and processes 8 samples per run with three manual steps, but shares the same trade-off: $400–500 cartridges drive costs per sample.^41^

A purely centrifugal microfluidic implementation reduced reagent consumption by 40% and processed up to two samples in parallel.^42^ Three limitations remained. First, throughput was limited to two samples per cartridge, because the complex microfluidic operations and stick-pack-stored reagents occupied a substantial fraction of the cartridge footprint. Second, users had to pipette reagents into each of six inlet chambers before operation (>6 manual steps per sample), introducing a risk of handling error. Third, reagent miniaturization reached only 40%. The smaller volumes were not feasible, as each reagent required an individual metering structure.

Here we present the Robotic Centrifugal Microfluidics (RoCM-LP) platform, which combined a pipetting robot with centrifugal microfluidic cartridges. RoCM-LP processed up to nine samples per run (in increments of three, owing to the three-sample slice format) at $50k–70k investment cost, achieved a tenfold reagent reduction, and limited manual handling to three steps: reagent preparation, sample loading, and library retrieval. Two design features enabled this performance. A two-stage aliquoting structure provided precise 0.5 μL metering, and a one-pot reaction chamber confined all biological reactions to a single chamber, thereby limiting the sample loss associated with inter-chamber liquid transfer. Because the pipetting robot supplied reagents directly, nine samples can be processed in parallel without on-cartridge reagent storage. The bulk volumes were instead prepared once in the liquid storage area and can serve multiple runs. As a proof of concept, we demonstrated TB diagnostics using DNA from four diverse *Mycobacterium tuberculosis* complex (MTBC) strains.

## Materials and Methods

### Samples

The Research Center Borstel provided DNA from four different MTBC strains as sample inputs: S1, H37Rv reference strain with (4,417,942 bp);^43^ S2, an attenuated BCG vaccination strain with a virulence-reducing deletion (4,374,522 bp);^44^ S3, an MDR strain (4,245,814 bp) carrying 1396 single nucleotide polymorphisms (SNPs) and three large deletions relative to S1;^45^ and S4, an XDR strain (4.2–4.4 Mbp) with multiple insertions and deletions conferring resistance to several TB drugs.^46,47^ S3 and S4 are clinically relevant strains. Inputs were 0.5 ng for S1 and 0.05 ng for S2–S4, in 0.5 μL per sample.

### Reagents

The Illumina Nextera XT DNA library preparation kit served as the biological reference workflow and supplied all reagents for tagmentation, amplification, binding, washing, and elution; volumes are listed in Table 1.

**Table 1:** Reagent and Material Consumption in the Manual Reference Workflow and the Automated Workflow^a^

reagent/material	manual reference workflow	automated workflow
DNA sample	5 μL	0.5 μL
tagmentation buffer	10 μL	1 μL
Amplicon tagmentation mix	5 μL	0.5 μL
neutralized tagmentation buffer	5 μL	0.5 μL
PCR master mix	15 μL	1.5 μL
UDI primers	10 μL	1 μL
binding buffer	90 μL	9 μL
washing buffer	400 μL	36 μL
elution buffer	180 μL	18 μL
water with 1% BSA	0 μL	1 μL
neodymium disc magnets (1 mm × 1 mm)^32^	0 piece	1 piece

a In the automated workflow, 1 μL of primers from Illumina ready-to-use Unique Dual Index stock^48^ was dried and pre-stored in the cartridge. To preserve constant liquid–liquid ratio, 1 μL of water containing 1% v/v bovine serum albumin (BSA) was used. In addition, a single neodymium disc magnet (1 mm × 1 mm)^50^ was used in automation workflow to enhance the bead mixing. The elution buffer was resuspension buffer in Nextera XT Library Preparation Kit^49^.

### Biological Manual Reference Workflow

Illumina Nextera XT DNA Library Preparation protocol consists of tagmentation, tagmentation neutralization, amplification, binding, washing, and elution. Reagent volumes are listed in Table 1. First, the DNA sample is mixed with two reagents: tagmentation buffer (TD) and amplicon tagmentation mix (ATM). The ATM contains enzymes called transposases, which cut the DNA into smaller fragments and attach adaptors to both ends of each piece. The mixture is then heated at 55 °C for 5 min in a thermal cycler to allow this process (called tagmentation) to occur. The neutralized tagmentation buffer (NT) is added and the sample is cooled to 10 °C, which stops the reaction. The PCR Master Mix and Illumina Unique Dual Index primers^48^ are then added; the primers carry indexing sequences complementary to the transposase-introduced adaptors, enabling sample pooling for parallel sequencing. Amplification runs for 12 thermal cycles. Amplified DNA is then bound to hydroxyl-coated AMPure magnetic beads in a polyethylene glycol (PEG) binding buffer, which promotes binding of DNA to the bead surfaces. Beads are washed with 80% ethanol/20% water to remove fragments below 100 bp and dimers. DNA is finally eluted from the beads in elution buffer.^49^ provides the complete manual reference workflow with details for each step.

### Automated Workflow

On the RoCM-LP platform, most reagent volumes were reduced tenfold relative to the manual reference (minimum 0.5 μL; Table 1), with the washing buffer reduced 11fold; amplification was extended from 12 to 14 cycles to recover sufficient DNA for sequencing. To reduce processing steps, primers and magnets were pre-stored in the cartridge. Then 1 μL of primers was deposited in each reaction chamber and dried overnight at 50 °C, with the original primer volume in solution replaced by water containing 1% BSA to preserve the liquid-volume ratio. Neodymium disc magnets (1 mm × 1 mm)^50^ were placed at predefined locations adjacent to the reaction chambers. These internal magnets improve bead mixing inside the reaction chambers and remained in their pre-stored positions during tagmentation and amplification to prevent potential interference with the biological reactions. After amplification, the robot relocated them to the bottoms of the reaction chambers for bead mixing in binding, washing, and elution. The complete automated workflow with rotations, temperatures, and time parameters can be found in .

### Robot and Tools

The basic principle of RoCM-LP and a system characterization was recently presented by our group.^1^ Figure 1A shows the overall RoCM-LP layout used for this work on NGS library preparation. A SCARA robot with four degrees of freedom (including one rotational axis) operates two interchangeable tools. The pipette tool picks up tips, aspirates from the liquid storage, and dispenses into the reagent inlets. The magnet tool houses three neodymium disc magnets (8 mm × 15 mm) that drive the internal magnets in the reaction chambers for bead mixing. The liquid storage holds tubes of 0.5, 1.5–2, 5, and 10 mL on a cooling element that keeps reagents chilled throughout the run. A front-mounted tablet provides the user interface.

**Figure 1:**
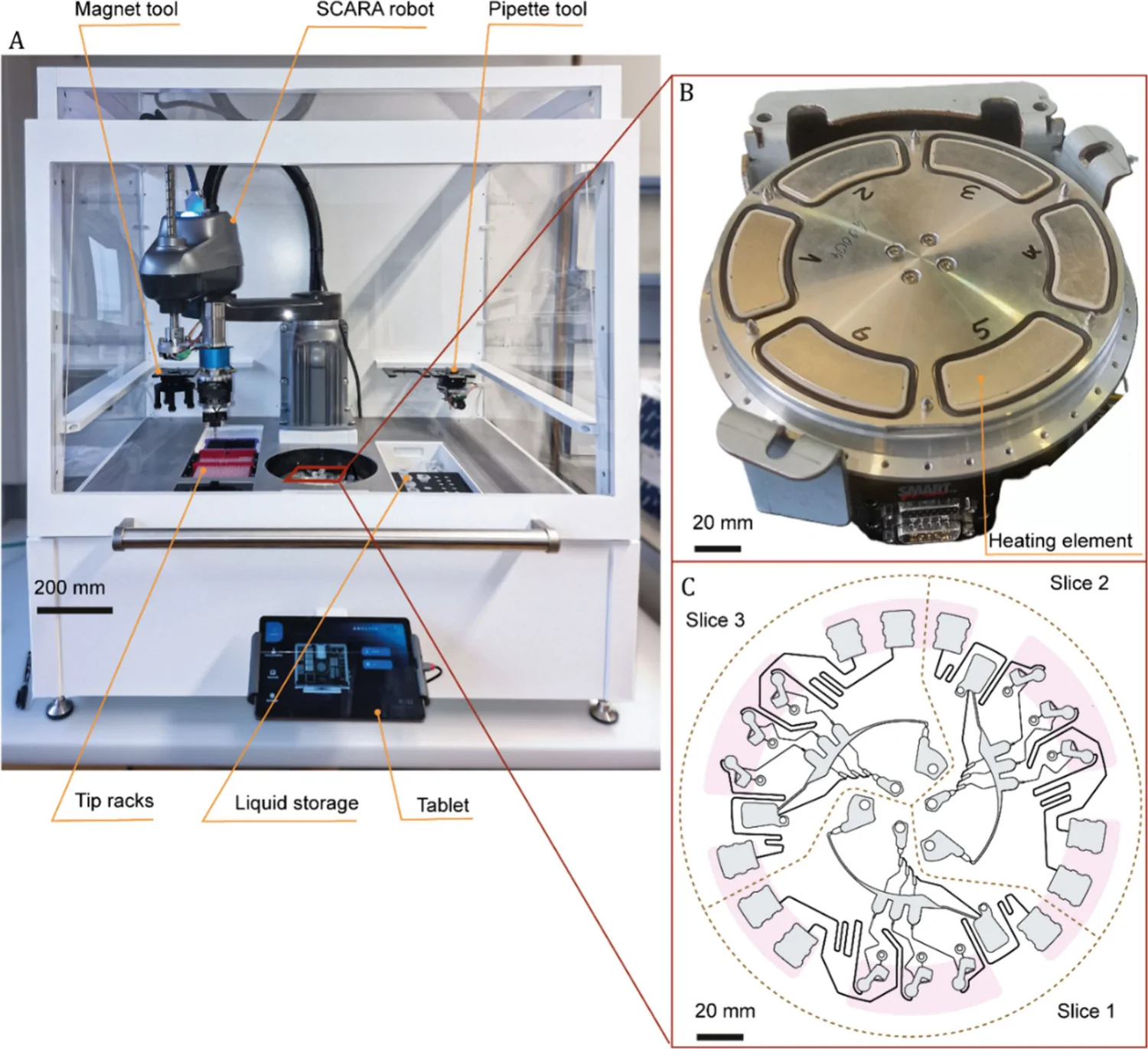
(A) The RoCM-LP platform comprises three main components: a SCARA (selective compliance articulated robot arm) robot with four degrees of freedom (including a rotational axis), a central rotor, and a centrifugal microfluidic cartridge mounted on the rotor. The robot uses a pipette tool (right) to aspirate and dispense liquids, and a magnet tool (left) to drive bead mixing and move the internal magnets inside the reaction chambers; pipette tips and reagents are held in the tip racks and liquid storage. (B) The rotor spins and heats the cartridge via six heating elements. (C) The cartridge contains three identical slices, each preparing three libraries (nine total); heating-element positions relative to the fluidic structures are highlighted in pink. Figure 2 details a single slice.

### Rotor

Figure 1B shows the central rotor, which spins and heats the cartridge mounted on top. Six heating elements reach 120 °C, and the rotor spins up to 60 Hz with a maximum acceleration/deceleration of 25 Hz/s. Vacuum pressure holds the cartridge during rotation. In addition, the rotor also communicates with the robot for optional synchronized liquid dispensing under rotation, or simple dispensing and movement of the internal magnets under standstill. The temperature of six heating elements was also calibrated using infrared thermal camera ()

### Cartridge

Figure 1C shows the complete cartridge, which has three identical slices and prepares nine libraries in parallel; Figure 2 details a single slice. The initial design was developed in CAD (SolidWorks 2024) and MATLAB Simulink simulations^51^ and manufactured at Hahn-Schickard. A KERN Evo mill (KERN Microtechnik, Germany) cut a positive structure into a 6 mm thick, 160 mm diameter polymethylmethacrylate (PMMA) plate; a special-grade PDMS (Elastosil RT-607, Wacker Chemie, Germany) formed the negative. A HEX01 hot embosser (Jenoptik, Germany) replicated the structure into 400 μm thick, 140 mm diameter cyclic olefin polymer (COC) films and thermally sealed the cartridge with a 200 μm COC film.^52^ A thermal insulation cover (Figure S3) ensures temperature homogeneity during rotation and heating, with an air gap acting as the insulating layer. The cover was made by cutting a 5 mm thick, 160 mm diameter PMMA plate on a CO2-laser VLS4.75 (Universal Laser Systems, USA), sealing it with 3M pressure tape, and bonding it to the cartridge with TESA universal double-sided tape. To prevent liquid transport from the aliquoting structures into the pneumatic chambers under centrifugal force while avoiding cross-contamination between closely spaced reaction chambers, polytetrafluoroethylene (PTFE) porous membranes (4 μm average pore size)^53^ were placed directly above the DNA inputs (Figure 2) for air ventilation.

**Figure 2:**
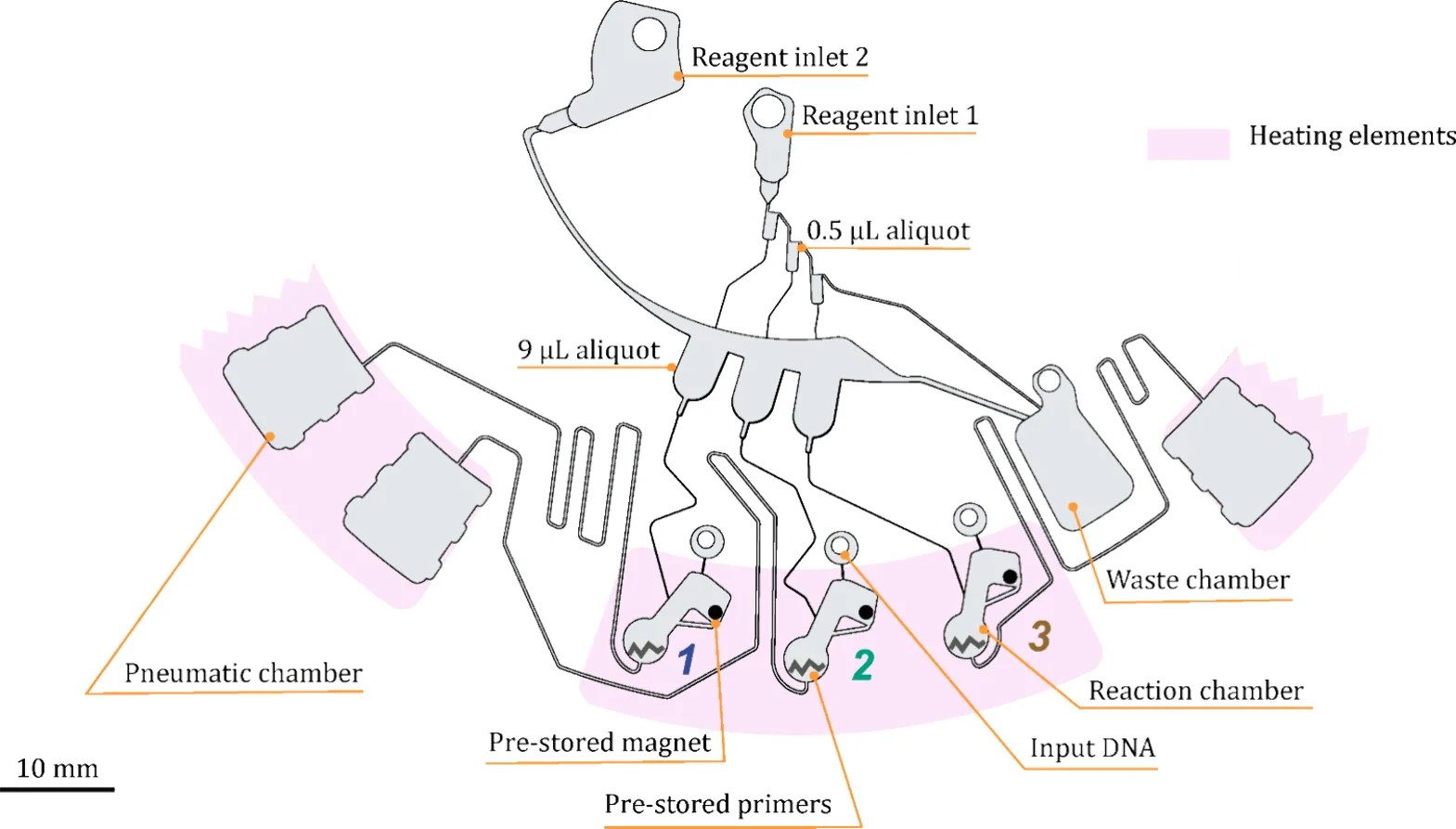
Layout of a single cartridge slice. Two-stage aliquoting structures (upper 0.5 μL and lower 9 μL, three chambers each) are fed by two reagent inlets, with excess liquid collected in a waste chamber. Biological reactions occur in the three reaction chambers, which receive samples through openings directly above and contain pre-stored primers and internal magnets. Pneumatic chambers provide bubble mixing before tagmentation and amplification, and assist liquid removal during binding and washing. The rotor’s heating elements sit directly below the reaction and pneumatic chambers. Reaction chambers are numbered 1–3 from left to right and color-coded blue (1), green (2), and brown (3).

### Bioinformatics

Pre-sequencing quality control measured DNA concentration on a Qubit 1.0 fluorometer and fragment size distribution on an Agilent Fragment Analyzer 5200. Libraries were then sequenced on an Illumina NextSeq 1000/2000 at the Research Center Borstel. Analysis used the MTBSeq workflow,^54^ which combined BWA^55^ and SAM tools^56^ for read alignment, PICARD-tools^57^ for duplicate-read removal, and the Genome Analysis Toolkit (GATK)^58^ for local realignment around insertions and deletions. MTBSeq reports three quality indicators: mapped read percentage, coverage depth, and coverage breadth percentage. Mapped read percentage is the fraction of reads with Phred quality >20 aligned to the target genome; high values indicate good library quality and low contamination, while low values suggest reads outside the MTBC. The threshold is ≥80%.^54^ Coverage depth is the average number of high-quality reads covering each nucleotide of the target genome;^59^ higher depth helps overcome sequencing errors, and a minimum of 50× is required to reliably detect MTBC mutations.^60,61^ Coverage breadth percentage is the fraction of the target genome covered by ≥4 reads with Phred quality >20; higher values increase the likelihood of detecting mutations at any position. The threshold is ≥95%.^62^

### Unit Operations for the Automated Workflow

The RoCM-LP platform uses six unit operations, with working principles shown in Figure 3 and Figure 4. Protocol parameters (rotation frequencies, temperatures, robot activities) are provided in Figure S1A.

(A) *The 0.5 μL Aliquoting Structure:* the SCARA robot picked up a pipette tip and aspirated reagent from the liquid storage, first mixing each tube with 5–10 aspirate/dispense cycles for homogeneity. The robot then moved to reagent inlet 1; the rotor stopped, and the robot then dispensed directly into the inlet. The rotor accelerated to 50 Hz at 20 Hz/s over 7 s, distributing reagent through three aliquoting chambers with a waste chamber collecting excess. A rectangular resistance channel (25 × 35 μm^2^, 3.3–9.1 mm long) at the bottom of each aliquoting chamber minimized pre-flow for accurate metering. The rotor then ramped to 60 Hz to transfer the aliquots to the 9 μL aliquoting structure and on to the reaction chambers. Each cycle delivers 0.5 μL, so 1 or 1.5 μL required 2 and 3 cycles, respectively. Between transfers, the robot discarded the used tip, picked up a new one, and parked near the rotor for the next cycle. Video S1 shows the robot–rotor aliquoting motion. The metering accuracy of the 0.5 μL aliquoting structure was reported in the Figure 5.
(B) *The 9 μL Aliquoting Structure:* This structure worked on the same principle as the 0.5 μL version but used a 70 × 70 μm^2^, 22.4 mm long rectangular resistance channel with lower hydraulic resistance^63^ and shorter transfer time. Reagents entered through reagent inlet 2. Binding and elution buffers (9 μL each) required one cycle, while each wash step used two cycles to deliver 18 μL. The metering accuracy of the 9 μL aliquoting structure was reported in the Figure 5.
(C) *Bubble-Based Liquid Mixing:* Heating the pneumatic chambers above room temperature generated positive pressure that drove hot air into the liquid in the reaction chambers; the bubbles rose by buoyancy and mixed the contents,^64^ with the rotor held at 10 Hz throughout.
(D) *Liquid Removal by Pneumatic Pressure:* To remove supernatant, the pneumatic chambers were heated to 100 °C for 2 min, then cooled to 35 °C for 5 min. Heating expelled expanded gas through the reaction chambers to atmosphere; subsequent cooling created suction that drew the liquid into the pneumatic chambers.^65^ There was almost no bead loss after liquid removal (Figure S11).
(E) *Internal Magnet Transfer:* The robot swapped the pipette tool for the magnet tool, which carries three 8 mm × 15 mm disc magnets (Figure S2), and positioned it 10 mm above the cartridge near the reaction chambers. With the rotor stopped, the robot followed the trajectory in Figure 4E (*e*1→*e*2→*e*3); magnetic coupling pulled the three internal magnets along the matching path (*i*1→*i*2→*i*3) to the bottom of each reaction chamber.
(F) *Bead Mixing:* The robot positioned the magnet tool 8 mm above the cartridge center. When a reaction chamber rotated away from the external magnets (Figure 4F, left), centrifugal force exceeded magnetic force and pushed the internal magnet outward; when it approached the external magnets (Figure 4F, right), magnetic force dominated and pulled the magnet inward. The resulting radial oscillation depended on rotation frequency, the number and size of internal and external magnets, and their relative positions. Beads followed the internal magnets through magnetic attraction, but shear and viscous drag from the liquid lagged them behind, dispersing the beads. Video S2 shows the mixing.

**Figure 3:**
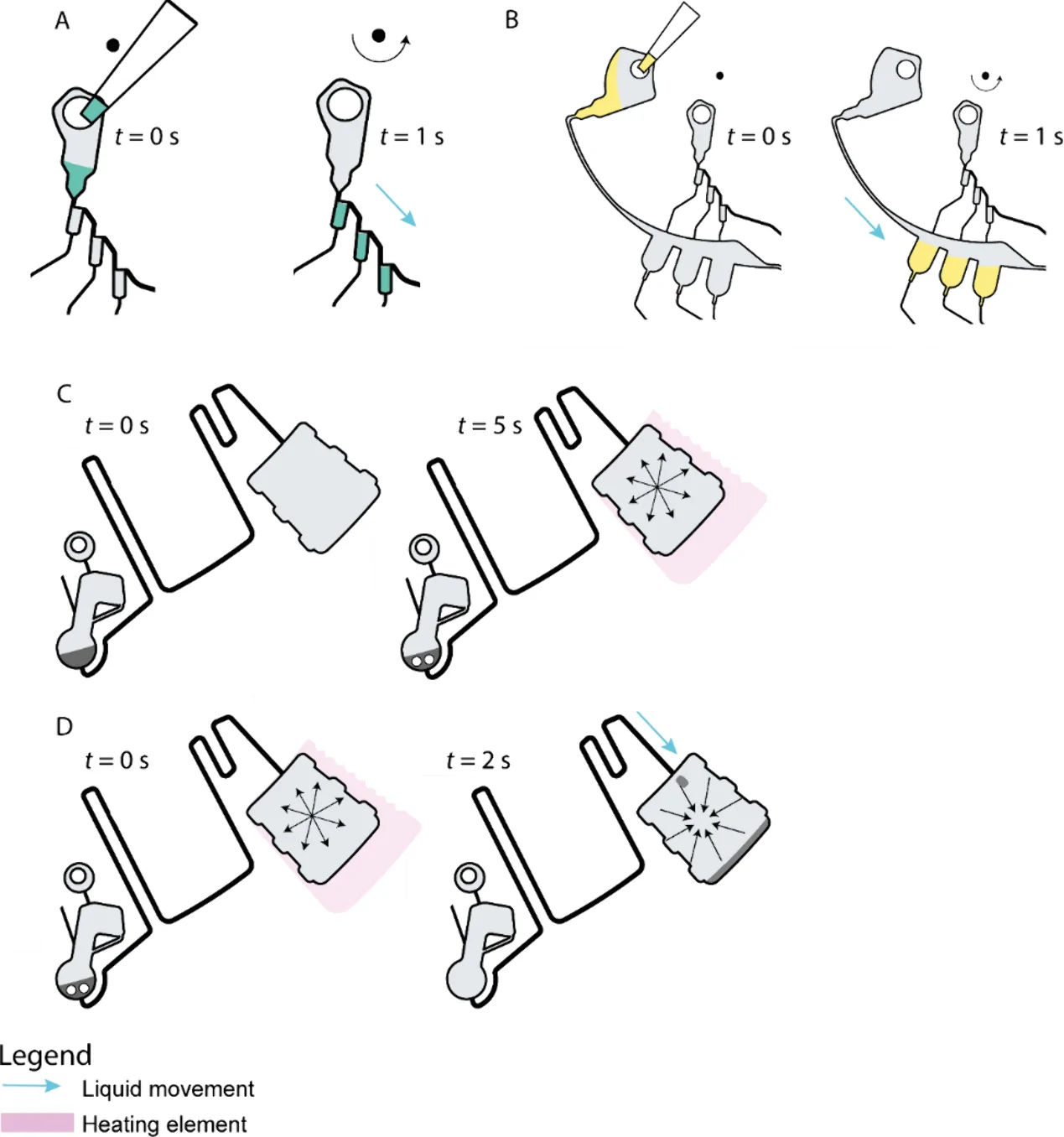
Unit operations on the RoCM-LP platform. (A) The 0.5 μL aliquoting structure meters small volumes (TD, ATM, NT, PCR Master Mix, water with 1% BSA) by resistance-based aliquoting; a rectangular resistance channel (25 × 35 μm^2^, 3.1–9.1 mm) minimizes pre-flow through the aliquoting chambers.^66^ (B) The 9 μL aliquoting structure uses the same principle for larger volumes (binding, washing, and elution buffers) through a 70 × 70 μm^2^, 22.4 mm resistance channel. (C) Bubble-based mixing homogenizes reagents before tagmentation and amplification: heating the pneumatic chambers raises their pressure, driving air bubbles through the liquid in the reaction chambers. (D) Pneumatic-pressure liquid removal (used for supernatant removal during binding and washing): heating pushes air through the reaction chambers, then cooling lowers the pressure and draws liquid back into the pneumatic chambers.

**Figure 4:**
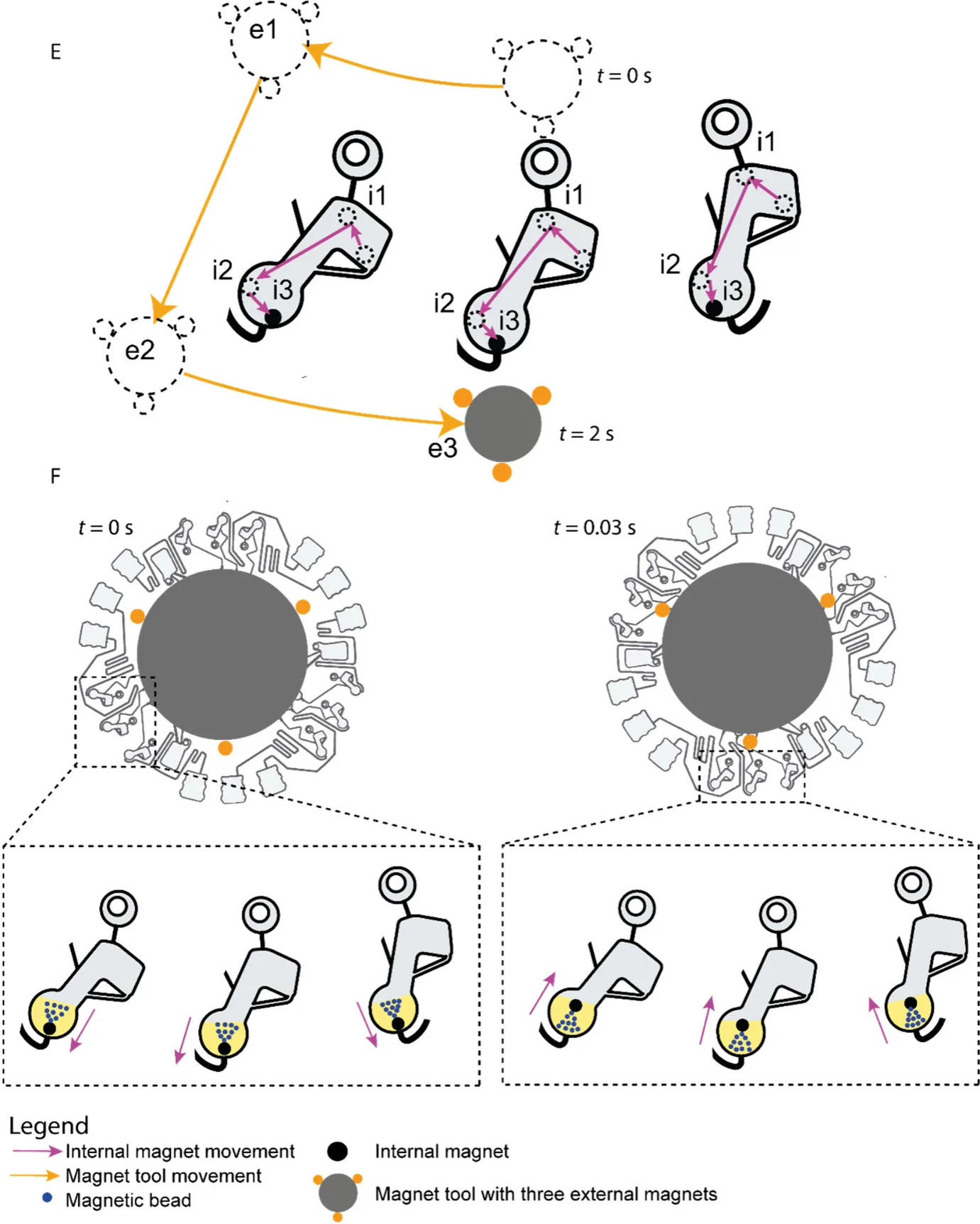
(E) Internal magnet transfer for bead mixing during binding and elution: the rotor stops and the magnet tool relocates internal magnets from their pre-stored locations to the bottoms of the reaction chambers, improving bead mixing and minimizing bead loss when liquid is drawn into the pneumatic chambers. (F) Bead mixing in binding and elution: the rotor spins the cartridge at 10–20 Hz while the robot holds the magnet tool above it. As the reaction chambers move away from the external magnets, centrifugal force pushes the internal magnets radially outward; as they re-approach, magnetic force pulls them inward. Beads track the internal magnets with a small lag from shear and viscous drag, dispersing continuously through the liquid.

**Figure 5:**
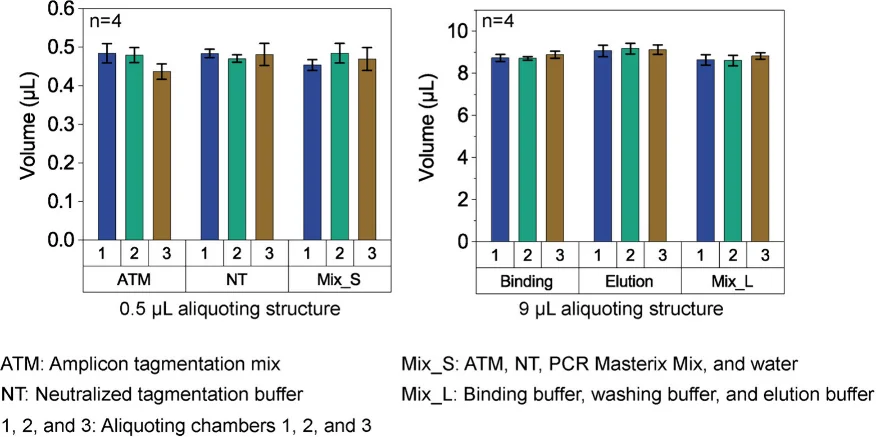
Metering accuracy of the 0.5 μL and 9 μL aliquoting structures. The 0.5 μL structure was tested with Amplicon tagmentation mix (ATM), neutralize tagment buffer (NT), and Mix_S (ATM, NT, PCR Master Mix, and water, dispensed sequentially). The 9 μL structure was tested with binding buffer, elution buffer, and Mix_L (binding buffer, 80% (v/v) ethanol (2×), and elution buffer, dispensed sequentially). Error bars represent the standard deviation of four measurements.

## Results and Discussion

### Automated Protocol

The RoCM-LP platform comprised three main parts: a SCARA robot, a rotor, and a centrifugal microfluidic cartridge.^1^ The robot supplied reagents and used the magnet tool to enhance bead mixing, while the rotor spun and heated the cartridge to drive aliquoting, tagmentation, amplification, binding, washing, and elution. The protocol used six unit operations (A–F) shown in Figure 3 and Figure 4; the rotation-frequency profile is given in Figure S1A. The 0.5 μL aliquoting structure (A) metered 0.5, 1, and 1.5 μL volumes for tagmentation and amplification. Before tagmentation, heating the pneumatic chambers to 60 °C drove bubble-based mixing (C); enzymatic fragmentation then ran at 58 °C for 5 min, with shake-mode mixing applied by ramping the rotation between 50 Hz (5 s) and 10 Hz over six cycles. Before amplification, the pneumatic chambers were heated from 60 °C to 70 °C for bubble-based mixing. During amplification, reaction-chamber temperature cycled through denaturation, annealing, and extension while the rotation again ramped between 50 and 10 Hz over 14 cycles. Temperature and frequency profiles for tagmentation and amplification are shown in Figure S1B. After amplification, the 9 μL aliquoting structure (B) metered 9 μL binding buffer, 18 μL washing buffer per wash, and 18 μL elution buffer. The SCARA robot then transferred internal magnets into the reaction chambers (E) and placed the magnet tool above the cartridge for bead mixing (F). Pneumatic-pressure liquid removal (D) drew binding and washing buffers from the reaction chambers into the pneumatic chambers. In the elution step, bead mixing (F) eluted DNA into the elution buffer.

### Measurement of Aliquoting Accuracy for 0.5 μL and 9 μL Aliquoting Structures

Validating the metering accuracy of the aliquoting structures is essential to ensure that correct reagent volumes are delivered in the automated workflow. For each structure, the volumes of three metering chambers were measured. The 0.5 μL structure was tested with ATM and NT, and the 9 μL structure with binding and elution buffers; each reagent was aliquoted four times. Mixed-reagent tests, in which different reagents were dispensed sequentially through the same structure, were also performed: ATM, NT, PCR Master Mix, and water were each metered once through the 0.5 μL structure (Mix_S), and binding buffer, washing buffer (2×), and elution buffer were metered in sequence through the 9 μL structure (Mix_L).

The advantage of the resistance-based design is that it meters repeatedly with liquids of differing properties while maintaining accuracy and requiring no calibration. Aliquoting structures based on hydrophobic^67^ or compressed- air^68^ valves also achieved high accuracy but were limited to a single use, as the wetting behavior of the valve changed after the first aliquoting step. Here, the resistance channel itself functions as the valve and minimizes pre-flow. Because hydrodynamic resistance scales with the fourth power of channel dimension but only linearly with liquid viscosity, metering accuracy is far less sensitive to liquid properties than in valve-based approaches.^69^ The coefficient of variation (CV) ranged from 1.8% to 6.4% for the 0.5 μL structure and from 0.9% to 2.9% for the 9 μL structure. The CV of the 0.5 μL structure was slightly higher than the 0.5% reported for the valve-based designs^67,68^ whose values were demonstrated only at 1.4–5 μL and for a single aliquoting step. The CV is also comparable to that of state-of-the-art pipetting robots such as the Hamilton MicroLab Prep^70^ or Agilent Bravo Handling Liquid^22^ which achieve approximately 5% at similarly small volumes.

### Comparison of Manual with Automated Tests

To benchmark RoCM-LP performance, sequencing libraries from four MTBC strains (S1–S4) prepared on the automated platform (one slice, three reaction chambers per strain) were compared with those from the manual reference workflow (triplicate per strain). Both workflows met all quality thresholds (Figure 6): coverage depth exceeded 50× in all cases and was consistently higher for the automated workflow (112× ± 11.95% vs 98× ± 39.74%). The average mapped-read percentage was slightly higher for the manual workflow (98.68% ± 0.34% vs 96.62% ± 3.53%; Welch t test, p = 0.03), whereas coverage breadth was comparable (98.33% ± 1.09% automated vs 98.50% ± 0.92% manual; p = 0.34).

**Figure 6:**
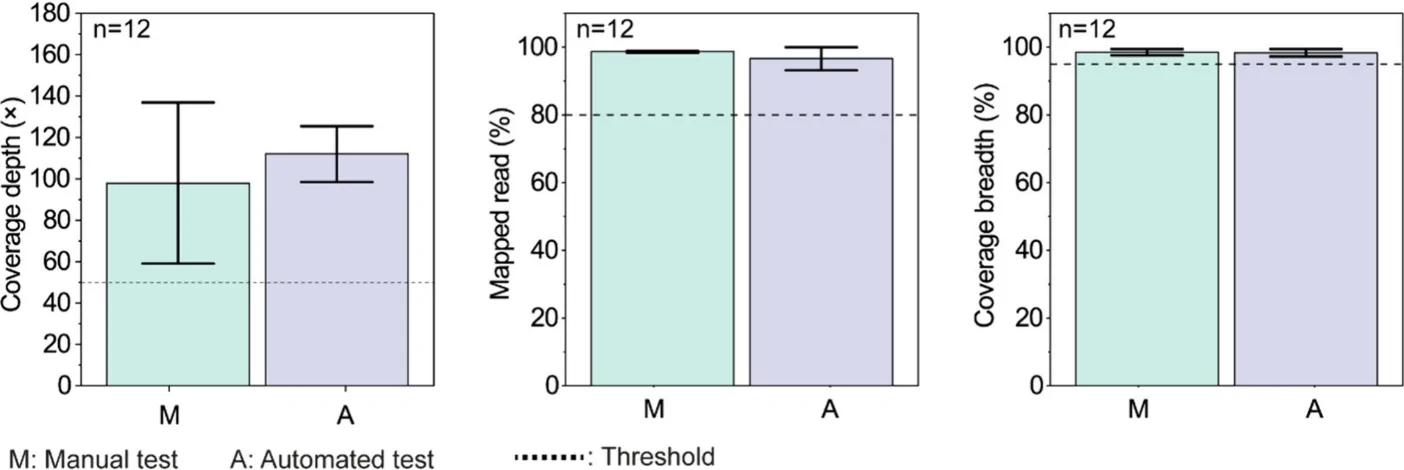
Sequencing results comparing automated library preparation on the RoCM-LP platform with manual preparation for four MTBC strains, using the reagent volumes listed in Table 1. A total of 12 samples from four MTBC strains were tested manually and automatically. Each strain was processed manually in triplicate and automatically in a single slice with three reaction chambers. Dashed lines mark the threshold for each quality indicator. The error bars in each graph represent the standard deviation.

Per-strain results are shown in Figure 7. Coverage breadth was similar across all four strains in both workflows, as were mapped-read percentages for S1 and S2. For S1, the mapped-read percentage was 98.74% ± 0.12% (manual) vs 96.75% ± 1.51% (automated), with coverage breadth of 99.0% ± 0% in both; for S2, 98.75% ± 0.03% vs 97.9% ± 0.3%, with coverage breadth of 97.0% ± 0% vs 96.7% ± 0%.

**Figure 7:**
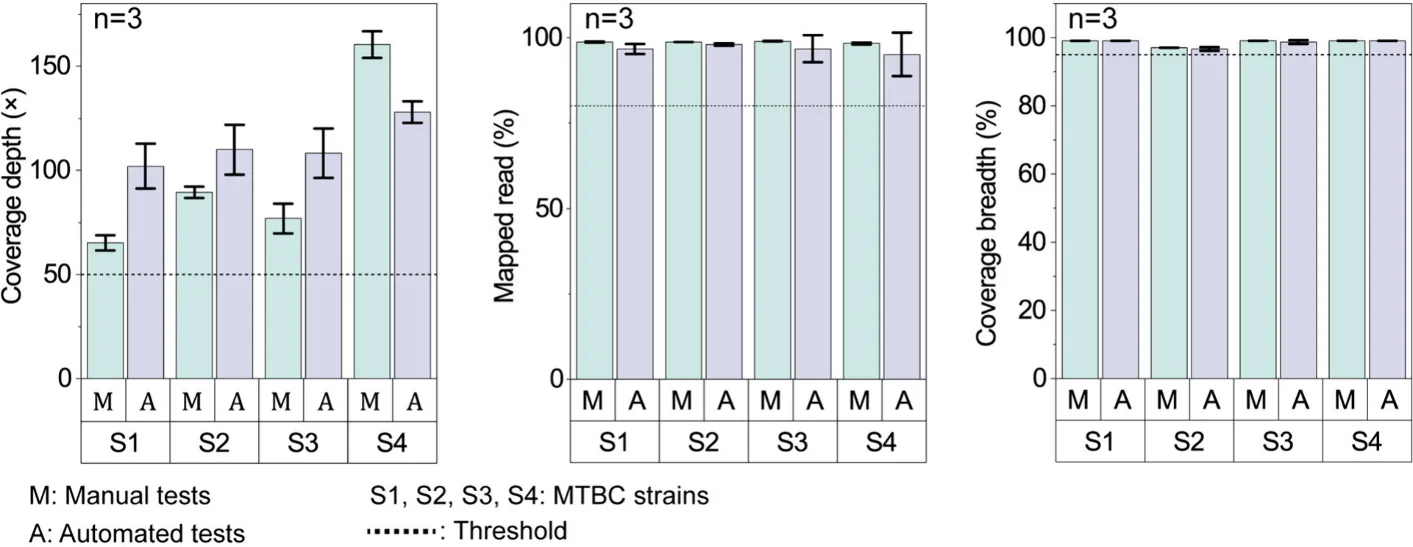
Detailed results of sequencing indicators for each MTBC strain in automated and manual tests from Figure 6. Dashed lines mark the threshold for each quality indicator. The error bars in each graph represent the standard deviation of the three data points.

Mapped read percentages for S3 and S4 in the automated workflow showed higher deviations than in the manual reference workflow: 98.9% ± 0.06% (manual) vs 96.7% ± 4.12% (automated) for S3; 98.3% ± 0.31% vs 95.1% ± 6.64% for S4, reflecting the differences in DNA fragment length distribution. The average fragment lengths (manual vs automated) were 446 bp ± 2.3% vs 498 bp ± 35.7% for S3 and 773 bp ± 3.51% vs 417 bp ± 21.9% for S4 (Figure S7). In S3, chamber 2 produced fragments averaging 700 bp against 421 bp (chamber 1) and 373 bp (chamber 3); in S4, chamber 1 produced 517 bp against 349 bp (chamber 2) and 387 bp (chamber 3) (Figures S4 and S5). Because longer fragments incur higher base-call error rates in Illumina sequencing^71^ these chambers yielded correspondingly lower mapped-read percentages. This variation originates at the tagmentation step, where fragment length depends on the transposase-to-DNA ratio.^49^

Three factors contribute to this variation. First, reagent miniaturization required manual pipetting of 0.5 μL of sample input, giving a variable DNA input, and aliquoting of 0.5 μL of transposase in ATM per reaction. The manufacturer-specified imprecision is ±8% at 0.5 μL vs ±1% at 10 μL respectively^72^ and is higher still depending on operator skill and experience.^73,74^ To assess the impact of handling small liquid volumes, libraries were also prepared manually at miniaturized volumes and compared with those from the manual reference workflow (unchanged volumes) and the automated workflow (Figure S9). Two of the three samples in the manually miniaturized workflow failed: most DNA fragments were approximately 200 bp, whereas fragments ranged from 400 to 1000 bp in both the automated and the manual reference workflows.

Second, protein adsorption onto the COC cartridge surfaces during aliquoting can hinder enzymatic activity during tagmentation.^75,76^ Adsorption is driven mainly by dehydration forces and hydrophobic binding and may cause protein loss, reduced activity, or structural change.^77^ Adsorption of transposase in the transferred 0.5 μL of ATM would therefore render tagmentation less effective. Third, S3 and S4 differ genomically from S1 and S2. Both are drug-resistant lineage 2 (Beijing family) strains: S3 is an MDR strain (4,245,814 bp) carrying 1396 SNPs^45^ and S4 an XDR strain (4.2–4.4 Mbp) with multiple insertions and deletions conferring resistance to several TB drugs.^46,47^ In contrast, S1 is the H37Rv reference strain (4,417,942 bp)^43^ and S2 an attenuated BCG vaccine strain with a virulence-reducing deletion (4,374,522 bp).^44^ S3 and S4 both carry a deletion of RD105 and potentially of RD142, RD150, and RD181, which remain intact in S1 and S2.^78^ Such structural differences may directly affect transposase binding to DNA.^79^

DNA input in the automated workflow was reduced 10-fold relative to the manual workflow, so 14 amplification cycles were used instead of 12 to increase library yield. The total yield of DNA in libraries in both workflows before normalization for sequencing was reported in Figure S10. Because additional cycles risk over-amplifying some genomic regions while leaving others uncovered, GC content and whole-genome coverage were checked for each strain. GC distribution metrics: The GC content percentage of total reads in manual and automated workflows was comparable in four MTBC strains: 65.523% ± 0.67% and 65.51% ± 0.68% (Figure S8). No amplification bias was detected for S1, S2, or S3. One S4 replicate indicated bias (Figure S34). This replicate was in chamber 1, which had the longest fragment distribution (517 bp vs 349 and 387 bp) and the lowest mapped-read percentage (87.8% vs 98.9% and 99.5%). The mismatch may therefore reflect a combination of amplification bias and the fragment-length variance described above. As MTBC detection relies on multiple regional sequencing markers rather than a single nucleotide, strain identification remains possible despite this discrepancy.

MTBC strain identification requires at least 80% of reads to map to the reference genome. The automated libraries comfortably exceeded this threshold, with an average mapped-read percentage of 96.6%. Overall, although the automated results deviated slightly from the manual reference, all quality criteria were met and four MTBC strains were reliably identified. The mutation detection concordance between the reference genome and the results in both workflows can be found in Table S4.

### Reproducibility of Reaction Chambers 1–3 on a Single Slice

To assess whether the RoCM-LP platform delivers reproducible results across sample inputs, four slices were used to process four MTBC strains (S1–S4), one strain per slice. The average coverage depths of 102× ± 10.48% (S1), 110× ± 10.94% (S2), 108× ± 10.83% (S3), and 128× ± 4.03% (S4) confirmed that the automated libraries provided sufficient sequencing data for all four strains (Figure 8). The average mapped-read percentages were 96.8% ± 1.51% (S1), 97.9% ± 0.38% (S2), 96.7% ± 4.12% (S3), and 95.1% ± 6.64% (S4), all well above the 80% threshold. The average coverage breadth percentages were high, with 99% ± 0% (S1), 96.6% ± 0.59% (S2), 98.7% ± 0.59% (S3), and 99% ± 0% (S4), each exceeding the 95% threshold. In the automated tests, S4 had the lowest mapped-read percentage (95.1%) and S2 the lowest coverage breadth (96.6%); the same strain-level pattern was observed in the manual tests, where S4 (98.3% ± 0.31%) and S2 (97.0% ± 0%) again ranked lowest. Because this strain-dependent ranking appeared in both workflows, the variance is most likely intrinsic to the input material: differences in DNA sequence, secondary structure, and fragment-size distribution among the four MTBC strains.^79,80^ In summary, each slice produced high-quality, comparable libraries across all four MTBC strains.

**Figure 8:**
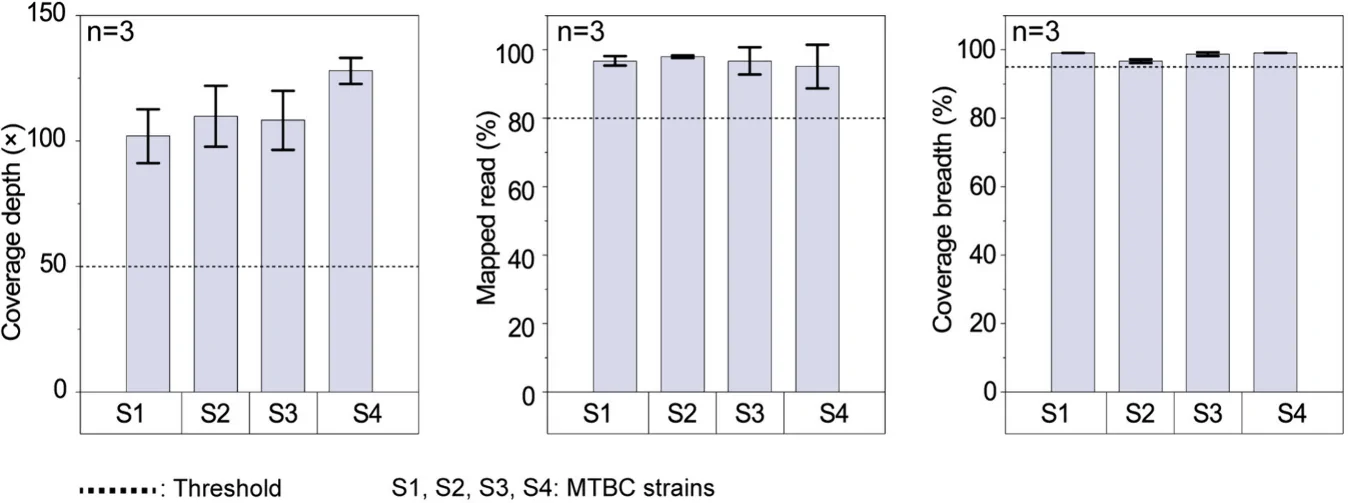
Sequencing results for libraries generated within a single cartridge slice for four MTBC strains (S1, S2, S3, and S4). Dashed lines mark the threshold for each quality indicator. The error bars in each graph represent the standard deviation.

### Analysis of Cross-Contamination between Reaction Chambers 1–3

Because the aliquoting structures share a fluidic connection, the three reaction chambers of a single slice are not fully independent (Figure 2), so cross-contamination between neighboring chambers had to be ruled out experimentally. As shown in Figure 9, the average coverage depth was 0.3× ± 149% for the three negative controls versus 157.2 × ± 15.96% for the positive controls, indicating that essentially no bacterial DNA was present in the negative chambers while the positive chambers yielded ample genomic reads. The mapped-read and coverage-breadth percentages followed the same pattern: 0.5% ± 95.6% vs 98.7% ± 0.99% and 0.1% ± 168% vs 99.3% ± 0.13%, respectively, for negative versus positive controls. The positive-control libraries cleared every sequencing-quality threshold, while the residual reads in the negative chambers were too few to indicate carryover (Figure 10) and are instead consistent with index hopping during sequencing, a known artifact of Illumina platforms.^81^ Index hopping occurs when residual adapters and free primers in a library bind incorrectly to fragments during cluster generation, causing the index of one sample to be misassigned to another in multiplex sequencing; the resulting misassigned-read rate can reach 10% on Illumina platforms.^82^ In this work, the Nextera XT DNA library preparation protocol, UD Indexes, and a high-throughput sequencer (NextSeq 1000/2000) were used. Therefore, the average rate of index hopping for this combination has been reported as 0.68 ± 0.19%^83^ (Table S3). The mapped read and coverage breadth percentages in negative controls were in the range of 0.1–0.5%, lower than 0.68%. Furthermore, the DNA concentration of the three NTC samples was also measured by Qubit fluorometer using the dsDNA High Sensitive Assay Kit.^84^ All three NTC samples had DNA concentrations below the detection limit of the assay (<0.5 pg/μL). Because positive and negative libraries were pooled in the same sequencing run, the 0.5% mapped-read percentage in the negative controls is fully explained by index hopping rather than on-chip carryover. Together, these results confirm that no cross-contamination occurred between reaction chambers across all three tests.

**Figure 9:**
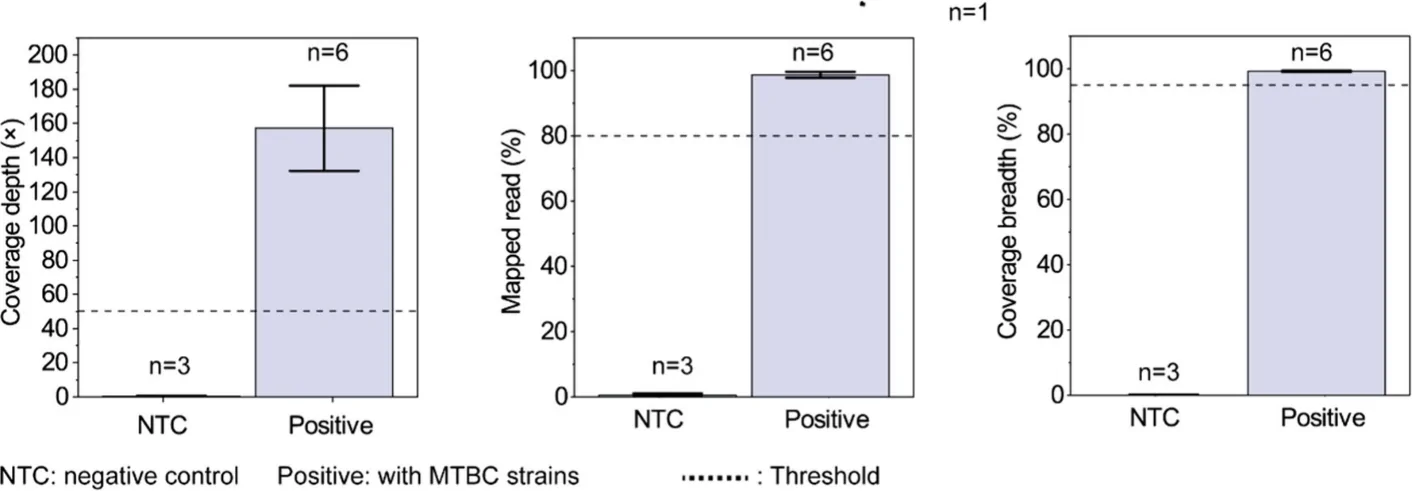
Sequencing results from the cross-contamination test across three reaction chambers. Three slices were processed in total. In each slice, one chamber received only nuclease-free DNA/RNA water (negative control) while the other two received DNA from MTBC strain S1 (positive controls); the negative control was chamber 1 in slice 1, chamber 2 in slice 2, and chamber 3 in slice 3. In total, there were 3 negative controls and 6 positive controls. Dashed lines mark the threshold for each quality indicator. The error bars in each graph represent the standard deviation.

**Figure 10:**
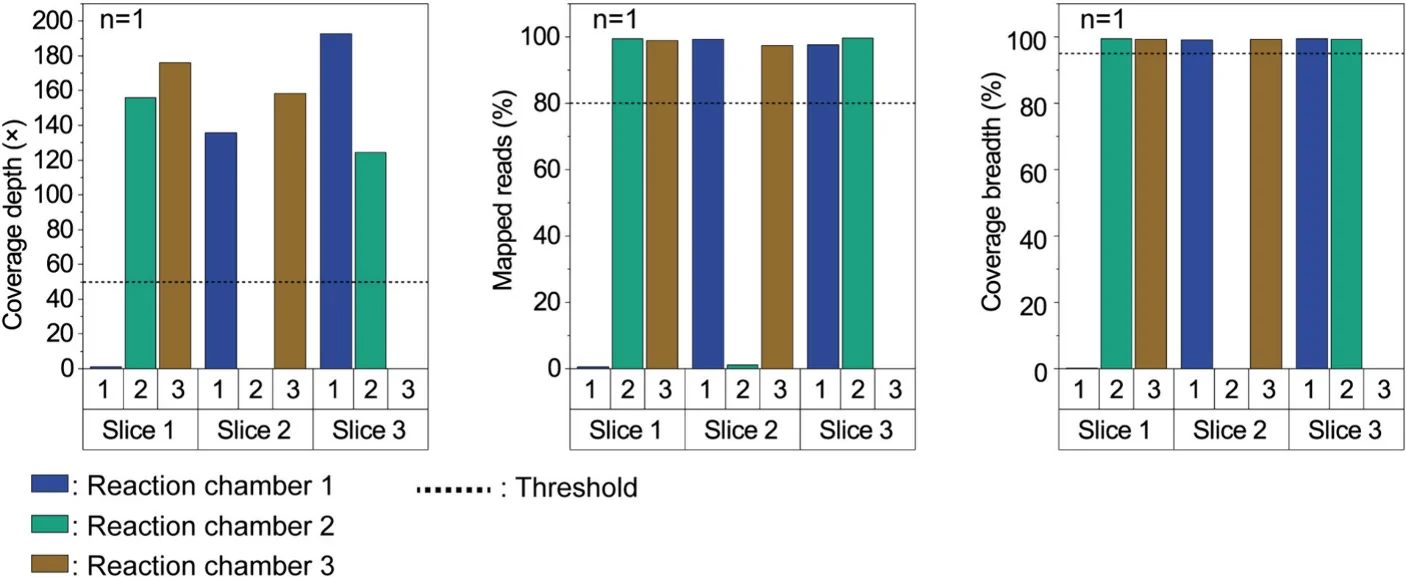
Detailed sequencing results in slice 1, slice 2, and slice 3 for cross-contamination tests (Figure 9). The negative control was chamber 1 in slice 1, chamber 2 in slice 2, and chamber 3 in slice 3. Dashed lines mark the threshold for each quality indicator; bar colors denote reaction chambers 1–3.

### Reproducibility of Reaction Chambers 1–3 Across Slices 1–3

To verify whether the platform delivers nine reproducible libraries from a single sample input, three slices were each loaded with the same MTBC strain (S1) and processed in parallel. Figure 11 shows that all three slices generated sufficient sequencing data, with average coverage depths of 102× ± 10.48% (slice 1), 109× ± 14.57% (slice 2), and 130× ± 11.7% (slice 3). Average coverage breadth percentage was 99% ± 0% across all three slices, and average mapped-read percentages were comparable at 96.75% ± 1.51% (slice 1) and 98.74% ± 0.22% (slice 2), and 98.66% ± 0.99% (slice 3). The slightly lower mapped-read percentage in slice 1 reflected its longer average DNA fragment size: 924 bp, compared with 552 bp in slice 2 and 565 bp in slice 3 (). As noted earlier in the manual-versus-automated comparison, longer fragments yield lower mapped-read percentages, plausibly due to pipetting variability and protein adsorption on the cartridge surface. In conclusion, the three slices performed equivalently and exceeded all sequencing-quality thresholds, confirming that the RoCM-LP platform reliably generates nine high-quality libraries in parallel and is suitable for scalable, reproducible sample processing. One limitation of the RoCM-LP platform is that it processes samples in multiples of three (3, 6, or 9) in parallel rather than individually. This constraint arises from the design of the heating elements in the rotor. Revising this design would allow each reaction chamber to be controlled and therefore processed independently. In practice, however, this limitation has little impact: bacterial genomes are short, and sequencing is typically performed on multiple samples simultaneously, so single-sample runs are neither cost-effective nor representative of routine daily use.

**Figure 11:**
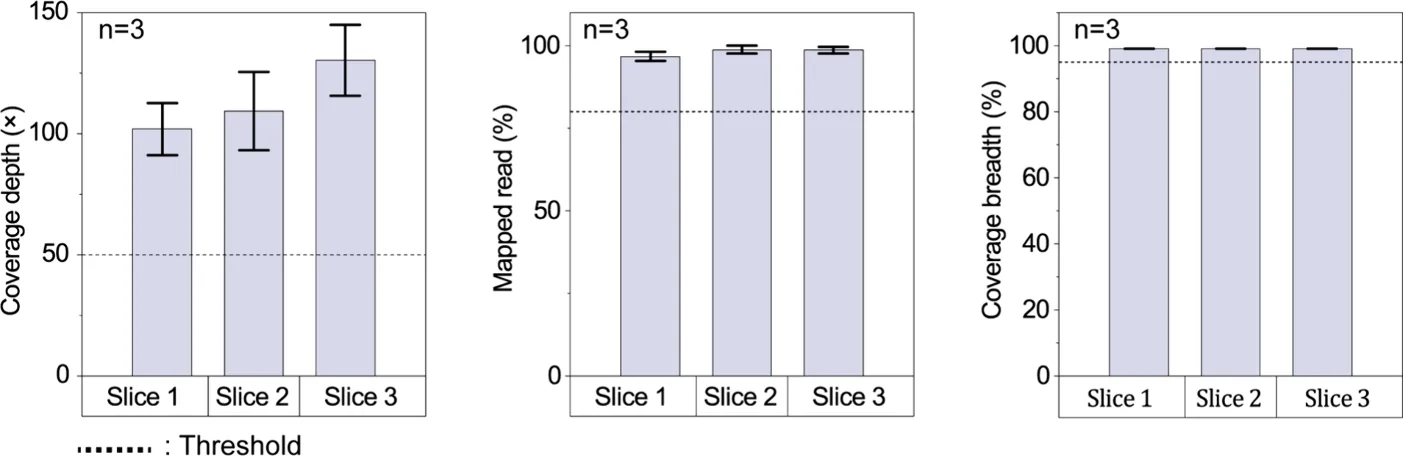
Sequencing results from the reproducibility test of three reaction chambers across three slices, all loaded with the same MTBC strain (S1). Dashed lines mark the threshold for each quality indicator. The error bars in each graph represent the standard deviation of the three data points.

Furthermore, the RoCM-LP platform does not include an automated quality control of the samples (analysis of DNA concentration and fragment length distribution). The RoCM-LP platform could further reduce downstream analysis required from users by integrating on-board DNA quantification. qPCRs have already been demonstrated with a similar turntable setup in multiple applications.^85^ An individual slice for QC based on qPCR similar to LabSlice^86^ could be used for precise quantification in a centrifugal microfluidic setup.

## Conclusions

This work demonstrates that the RoCM-LP platform reliably prepares nine high-quality sequencing libraries in parallel, with sufficient coverage depth and high mapped-read and coverage-breadth percentages, for four different MTBC strains, a throughput well suited to decentralized laboratories. Investment costs of $50k–70k are comparable to or lower than those of most medium- and high-throughput systems (Table 2). Furthermore, the costs per sample were also substantially lower: a tenfold reduction in reagent consumption relative to the manual reference workflow was achieved without loss of sequencing quality, in contrast to platforms that rely on costly consumables such as cartridges and pipette tips (Table 2). The RoCM-LP platform also increased the degree of automation with a minimum of three manual handling steps, namely reagent preparation, sample loading, and library retrieval. This high degree of automation is on par with most other available platforms (Table 2).

**Table 2:** Comparison of the Different Platforms in Terms of Four Requirements (Sample Throughput, Investment Costs, Costs Per Sample, and Degree of Automation) for NGS Application in Decentralized Laboratories

system	examples	sample throughput	investment costs	costs per sample	degree of automation
high-throughput systems	the Agilent Bravo Platform, the Beckman Coulter Biomek i7, the MGISP-960 from MGI	96–384 samples per run	$100k–200k	high: when the platform is used to process a low number of samples in decentralized laboratories due to required disposables	high: it requires only 3 manual handling steps such as sample input, reagent preparation, and library retrieval
	Mosquito Gen3 from SPTLabTech	up to 384 samples per run	$90k–140k	high: it requires expensive disposable pipette tips	low: it requires more than 4 manual handling steps including sample input, reagent preparations, carrying microwell plates multiple times over to thermal cyclers, and library removal
	MOgene Fluidigm Juno	Up to 384 samples per run	$20k–60k	high: it requires expensive cartridges ($300–$1000 per cartridge for one run)	high: it requires only 3 manual handling steps

medium-throughput systems	MagicPrep NGS from Tecan	8 samples per run	$50k–70k	high: it requires expensive cartridges ($400 for one cartridge per run)	low: it requires more than 6 manual handling steps with additional sample fragmentation steps
	BioQule NGS system from Revvity	8 samples per run	$50k–70k	high: it requires expensive cartridges ($400–$500 for one cartridge)	high: it requires 3 manual handling steps

low-throughput systems	centrifugal microfluidic cartridge	2 samples per run	$25k–30k	low: it requires low-cost cartridges	high: it requires 3 simple manual handling steps

our approach	RoCM-LP platform: pipetting robot and centrifugal microfluidics	3–9 samples per run	$50k–70k	low: it requires low-cost consumables: <$10 manufacturing cost for one cartridge and $5 for reagent cost per sample; total cartridge cost to process 9 samples in parallel is <$ 60; detailed cost calculation can be found in cost calculation (Supporting Information)	high: it requires 3 manual handling steps

The current microfluidic design forces each run to comprise either a triplicate or three different samples. A revised turntable with smaller heating elements would allow each sample to be controlled independently, increasing the platform’s flexibility. Investment costs remain elevated because production volumes are low; mass-manufactured components and a simplified design would lower them further and broaden access for decentralized laboratories. Costs per sample can be reduced still further through miniaturization once limiting factors such as manual pipetting of 0.5 μL of sample liquid and protein adsorption can be addressed. To address the manual pipetting, an additional 0.5 μL aliquoting structure at the sample inlet would reduce manual handling error: a pipetting robot could dispense a bulk sample volume, from which 0.5 μL could then be aliquoted precisely and reliably. This could further reduce the manual handling steps from three to two (reagent preparation and library retrieval). To address protein adsorption, methods such as plasma treatments,^87^ UV oxidation,^88^ and dip coating^75^ have been reported to render the surfaces hydrophilic, preventing the hydrophobic interaction with proteins.

The RoCM-LP platform should be evaluated in regions where tuberculosis remains a major health burden, such as countries in Africa and Central Asia, and its application to other relevant scenarios such as monitoring antimicrobial resistance (AMR)^89^ and foodborne pathogens (Salmonella, Listeria)^90^ should be investigated.

Next, the cartridge in this work was designed for Illumina’s Nextera XT protocol, which involves tagmentation, amplification, and a single bead cleanup. The cartridge has a simple design comprising double aliquoting structures, a single one-pot reaction chamber, and pneumatic chambers for liquid handling. The 0.5 μL and 9 μL metering chambers cover volumes of 0.5–5 μL and 9–90 μL, respectively. The reaction chamber accommodates up to 350 μL and can be controlled from 10 °C to 120 °C, spanning the typical range for biological reactions, while the pneumatic chambers handle 800–1200 μL. Similar workflows, such as seqWell’s ExpressPlex 2.0,^91^ can therefore be accommodated on the same cartridge design. The main limitation is that only a single bead cleanup step is possible, whereas protocols such as Illumina DNA Prep^92^ require two steps with different beads. Storing two bead sets separately would therefore broaden the applicability of the platform, but would require a substantial redesign.

## Supplementary Material







## Notes

Description of the parameter protocol for the manual reference workflow and the automated workflow, a CAD design for the magnet tool in the RoCM-LP platform, a CAD design for isothermal insulation cover for the cartridge, DNA fragment size distributions to explain data in results and discussions (PDF)
Recording of the movements of SCARA robot and rotor in the RoCM-LP platform to aliquot liquids (MP4)
Video S2: Recording of bead mixing. Declaration of Generative AI and AI-assisted Technologies in the Writing Process Manuscript editing was supported by Claude (Anthropic) (MP4)
