# Supplementary material for: Robotic Centrifugal Microfluidic Automation Enables Library Preparation of Nine Samples with a Tenfold Reduction in Reagent Consumption for Decentralized Sequencing
Source: Anal Chem. 2026 Sep 21;98(39):28917–31. doi: 10.1021/acs.analchem.6c03501 (PMC13647976; doi:10.1021/acs.analchem.6c03501)
Supplement: Supplementary file 1 [file ac6c03501_si_001.pdf]

Supporting Information

Robotic centrifugal microfluidic automation enables library preparation of nine samples with a tenfold reduction in reagent consumption for decentralized sequencing

Trong Nguyen[a,b], Viola Dreyer[c,d], Yumi Kaku[a], Peter Juelg[a,b], Tobias Hutzenlaub[a,b], Stefan Niemann[c], Nils Paust[a,b], and Jacob Friedrich Hess[a,b](*)

[a]Hahn-Schickard, 79110 Freiburg, Germany; [b]Laboratory for MEMS Applications, IMTEK – Department of Microsystems Engineering, University of Freiburg, 79110 Freiburg, Germany; [c]Research Center Borstel, Leibniz Lung Center, 23845 Borstel, Germany; [d]German Center for Infection Research (DZIF), 20359 Hamburg, Germany

(*) Corresponding author

Table of Contents

Table S1: Complete automated protocol for the RoCM-LP platform	S2
Table S2: Complete parameter protocol for the manual reference workflow.	S6
Table S3: The reported index hopping rate with non-redundant dual index.	S8
Table S4: The mutation detection concordance in the automated and manual workflows.	S8
Figure S1: Illustration of automation protocol for the rotation frequency and temperature	S9
Figure S2: CAD Design for the magnet tool.	S10
Figure S3: CAD design of a thermal insulation cover.	S10
Figure S4: The DNA fragment size distribution in the automated tests for MTBC strain S3.	S11
Figure S5: The DNA fragment size distribution in the automated tests for MTBC strain S4.	S11
Figure S6: The DNA fragment size distribution in reproducibility tests for three slices.	S12
Figure S7: The average DNA fragment size between manual and automated workflow.	S13
Figure S8: The average GC content of total reads between manual and automated workflow.	S13
Figure S9: The DNA fragment size distribution in miniaturized manual workflow	S14
Figure S10: DNA yield post library preparation and before normalization for sequencing.	S14
Figure S11: Magnetic bead retention in reaction chambers after binding and washing steps.	S15
Figure S12: Pre- and post-calibration temperature data from six heating elements	S16
Cost Calculation (Tables S5 – S11).	S17

Whole Genome Coverage for four MTBC strains (Figures S13 – S36). S20
Reference link S30

Table S1: Complete protocol for the RoCM-LP platform with rotations, temperatures, and time parameters.

Step number	Step description	Frequency	Duration	Acceleration/ Deceleration	Reaction chambers	Pneumatic chambers
1	Insert 0.5 μL of DNA sample into the reaction chamber				30 ˚C	30 ˚C
2	Bring sample down	10 Hz	20 s	10 Hz/s	“	“
3		60 Hz	30 s	5 Hz/s	“	“
4	Stop rotation	0 Hz	-	20 Hz	“	“
5	Robot inserts Tagmentation Buffer into the cartridge inlet 1	“	-	-	“	“
6	Aliquot Tagmentation Buffer	50 Hz	7 s	20 Hz/s	“	“
7		60 Hz	40 s	5 Hz/s	“	“
8	Repeat steps 4–7				“	“
9	Stop rotation	0 Hz	-	20 Hz	“	“
10	Robot inserts Amplicon Tagmentation Mix into the reagent inlet 1	“	-	-	“	“
11	Aliquot Amplicon Tagmentation Mix	50 Hz	7 s	20 Hz/s	“	“
12		60 Hz	40 s	5 Hz/s	“	“
13	Reduce frequency for bubble mixing	10 Hz	70 s	10 Hz/s	“	60 ˚C
14	Bring liquids down after mixing	50 Hz	60 s	5 Hz/s	“	“
15	Reduce frequency for tagmentation	10 Hz	10 s	10 Hz/s	“	“
16	Tagmentation	“	30 s	“	58 ˚C	“
17	Wait	“	50 s	“	“	“
18	Increase frequency	50 Hz	5 s	5 Hz/s	“	“

19	Repeat steps 17 and 18 six times	-	-	-	“	“
20	Cool down to stop tagmentation	50 Hz	10 s	5 Hz/s	10 ˚C	“
21	Bring reaction chambers to room temperature	“	60 s	“	30 ˚C	“
22	Reduce frequency	10 Hz	10 s	10 Hz/s	“	“
23	Stop rotation	0 Hz	-	-	“	“
24	Robot inserts Neutralize Tagmentation Buffer into the cartridge inlet 1	-	-	-	“	“
25	Aliquot Neutralize Tagmentation Buffer	60 Hz	40 s	10 Hz/s	“	“
26	Incubate	10 Hz	300 s	5 Hz/s	“	“
27		50 Hz	140 s	“	“	“
28		40 Hz	20 s	“	“	“
29		30 Hz	“	“	“	“
30		20 Hz	“	“	“	“
31		10 Hz	“	“	“	“
32	Stop rotation	0 Hz	-	“	“	“
33	Robot inserts PCR Master Mix into the cartridge inlet 1	-	-	-	“	“
34	Aliquot PCR Master Mix	50 Hz	7 s	20 Hz/s	“	“
35		60 Hz	140 s	5 Hz/s	“	“
36	Reduce frequency	40 Hz	10 s	5 Hz/s	“	“
37		20 Hz	“	“	“	“
38		10 Hz	“	“	“	“
39	Repeat steps 32–38 three times	-	-	-	“	“
40	Stop rotation	0 Hz	-	“	“	“
41	Robot inserts Water (1 µg/µL BSA) into the cartridge inlet 1	-	-	-	“	“
42	Aliquot water	50 Hz	7 s	20 Hz/s	“	“
43		60 Hz	140 s	5 Hz/s	“	“
44	Reduce frequency	40 Hz	10 s	5 Hz/s	“	“
45		20 Hz	“	“	“	“
46		10 Hz	“	“	“	“
47	Repeat steps 40–46 two times	-	-	-	“	“

48	Robot takes a magnet tool				“	“
49	Robot uses the magnet tool to move internal magnets into a pre-stored position	-	-	-	“	“
50	Bubble mix	10 Hz	70 s	10 Hz/s	“	70 ˚C
51	Bring liquids down after mixing	50 Hz	60 s	5 Hz/s	“	“
52	Reduce frequency for PCR	10 Hz	10 s	10 Hz/s	“	“
53	Pre-annealing	“	180 s	“	77 ˚C	“
54	Pre-denaturation	“	90 s	“	100 ˚C	“
55	PCR-denaturation	“	45 s	“	“	“
56	PCR-binding	“	60 s	“	58 ˚C	“
57	PCR-annealing	“	60 s	“	77 ˚C	“
58	Increase frequency	50 Hz	5 s	5 Hz/s	“	“
59	Repeat 14 PCR cycles (steps 55–58)					
60	Final-annealing	10 Hz	300 s	10 Hz/s	“	“
61	Stop PCR	“	80 s	“	30 ˚C	
62	Increase frequency	50 Hz	20 s	5 Hz/s	“	“
63	Reduce frequency	10 Hz	10 s	10 Hz/s	“	“
64	Stop rotation	0 Hz	-	“	“	“
65	Robot uses the magnet tool to move internal magnets from the pre-stored positions to the bottom of the reaction chambers	-	-	-	“	“
66	Increase frequency	10 Hz	-	10 Hz/s	“	“
67	Stop rotation	0 Hz			“	“
68	Robot inserts Binding Buffer into the cartridge inlet 2	-	-	-	“	“
69	Aliquot Binding Buffer	50 Hz	7 s	20 Hz/s	“	“
70		60 Hz	60 s	5 Hz/s	“	“
71		30 Hz	10 s	“		
72		15 Hz	10 s	20 Hz/s		
73	Bubble Mix	“	70 s	“	“	100 ˚C
74	Increase frequency	40 Hz	5 s	“	“	“
75	Robot gets the magnet tool	“	“	“	“	“
76	Mixing beads	10 Hz	20 s	“	“	“
77		15 Hz	20 s	“	“	“

78		20 Hz	20 s	“	“	“
79	Repeat steps 76–78 seven times	-	-	-	“	“
80	Robot removes the magnet tools away from the cartridge to stop mixing	-	-	-	“	“
81	Incubate	20 Hz	120 s	10 Hz/s	“	“
82		50 Hz	20 s	“	“	“
83	Remove Binding Buffer	“	10 s	“	“	35 °C
84		1 Hz	180 s	5 Hz/s	“	“
85	Stop rotation	0 Hz	-	“	“	“
86	Robot inserts Washing Buffer (80% Ethanol) into the cartridge inlet 2	-	-	-	“	“
87	Aliquot Washing Buffer	50 Hz	7 s	20 Hz/s	“	“
88		60 Hz	200 s	5 Hz/s	“	“
89	Reduce frequency	10 Hz	-	5 Hz/s	“	“
90	Repeat steps 85–89 two times	-	-	-	“	“
91	Incubate	10 Hz	30 s	10 Hz/s	“	“
92		50 Hz	10 s	“	“	“
93		20 Hz	10 s	“	“	“
94	Remove Washing Buffer	“	40 s	“	“	100 °C
95		5 Hz	60 s	5 Hz/s	“	35 °C
96	Increase frequency	50 Hz	20 s	10 Hz/s	“	“
97	Reduce frequency	10 Hz	-	-	“	“
98	Stop rotation	0 Hz	-	“	“	“
99	Robot inserts Washing Buffer (80% Ethanol) into the cartridge inlet 2	-	-	-	“	“
100	Aliquot Washing Buffer	50 Hz	7 s	20 Hz/s	“	“
101		60 Hz	200 s	5 Hz/s	“	“
102	Reduce frequency	10 Hz	-	5 Hz/s	“	“
103	Repeat steps 98–102 two times	-	-	-	“	“
104	Incubate	10 Hz	30 s	10 Hz/s	“	“
105		50 Hz	10 s	“	“	“
106		20 Hz	10 s	“	“	“

107	Remove Washing Buffer	“	40 s	“	“	100 ˚C
108		5 Hz	60 s	5 Hz/s	“	35 ˚C
109	Increase frequency	50 Hz	20 s	10 Hz/s	“	“
110	Reduce frequency	10 Hz	-	-	“	“
111	Stop rotation	0 Hz	-	“	“	“
112	Robot inserts Elution Buffer into the cartridge inlet 2	-	-	-	“	“
113	Aliquot Elution Buffer	20 Hz	7 s	20 Hz/s	“	“
114		60 Hz	20 s	5 Hz/s	“	“
115	Reduce frequency	10 Hz	-	5 Hz/s	“	“
116	Repeat steps 111–115 two times	-	-	-	“	“
117	Incubate	10 Hz	30 s	10 Hz/s	“	“
118		50 Hz	10 s	“	“	“
119		20 Hz	10 s	“	“	“
120	Robot gets the magnet tool for bead mixing					
121	Increase frequency	40 Hz	5 s	5 Hz/s	“	“
122	Mix beads	10 Hz	20 s	20 Hz/s	“	“
123		15 Hz	“	“	“	“
124		20 Hz	“	“	“	“
125	Repeat steps 122–124 six times	-	-	-	-	-
126	Robot removes the magnet tools away from the cartridge to stop mixing	-	-	-	-	-
127	Incubate	10 Hz	120 s	10 Hz/s	“	“
128	Stop rotation to remove libraries out	0 Hz	-	“	“	“
129	from chambers					

Table S2: Complete parameter protocol for the manual reference workflow.

Step number	Step description	Pipetting	Vortex	Centrifuge	Thermal cycling	Magnet movement
1	Thaw DNA and reagents at suitable temperature				1	
2	Prepare Thermal Cycler					
3	Prepare 200 µL PCR tubes for samples					

4	Vortex DNA samples and centrifuge briefly (3 s)		1	1	
5	Add 5 μL DNA into each PCR tube	1			
6	Vortex Tagmentation Buffer and centrifuge briefly (3 s)		1	1	
7	Add 10 μL Tagmentation Buffer into each PCR tube	1			
8	Mixing	10			
9	Vortex Amplicon Tagmentation Mix and centrifuge briefly (3 s)		1	1	
10	Add 5 μL Amplicon Tagmentation Mix into each PCR tube	1			
11	Mixing	10			
12	Centrifuge briefly (3 s)			1	
13	Place PCR tubes into thermal cycler for tagmentation				1
14	Vortex Neutralize Tagmentation Buffer and centrifuge briefly (3 s)		1	1	
15	Remove PCR tubes				
16	Add 5 μL of Neutralize Tagmentation Buffer into each PCR tube	1			
17	Mixing	10			
18	Centrifuge briefly (10 s)			1	
19	Incubate at room temperature for 5 minutes				
20	Vortex Forward Primer and centrifuge briefly (3 s)		1	1	
21	Add 5 μL Forward Primer into each PCR tube	1			
22	Vortex Reverse Primer and centrifuge briefly (3 s)		1	1	

23	Add 5 μL Reverse Primer into each PCR tube	1				
24	Vortex PCR Master Mix and centrifuge briefly (3 s)		1	1		
25	Add 15 μL PCR Master Mix into each PCR tube	1				
26	Mixing	10				
27	Centrifuge briefly (30 s)			1		
28	Place PCR tubes in thermal cycler for amplification				1	
29	Remove PCR tubes					
30	Centrifuge briefly (20 s)					
31	Vortex AMPure Beads and centrifuge briefly (3 s)		1	1		
32	Add 90 μL AMPure Beads into each PCR tube	1				
33	Vortex for 10 s		1			
34	Incubate at room temperature for 5 minutes					
35	Place magnet stand on the PCR tubes, and wait for 2 minutes					1
36	Remove supernatant by pipetting liquid out	2				1
37	Vortex Washing Buffer (80% Ethanol) and centrifuge briefly (3 s)		1	1		
38	Add 200 μL Washing Buffer into each PCR tube	1				
39	Incubate for 30 s					
40	Remove supernatant by pipetting liquid out	2				
41	Add 200 μL Washing Buffer into each PCR tube	1				
42	Incubate for 30 s					

43	Remove supernatant by pipetting liquid out	2			
44	Incubate at room temperature for 15 minutes				
45	Remove tubes from the magnet stand				1
46	Vortex Elution Buffer and centrifuge briefly (3 s)		1	1	
47	Add 52.2 μL Elution Buffer into each PCR tube	1			
48	Mixing	10			
49	Incubate for 5 minutes				
50	Place the tubes in the magnet stand				1
51	Incubate for 2 minutes				
52	Transfer supernatant into new tubes	1			

Table S3: The reported index hopping rate with non-redundant dual index[83].

In this work, the Nextera XT DNA library preparation protocol, UD Indexes, and a high-throughput sequencer (NextSeq 1000/2000) were used. Therefore, the average rate of index hopping for this combination should be 0.68 % ± 0.19 %. The mapped read and coverage breadth percentages in negative controls were in the range of 0.1–0.5 %, lower than 0.68 %.

Library type	Method description	Multiplex PCR prior to sequencing?	Mean library yield	Index swap rate (%)[a]		
				MiSeq	HiSeqX or 4000	NovaSeq[b]
PCR-free genomes	DNA shearing + adapter ligation	-	2.8 nM	0.13 ± 0.08	3.01 ± 0.91	4.85 ± 0.88
PCR-plus genomes	DNA shearing + adapter ligation	-	141.1 nM	0.03 ± 0.04	0.24 ± 0.06	*no data*
Somatic exome	DNA shearing + adapter ligation	+	354.2 nM	0.67 ± 0.08	0.83 ± 0.23	0.52%
Germline exome	Nextera transposase	+	286.2 nM	0.49 ± 0.12	0.68 ± 0.19	*no data*
Stranded mRNA	cDNA prep + adapter ligation	-	39.8 nM	0.01 ± 0.001	0.32 ± 0.02	*no data*

[a]All swap rate values for each library type are means of 8 different pool & flowcell observations

[b]As of submission, NovaSeq data had only been generated for PCR-free genomes and one flow cell of exomes

Table S4: The mutation detection concordance between the reference genome and the results in the automated and reference manual workflows.

Species	Coll lineage	Sub-lineage	Manual	Automated
M. tuberculosis	H37Rv-like	-	3/3	3/3
M. bovis	BOV	-	3/3	3/3
M. tuberculosis	Beijing (2.2.1)	Central Asia Outbreak	3/3	3/3

M. tuberculosis	Beijing (2.2.1)	Europe/Russia W148 Outbreak	3/3	3/3

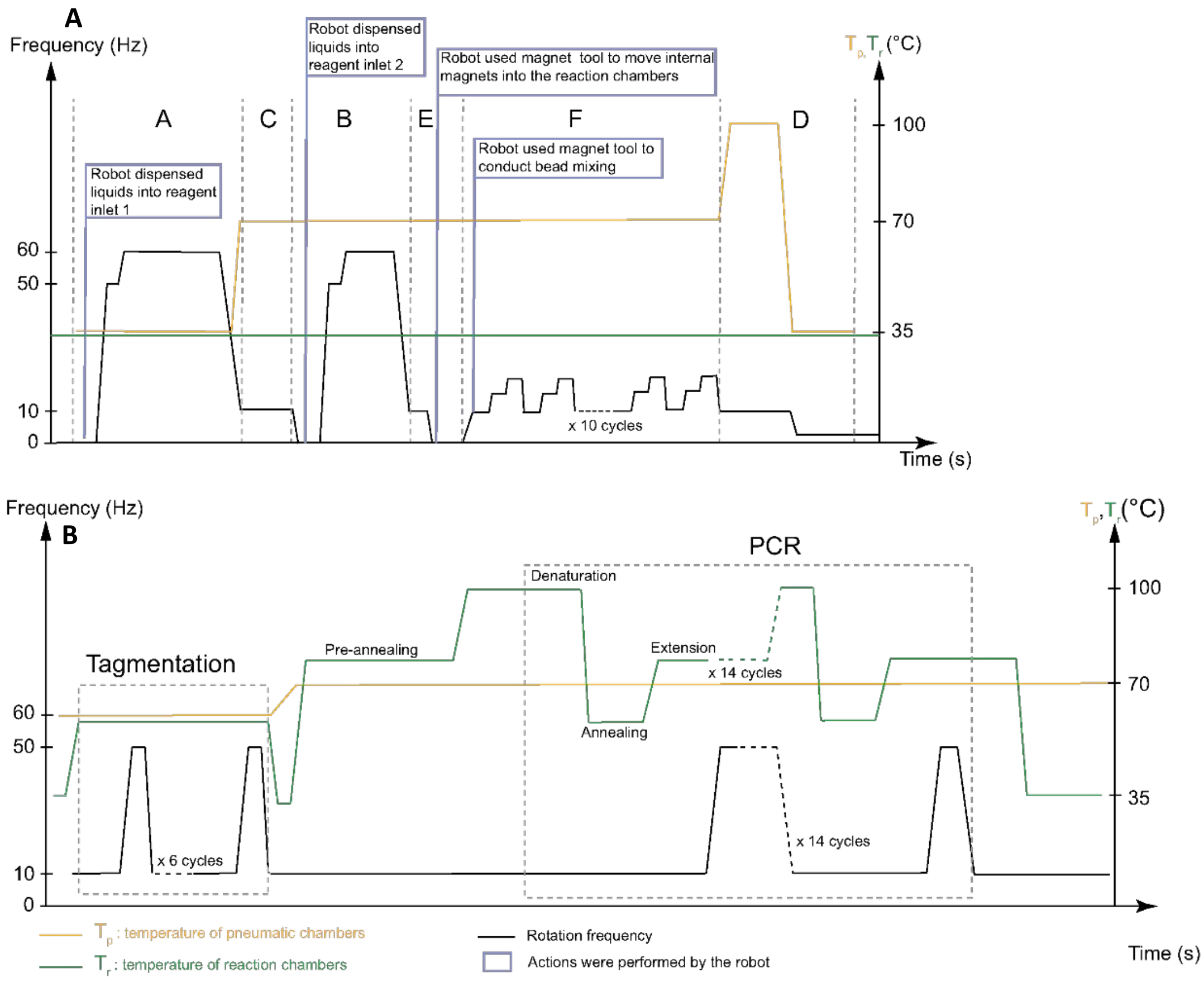

Figure S1: Illustration of automation protocol for the rotation frequency, the temperature of the pneumatic chambers (T_p), and the temperature of the reaction chambers (T_r). Figure 1.A presents the automation protocol for six unit operations (A, B, C, D, E, and F). The protocols for unit operations A and B are similar: the rotor first stops rotating, and the robot dispenses liquids into the reagent inlets of the cartridge. The rotor then increases its frequency from 0 to 50 Hz to aliquot liquids into the aliquoting chambers, and to 60 Hz to transfer liquids. The protocol for unit operation C is simple, in which the rotor keeps rotating at 10 Hz while heating the pneumatic chambers for bubble mixing. In unit operation E, the rotor stops spinning so that the robot can move the internal magnets from the pre-stored location to the bottom of the reaction chambers. In unit operation F, the rotor increases the frequency from 10 to 20 Hz in 10 cycles while the robot positions the magnet tool on top of the cartridge to enable bead mixing. In unit operation D, heating the pneumatic chambers to 100 ˚C followed by cooling them to 35 ˚C enables liquid removal. Figure 1.B illustrates the automation protocol for tagmentation and amplification. Before tagmentation, heating the pneumatic chambers from room temperature to 60 ˚C mixes liquids (unit operation C). The bottom graph presents the automation protocol for the tagmentation and amplification. During tagmentation, the rotor spins mainly at 10 Hz, and then quickly increases to 50 Hz to bring all liquid residues inside the chambers down. In addition, heating the reaction chambers to 58 ˚C for 5 min triggers

tagmentation. Before amplification, another liquid mixing occurs by increasing the temperature of the pneumatic chambers from 60 ˚C to 70 ˚C. During amplification, the temperature of the reaction chambers follows 14 thermal cycles, including denaturation, annealing, and extension phases. The rotation frequency during amplification is similar to that during tagmentation.

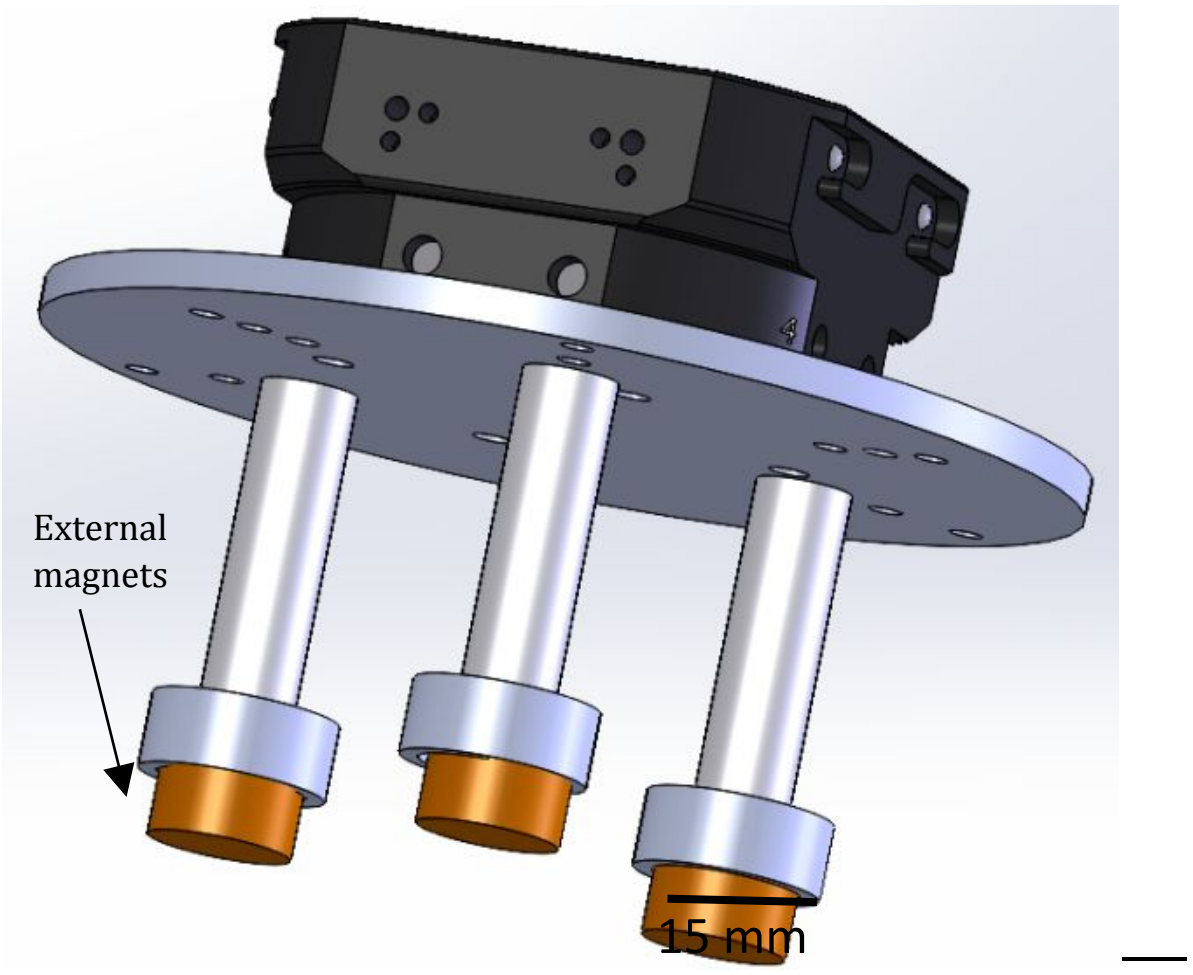

Figure S2: The magnet tool used in this study, with three external magnets (shown in orange), to move internal magnets and mix beads.

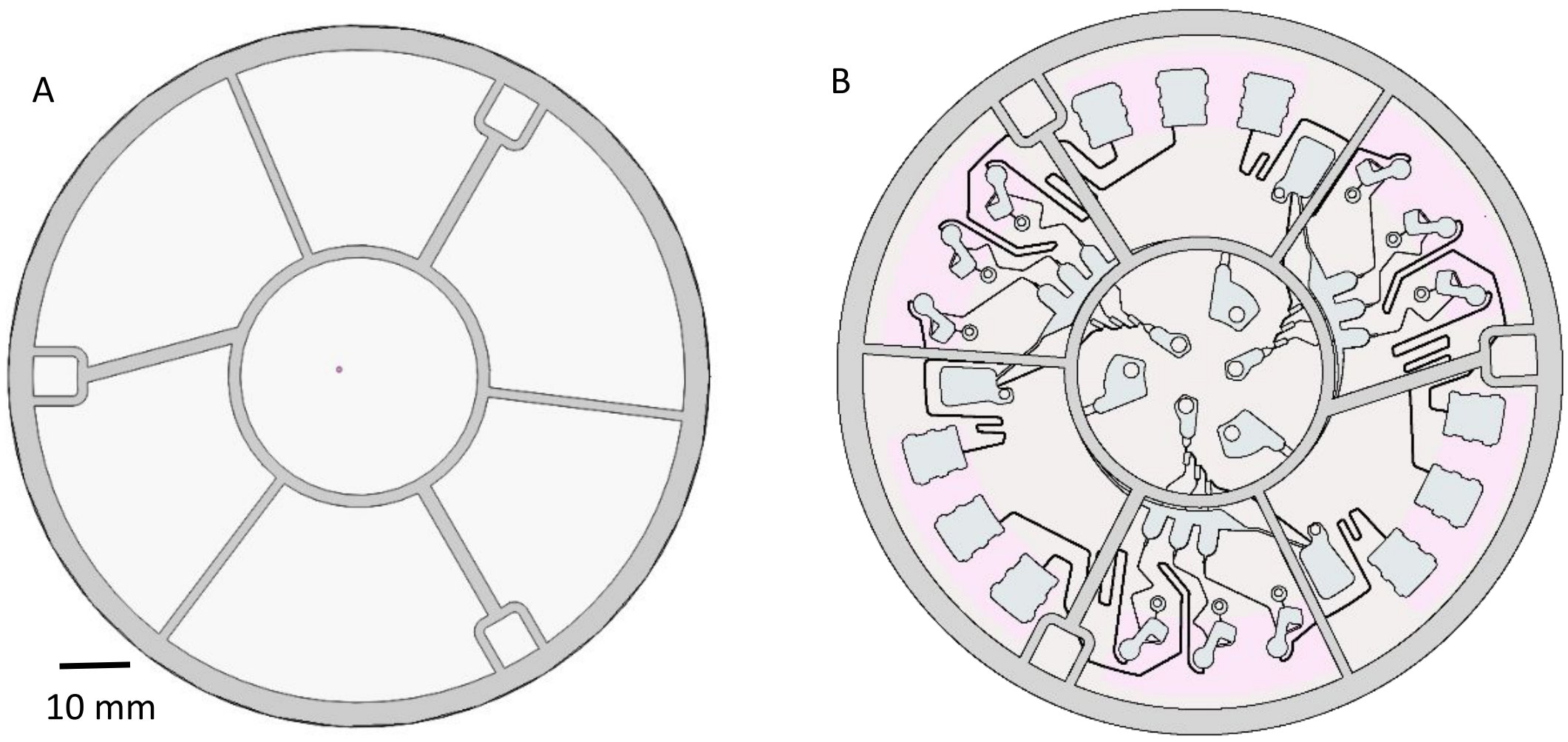

Figure S3: A presents the CAD design of a thermal insulation cover. B presents the complete cartridge with the thermal insulation cover on top. The cover isolates the chambers from the environment during heating and rotation. In addition, the cover divides the cartridge into six separate areas to minimize the thermal exchange between them.

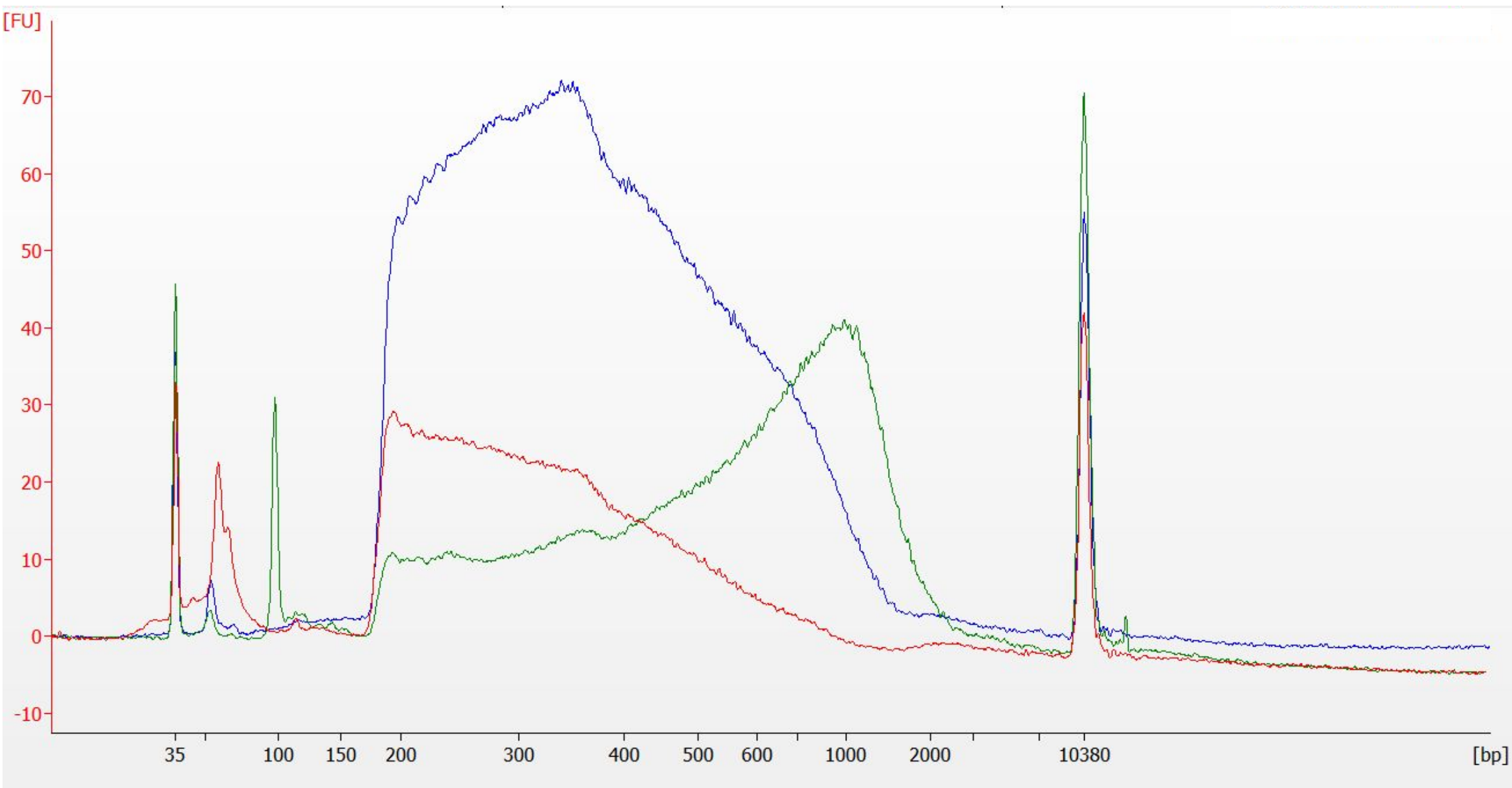

Figure S4: The DNA fragment size distribution in the automated tests for MTBC strain S3. The color of each line represents one reaction chamber: blue for Reaction Chamber 1, green for Reaction Chamber 2, and red for Reaction Chamber 3.

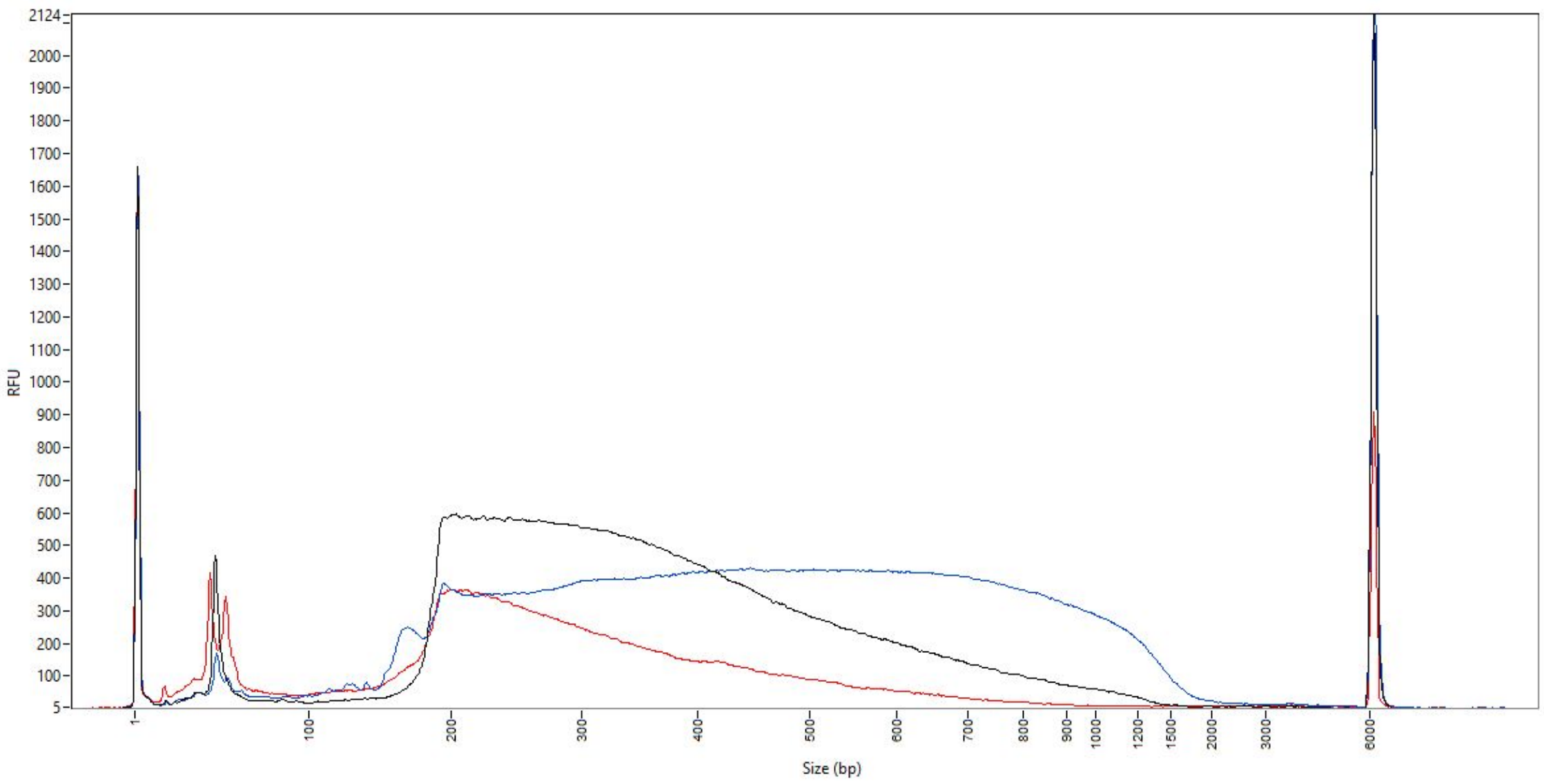

Figure S5: The DNA fragment size distribution in the automated tests for MTBC strain S4. The color of each line represents one reaction chamber: blue for Reaction Chamber 1, black for Reaction Chamber 2, and red for Reaction Chamber 3.

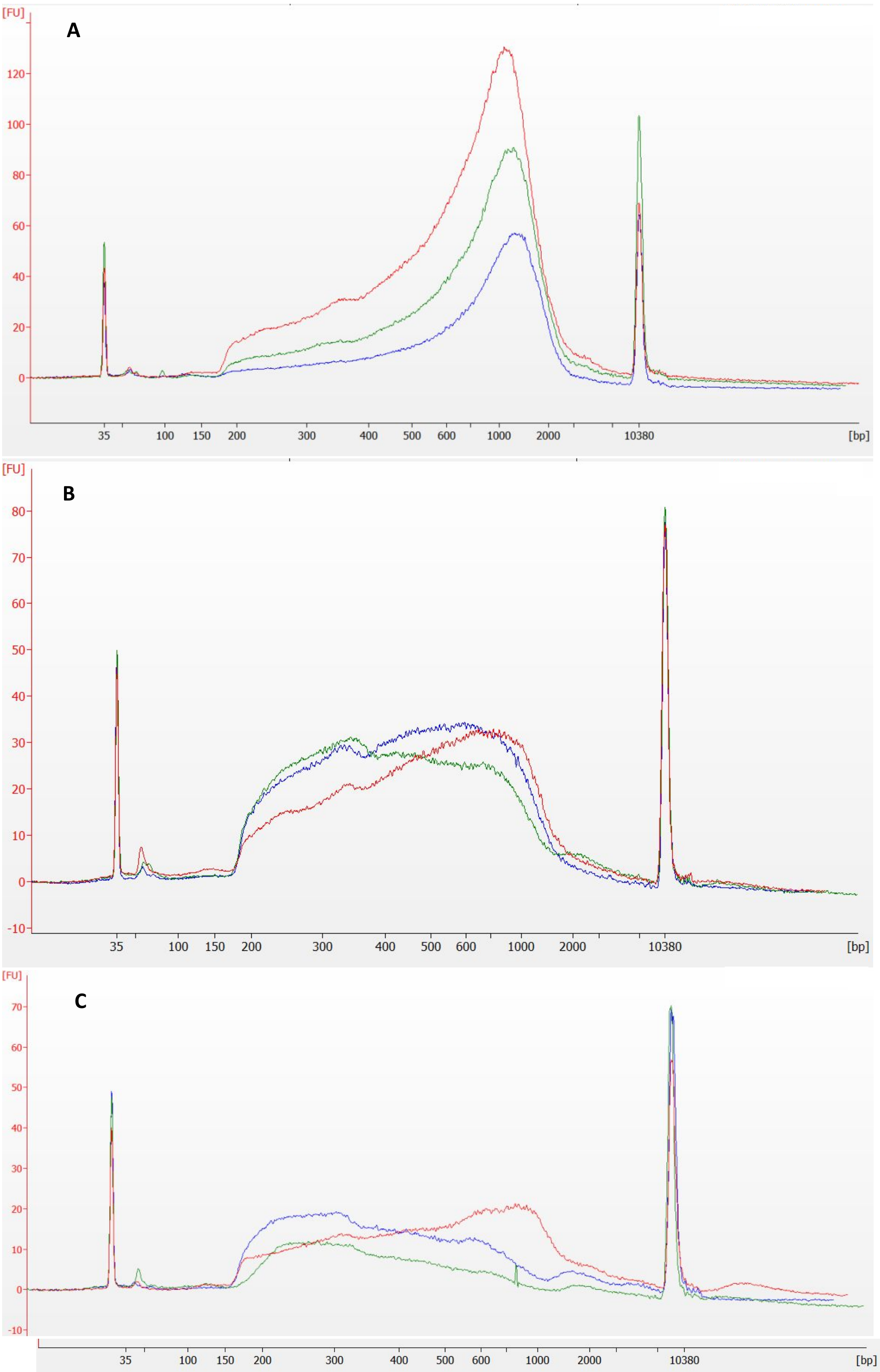

Figure S6: The DNA fragment size distribution in the reproducibility tests of reaction chambers 1–3 across three different slices. A, B, and C present slice 1, slice 2, and slice 3, respectively. In each slice, the color of each line represents one reaction chamber: blue for Reaction Chamber 1, green for Reaction Chamber 2, and red for Reaction Chamber 3.

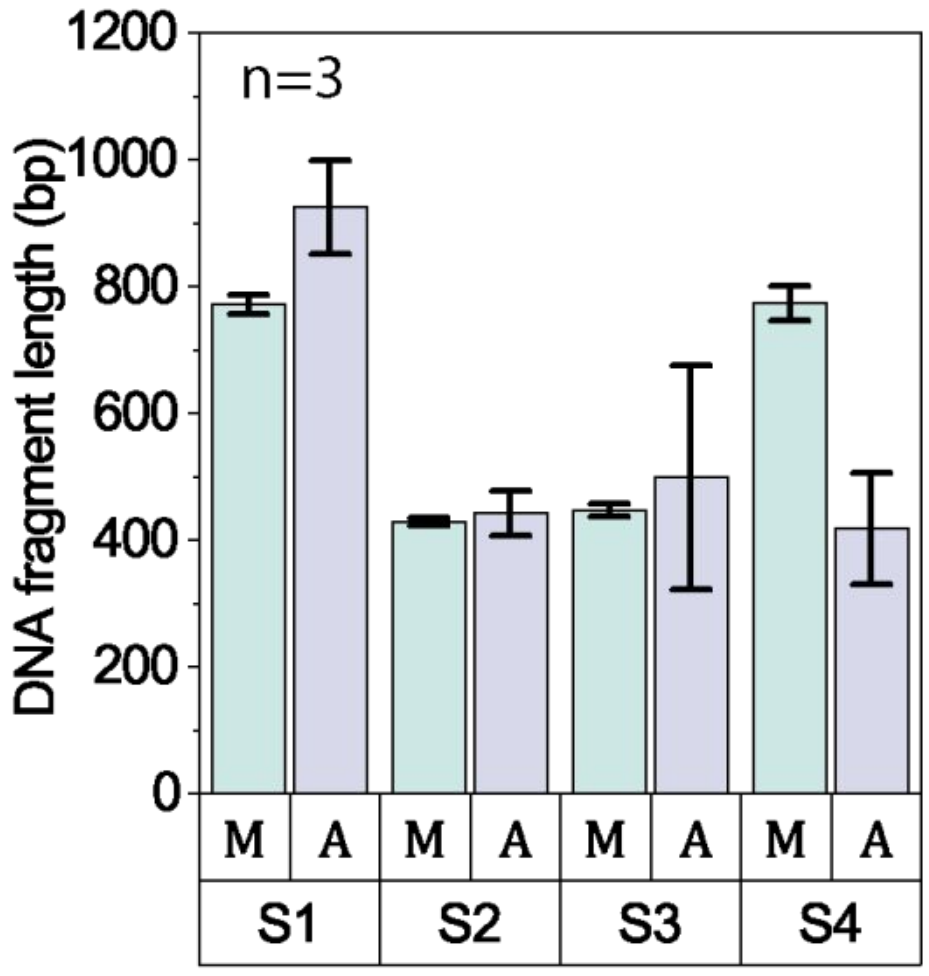

Figure S7: The average DNA fragment size between manual (M) and automated (A) workflows among four different MTBC strains (S1, S2, S3, and S4). The fragment sizes in manual vs. automated were: 771 bp (CV ± 1.91 %) *vs.* 924 bp (CV ± 8 %) for S1; 428 bp (CV ± 1.42 %) *vs.* 442 bp (CV ± 7.9 %) for S2; 446 bp (CV ± 2.3 %) *vs.* 498 bp (CV ± 35.7 %) for S3; and 773 bp (CV ± 3.51 %) *vs.* 417 bp (CV ± 21.9 %) for S4.

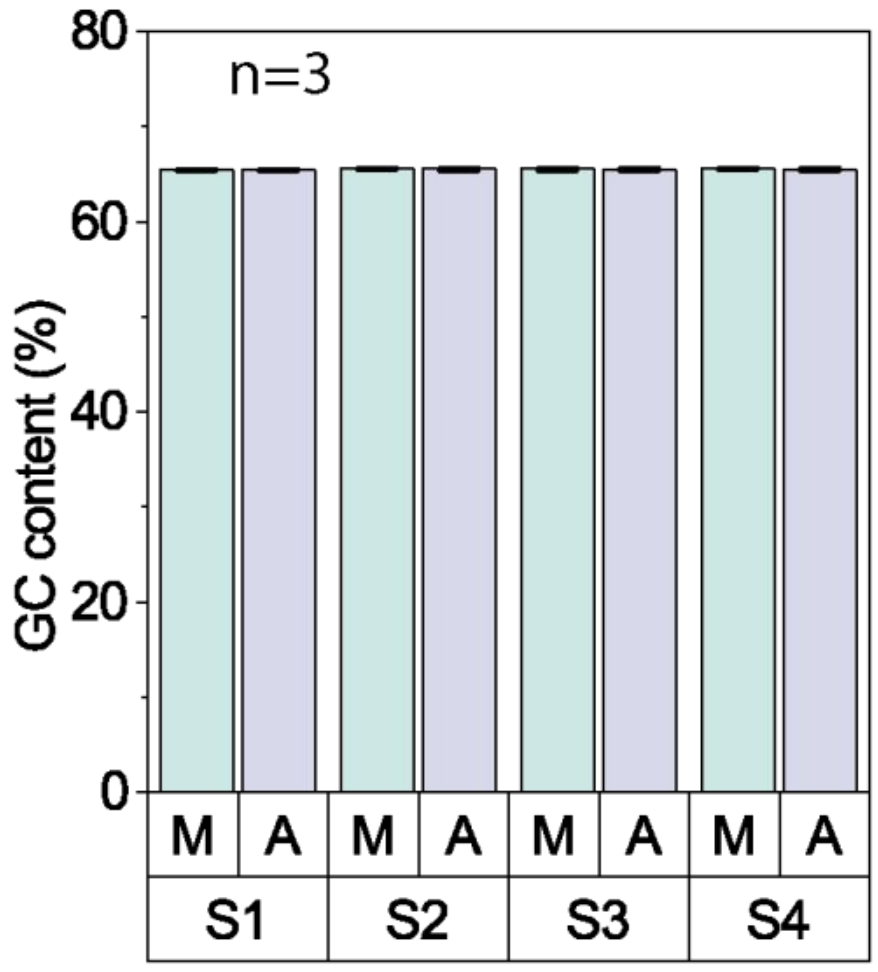

Figure S8: The average GC content (%) of total reads between manual (M) and automated (A) workflows among four different MTBC strains (S1, S2, S3, and S4). The GC content percentages in manual vs. automated were comparable across different strains.

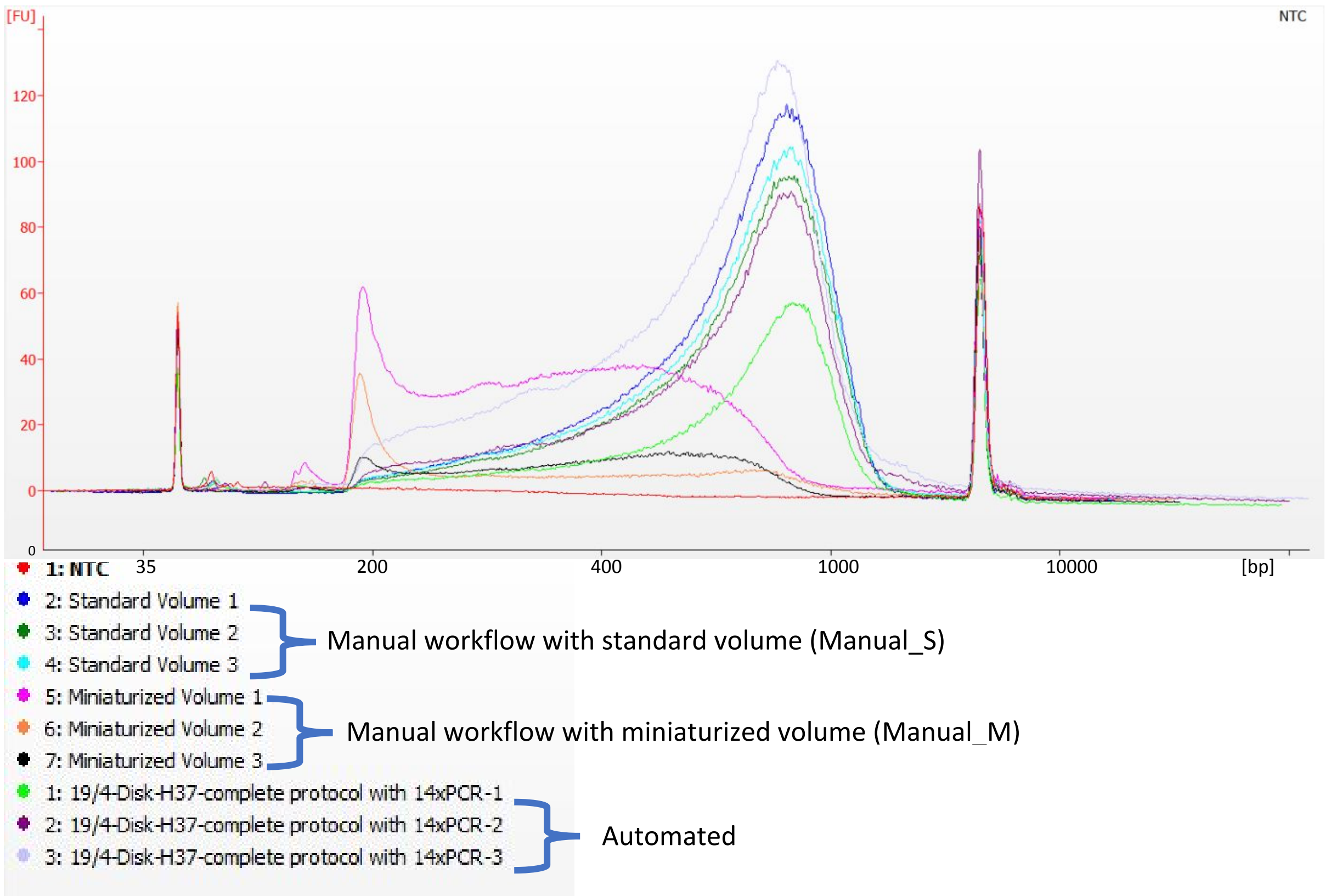

Figure S9: Comparison of DNA fragment length distributions among the manual workflow with standard volume (Manual_S), the manual workflow with miniaturized volume (Manual_M), and the automated workflow with miniaturized volume (Automated) for S1 strain.

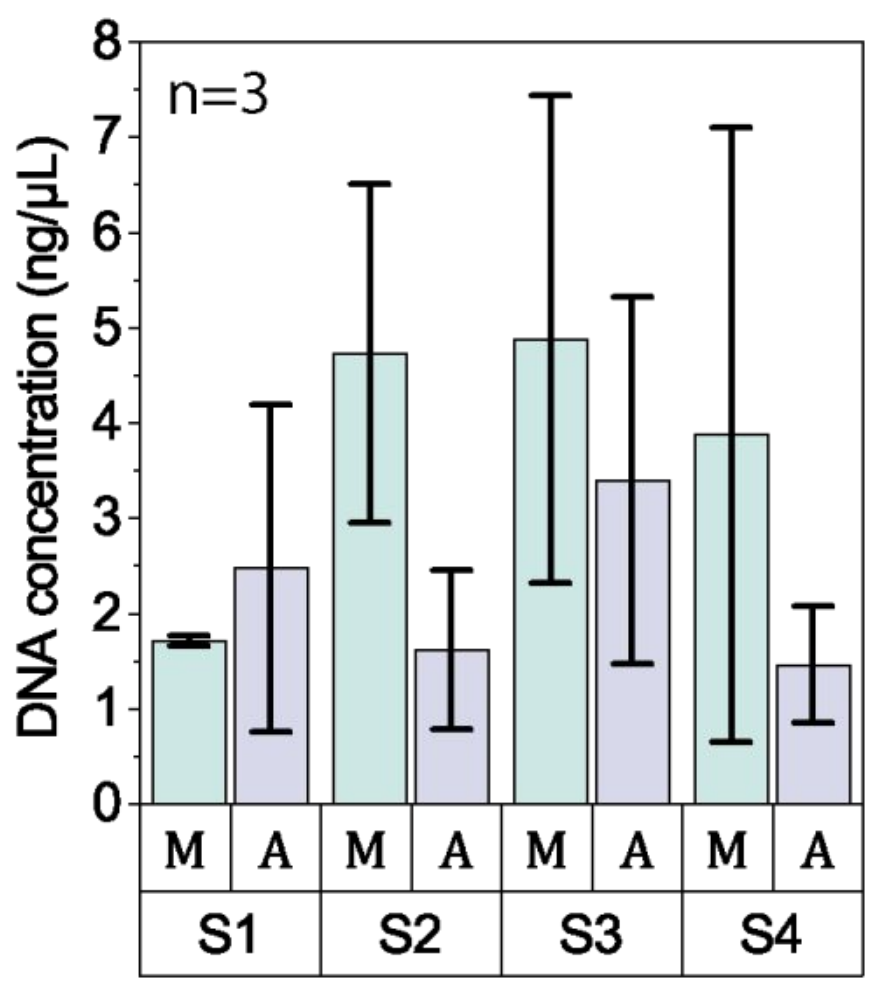

Figure S10: DNA yield post library preparation and before normalization for sequencing in the automated workflow (A) and the manual workflow (M) for four MTBC strains (S1, S2, S3, and S4). Yields varied substantially in both workflows, which complicated direct comparison. For S1, the automated workflow gave a higher average yield than the manual workflow (2.47 vs. 1.70 ng/µL, a factor of 1.45). Two additional amplification cycles correspond to a fourfold increase in product; because the automated workflow started from half the DNA input (0.5 vs. 1 ng), the expected yield ratio was approximately twofold rather than fourfold, in reasonable

agreement with the measured value. For S2–S4, average yields (automated vs. manual) were 1.62 vs. 4.72 ng/μL (S2), 3.39 vs. 4.48 ng/μL (S3), and 1.46 vs. 3.87 ng/μL (S4). Here the automated input was 0.05 ng against 1 ng, a 20-fold reduction; offset by the fourfold gain from two extra cycles, the manual workflow would be expected to yield approximately fivefold more. The measured manual-to-automated ratios were all below this value (2.9× for S2, 1.32× for S3, 2.65× for S4), indicating that the additional cycles compensated for the reduced input more effectively than predicted. Given the yield variance in both workflows, no conclusion is drawn as to which yields more DNA; the relevant outcome is that 14 cycles reliably provided sufficient library DNA for normalization prior to sequencing.

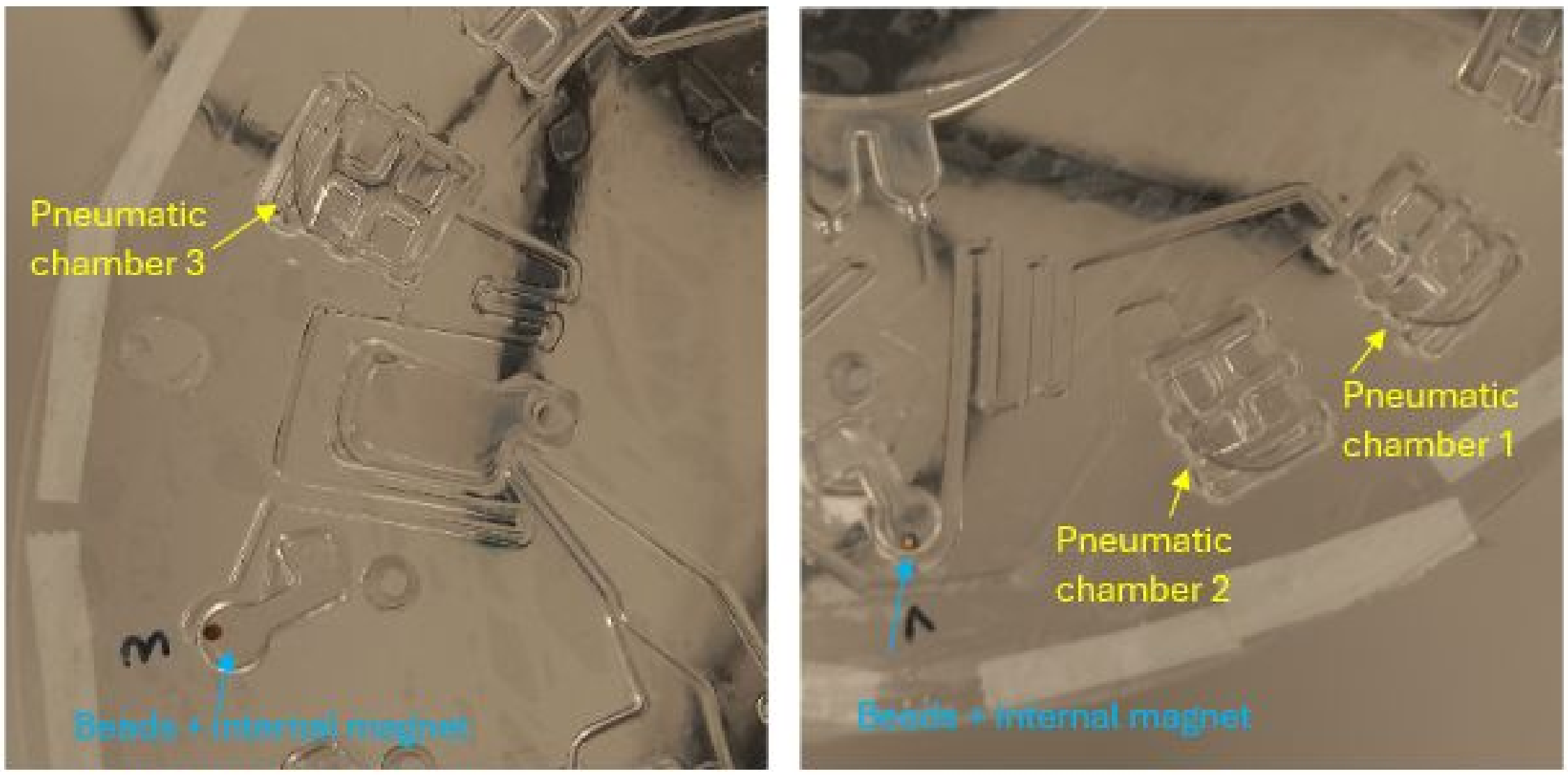

Figure S11: Magnetic bead retention in the reaction chambers. Images captured after the binding and washing steps show no visible bead loss; the beads remain within the chambers, retained by the internal magnets.

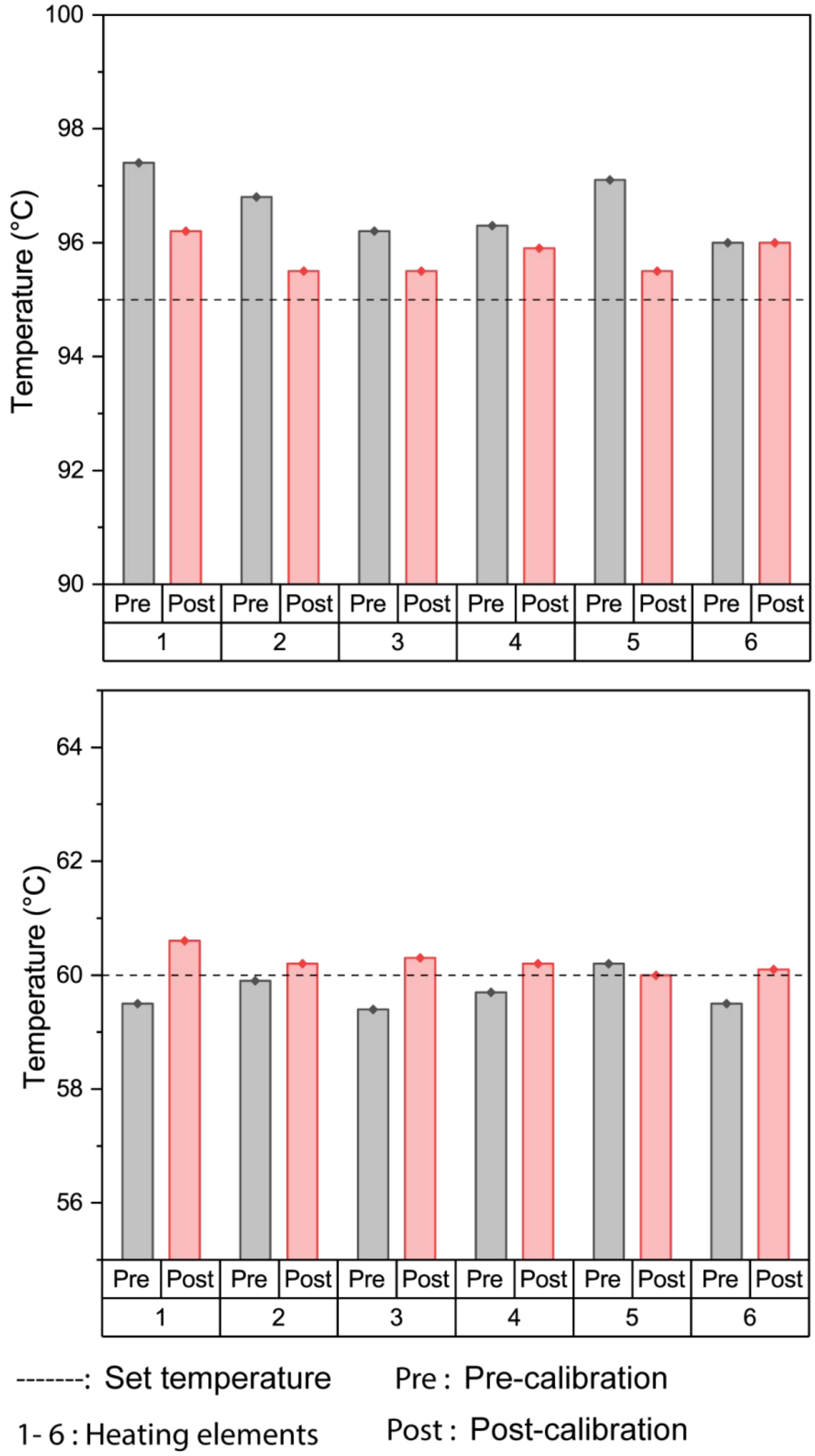

Figure S12: Pre- and post-calibration temperature data from six heating elements. The temperature calibration was performed using an infrared thermal camera to measure the temperature of each heating zone at two set points, 60 ˚C and 95 ˚C. The graphs show pre- and

post-calibration measurements for the six heating elements at both set points. After calibration, the measured temperatures deviated from the set points by 0.6 ˚C at 60 ˚C and 1.2 ˚C at 95 ˚C.

ESI Cost calculation

The cartridge cost is divided into two main categories: the cartridge manufacturing cost and the reagent cost per sample.

- Total cartridge cost = Total manufacturing cost + N × Total reagent cost per sample, where N is the number of samples per cartridge.
- Total manufacturing cost = Tooling cost + COC material cost + Molding cost + Thermal sealing cost + Quality control cost.
- Total reagent cost per sample = Original reagent cost per sample × miniaturization level.

Table S5: Total reagent cost per sample and total cartridge cost with detailed calculation.

Production volume [units]	Tools required	Total Tool Capex [$]	Tooling Cost [$/unit]	COC Material Cost [$/cartridge]	Moulding Cost [$/cartridge]	Thermal Sealing Cost [$/cartridge]	Quality Control Cost [$/cartridge]	Total Manufacturing Cost [$/cartridge]	Total Reagent Cost per sample [$/sample]	Total Cartridge Cost [$/cartridge]
1000	1	7000	7,00	0,23	2,53	2,53	1,64	13,94141667	5	58,94141667
10000	1	7000	0,70	0,23	2,53	2,53	1,64	7,641416667	5	52,64141667
100000	1	70000	0,70	0,23	2,53	2,53	1,64	7,641416667	5	52,64141667
1000000	1	70000	0,07	0,23	2,53	2,53	1,64	7,011416667	5	52,01141667
10000000	9	630000	0,06	0,23	2,53	2,53	1,64	7,004416667	5	52,00441667

The table below summarizes the total manufacturing cost, total reagent cost per sample, and total cartridge cost across different production volumes. Once the production volume exceeds 10,000 cartridges, the manufacturing cost falls below $10 per cartridge. Thanks to centrifugal microfluidics, the reagent cost falls to $5 per sample. For a cartridge processing 9 samples, the total cartridge cost is therefore under $60. This is highly competitive with current state-of-the-art systems, whose cartridge costs range from $300 to $1,000 for the Fluidigm Juno (MOgene), $400 for MagicPrep NGS (Tecan), and $400 to $500 for the BioQule NGS system (Revvity).

Tooling cost: *The total cost of the aluminum mold divided by the total number of cartridges produced with this mold.*

Table S6: An example of calculating tooling cost per cartridge

	Unit	Example
Aluminum Mold Cost	$	7000
Production volume	Number of cartridges	10000
Tooling cost	$/cartridge	0.7

Assumption:

- Micro-featured aluminum molds used for rapid injection molding cover 500–10,000 parts at \$2,000–\$5,000; we therefore assumed a price of \$7,000 for fewer than 10,000 parts [a]
- The estimated price of a molding tool for high-volume injection molding (up to 100,000 parts) is \$70,000 [a]

Cyclic olefin copolymer (COC) material cost: *The COC material cost per cartridge (molded part + sealing foil).*

Table S7: An example of calculating material cost per cartridge

	Unit	Example
Material cost for molded part	\$/cartridge	1.55
Material cost for sealing foil	\$/cartridge	0.2
COC Material Cost	\$/cartridge	1.75

Assumption:

- The price of the COC material is \$25/kg [b]
- The molded part of the COC cartridge has a diameter of 140 mm and a thickness of 4 mm (62 g).
- The sealing foil of the cartridge has a diameter of 140 mm and a thickness of 0.6 mm (8.68 g).

Molding cost: *The labor and operating cost of the molding press required to produce one cartridge.*

Table S8: An example of calculating tooling cost per cartridge

	Unit	Example
Labor Cost	\$/hr	26
Molding press operation cost	\$/hr	50
Cycle time	s/cartridge	120
Molding cost	\$/cartridge	2.53

Assumption:

- Labor cost: The operators are assumed to be in the mid pay range TV-L 7 (4,200 USD/month) [c], equivalent to $26/hour, assuming 20 working days per month and 8 hours per working day.
- Molding press cost: For a molding press with a clamping force below 22 T, one service provider quoted approximately $50/hour [d]
- Cycle time: Pressing one molded part of the cartridge takes approximately 120 seconds [e]

Thermal sealing cost: *The labor and operating cost of the thermal sealing machine required to produce one cartridge.*

Table S9: An example of calculating labor and operating cost per cartridge

	Unit	Example
Labor Cost	$/hr	26
Thermal sealing operation cost	$/hr	50
Cycle time	s/cartridge	120
Thermal sealing cost	$/cartridge	2.53

Assumption:

- Labor cost: The same assumption as for the molding cost calculation is used.
- Thermal sealing operation cost: The same cost as for the molding press operation is used here. This assumption is reasonable, as the thermal sealing machine typically requires less power (< 1 T) than the molding press.
- Cycle time: Sealing the foil onto the molded part takes approximately 120 seconds [f]

Quality control cost: *The cost of quality control required to ensure that cartridge production meets the relevant quality standards.*

Table S10: An example of calculating quality control cost per cartridge

	Unit	Example
Cartridge manufacturing cost	$/cartridge	7.6
Projected selling price for cartridge without reagent	$/cartridge	20
Quality Control Cost	$/cartridge	1.64

Assumption:

- Quality control cost for cartridge production: 6.8–9.4 % of total sales (the selling price of a cartridge without reagent is $20) [g]

Total reagent cost per sample: The original reagent cost per sample (as purchased from Illumina) divided by the miniaturization level.

Table S11: An example of calculating reagent cost per sample

	Unit	Example
Original reagent cost per sample	$/sample	50
Miniaturization level	x	1/10
Total reagent cost per sample	$/sample	5

Assumption:

- The Illumina Nextera XT DNA Library Preparation Kit used in this study costs $1,200 per 24 samples without miniaturization of reagent consumption [h].
- The miniaturization level in this study is 1/10.

Whole Genome Coverage for four MTBC strains (S1, S2, S3, and S4)

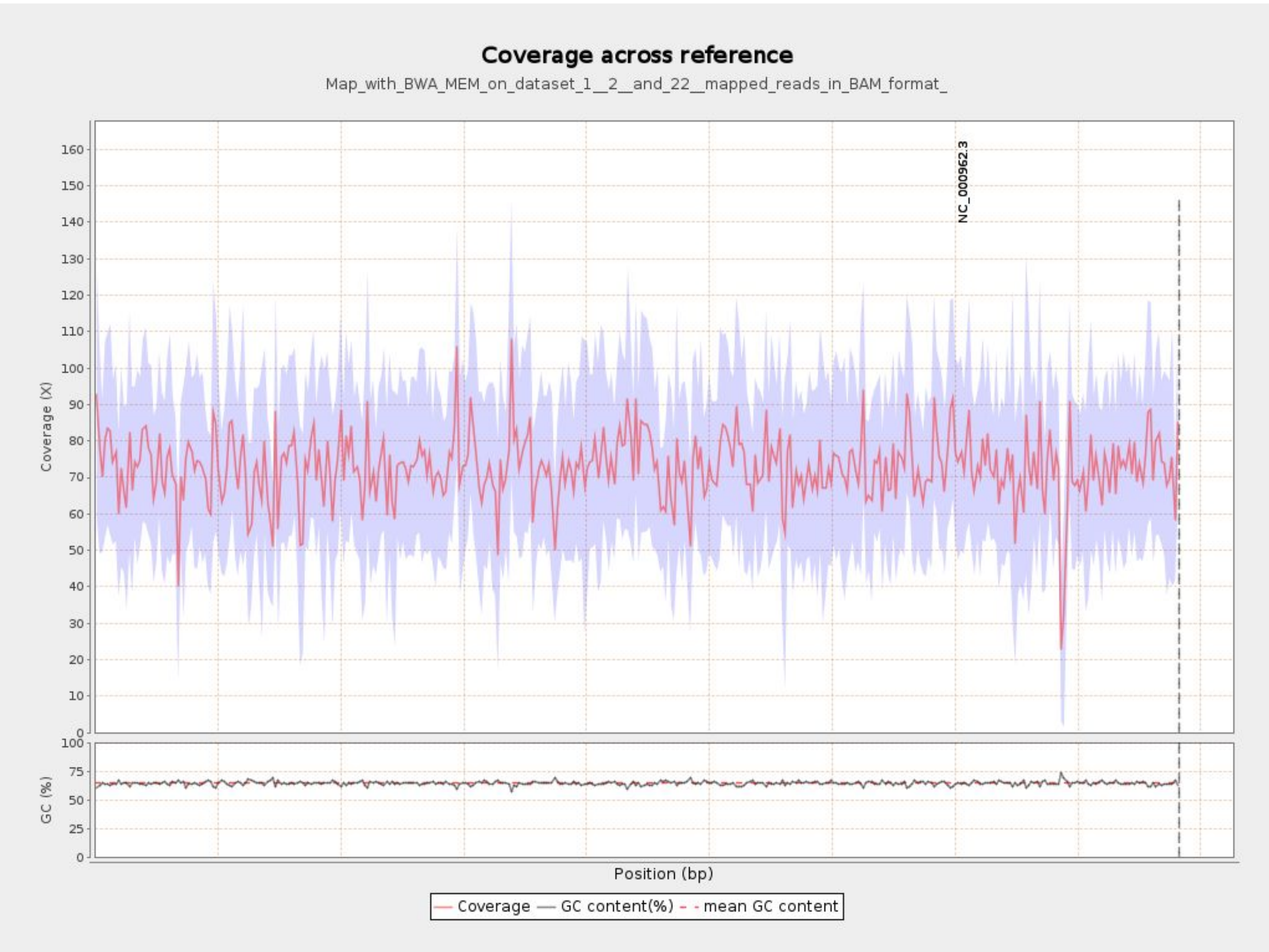

Figure S13: Whole genome coverage for 1st replicate in the manual test for strain S1

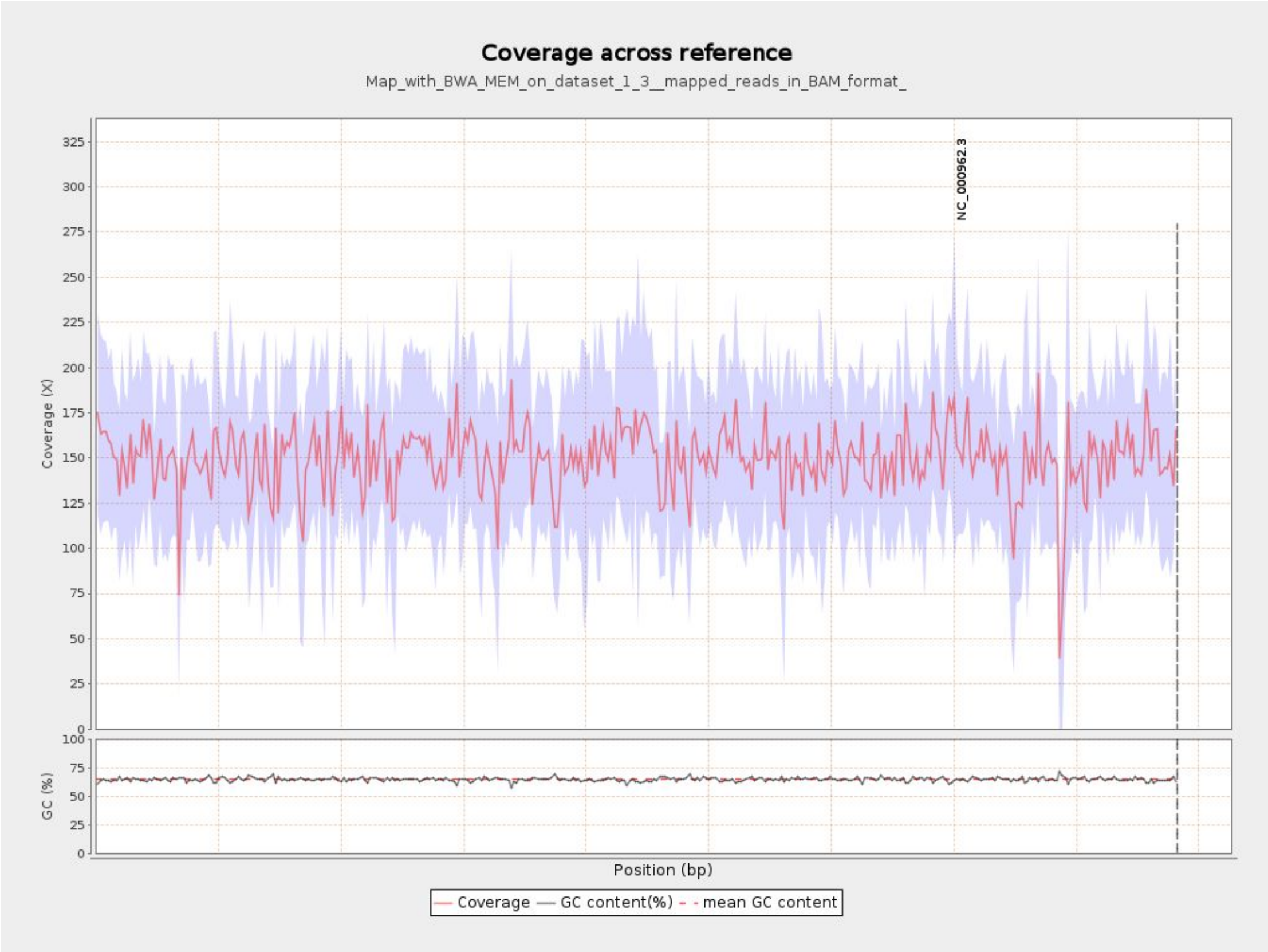

Figure S14: Whole genome coverage for 1st replicate in the automated test for strain S1

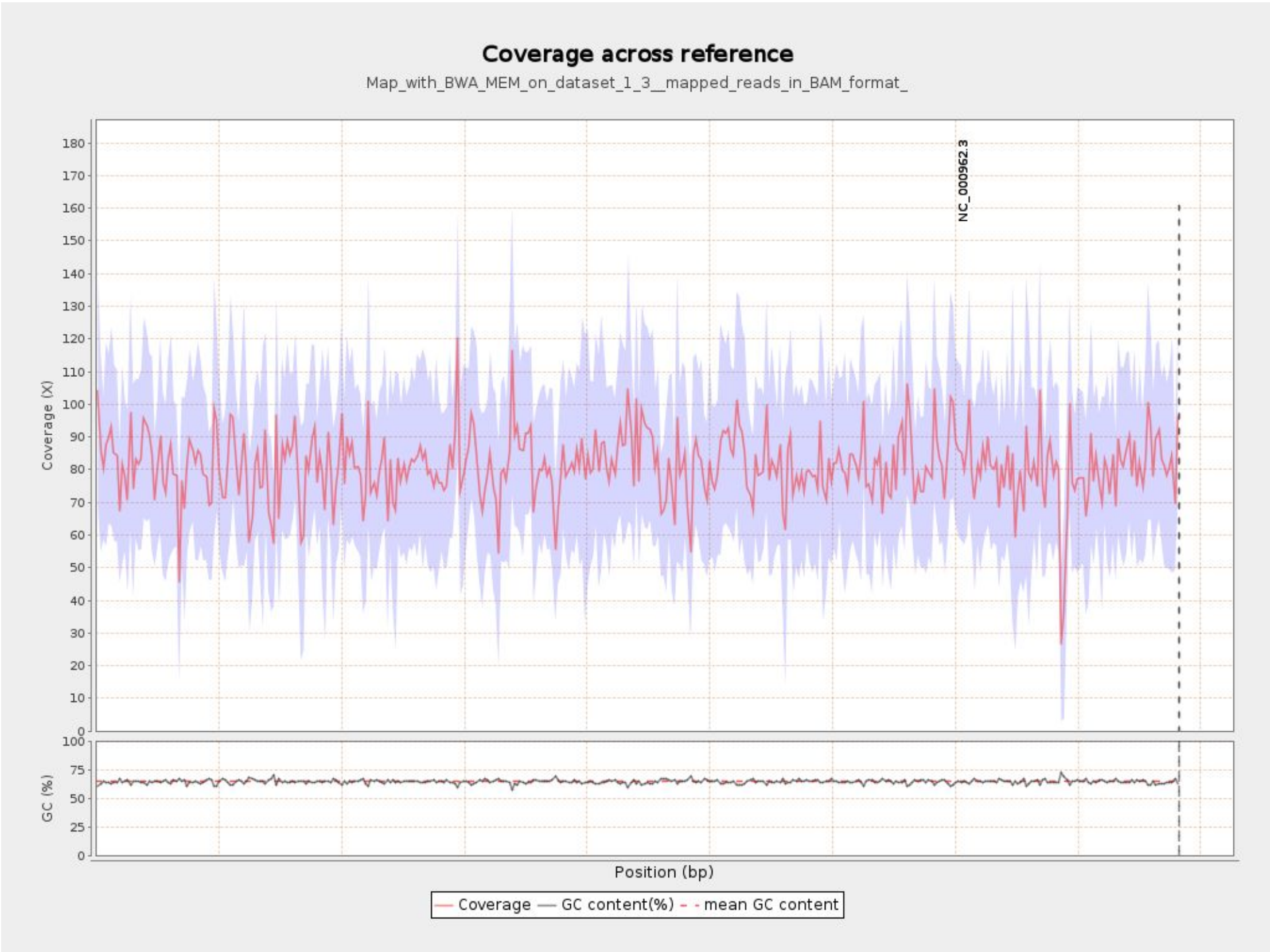

Figure S15: Whole genome coverage for 2nd replicate in the manual test for strain S1

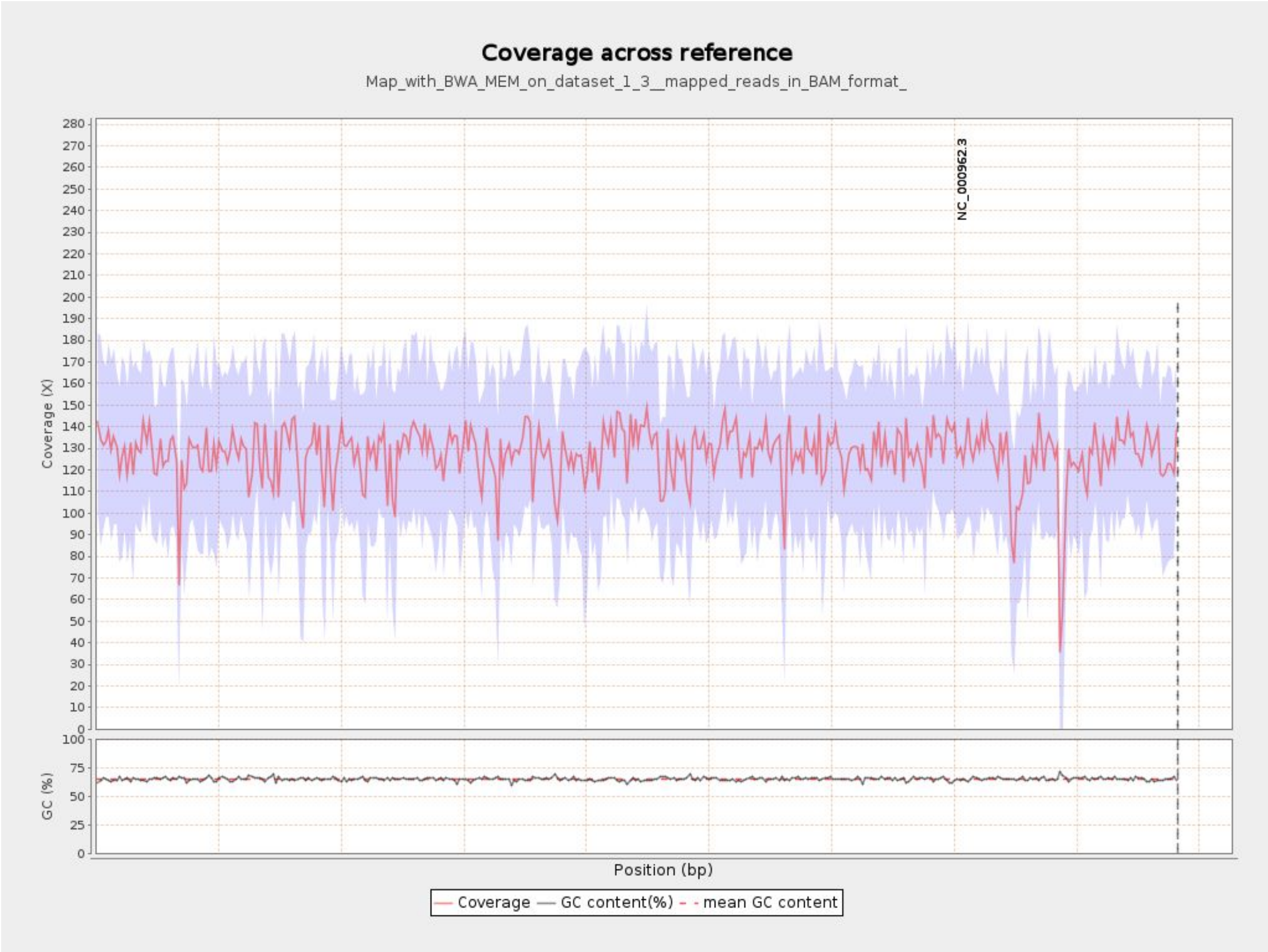

Figure S16: Whole genome coverage for 2nd replicate in the automated test for strain S1

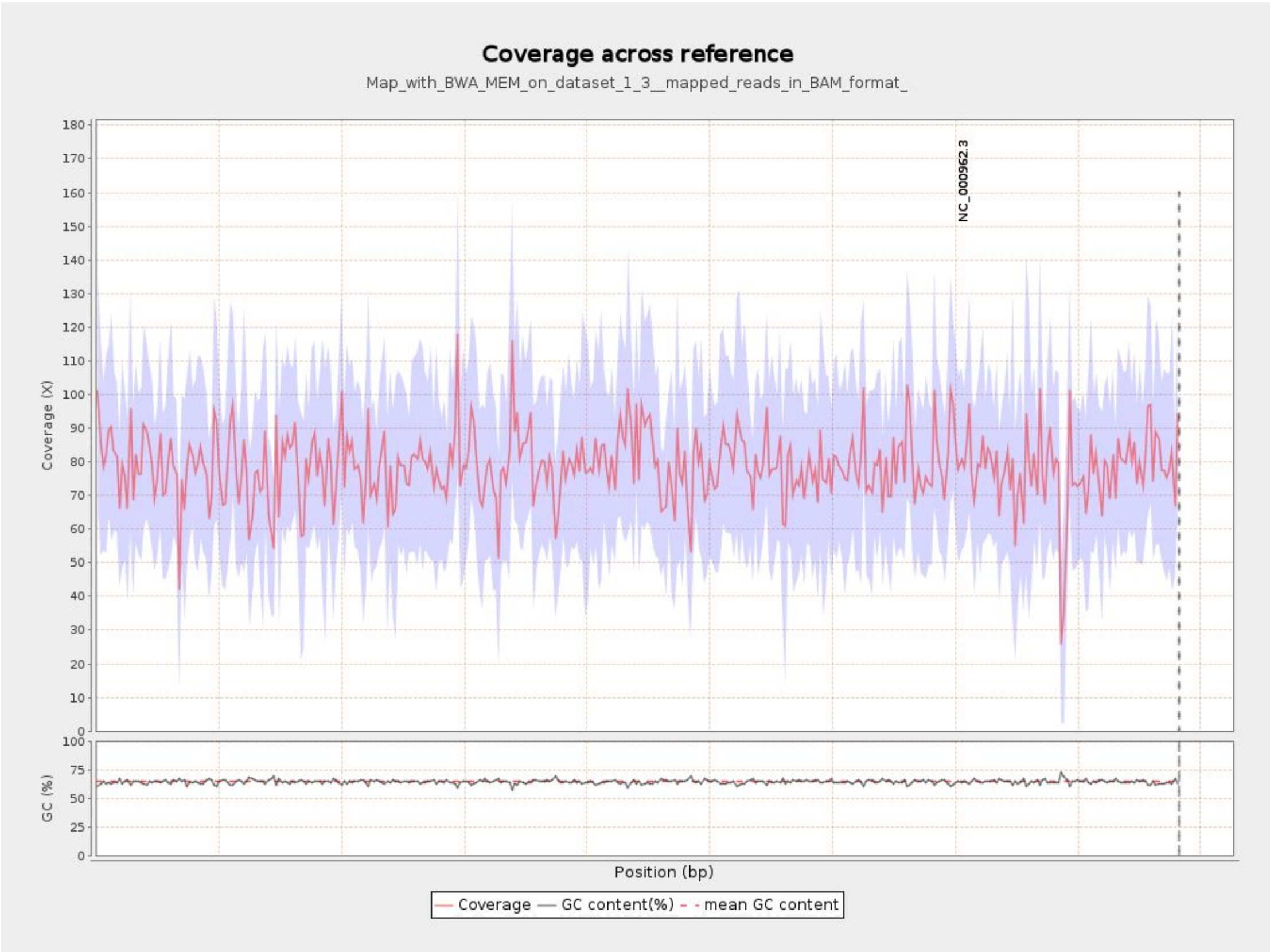

Figure S17: Whole genome coverage for 3rd replicate in the manual test for strain S1

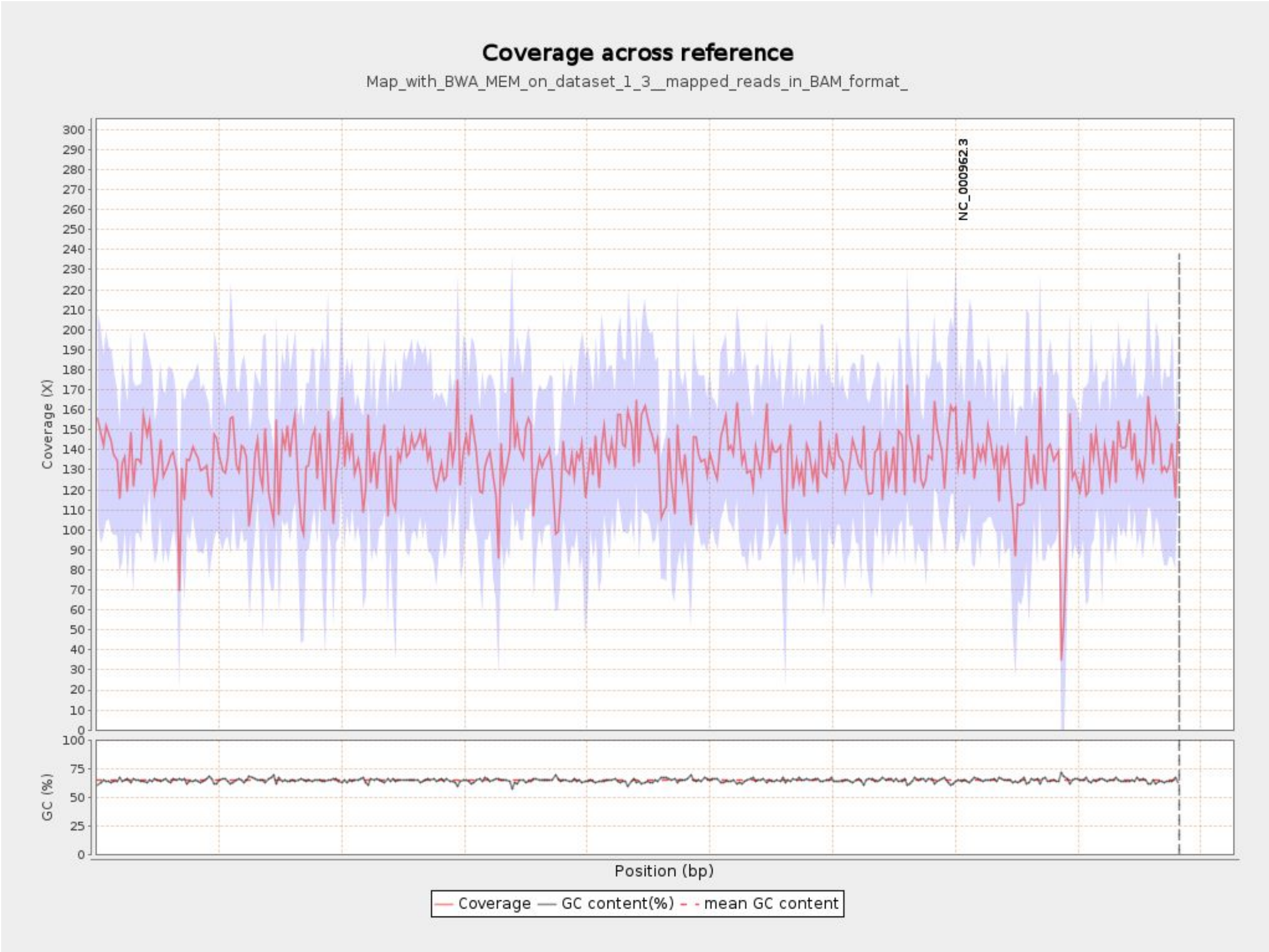

Figure S18: Whole genome coverage for 3rd replicate in the automated test for strain S1

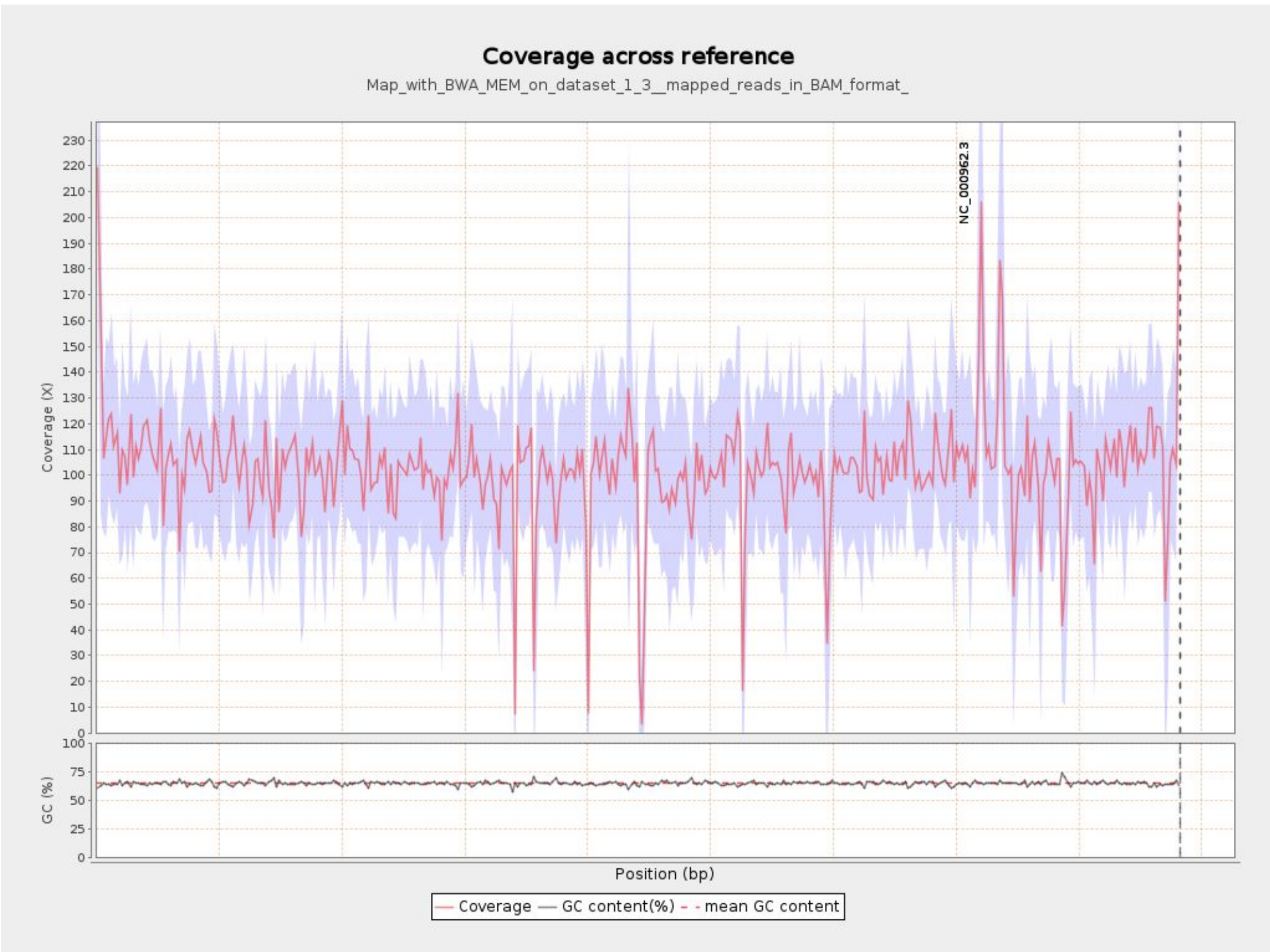

Figure S19: Whole genome coverage for 1st replicate in the manual test for strain S2

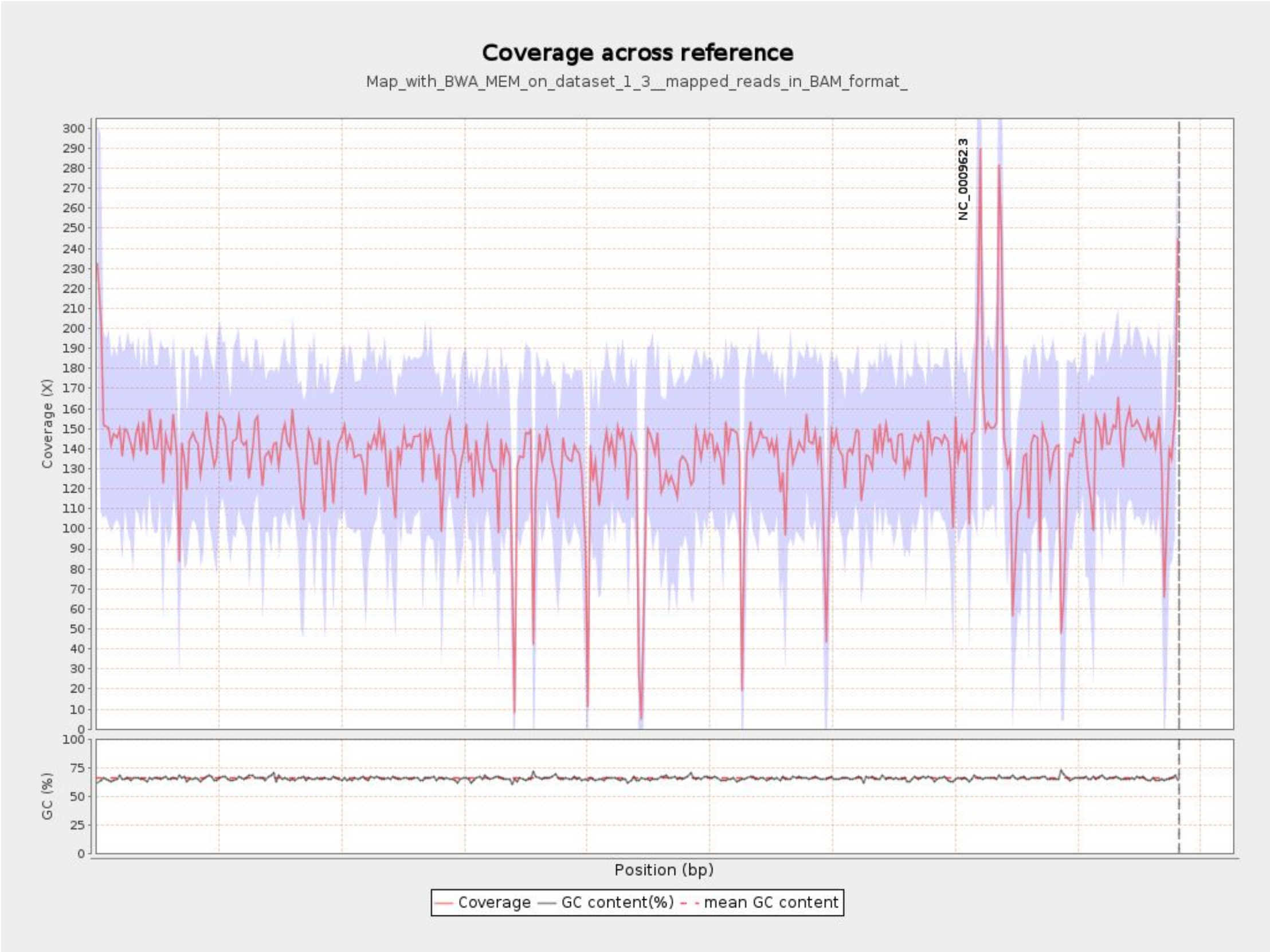

Figure S20: Whole genome coverage for 1st replicate in the automated test for strain S2

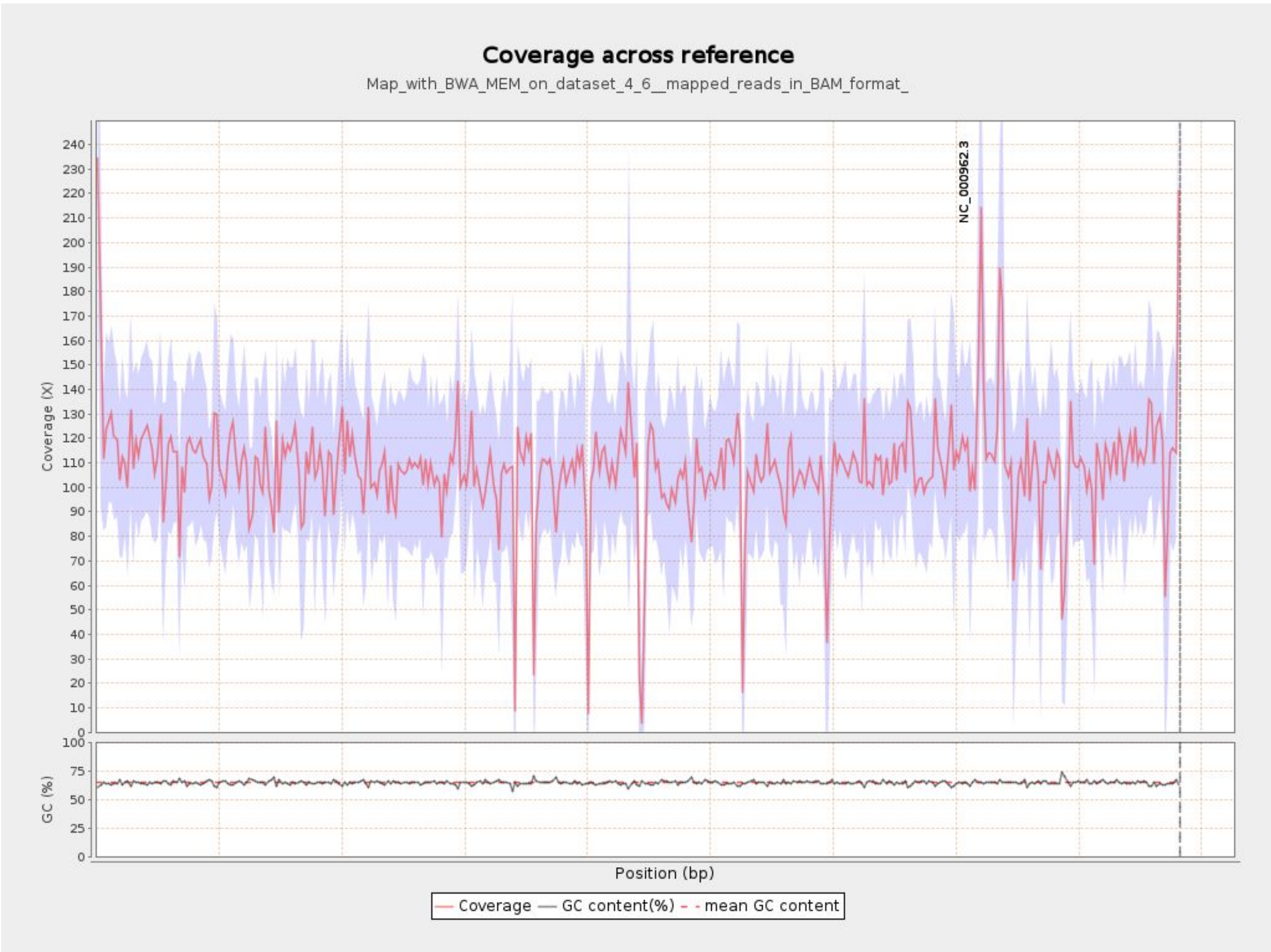

Figure S21: Whole genome coverage for 2nd replicate in the manual test for strain S2

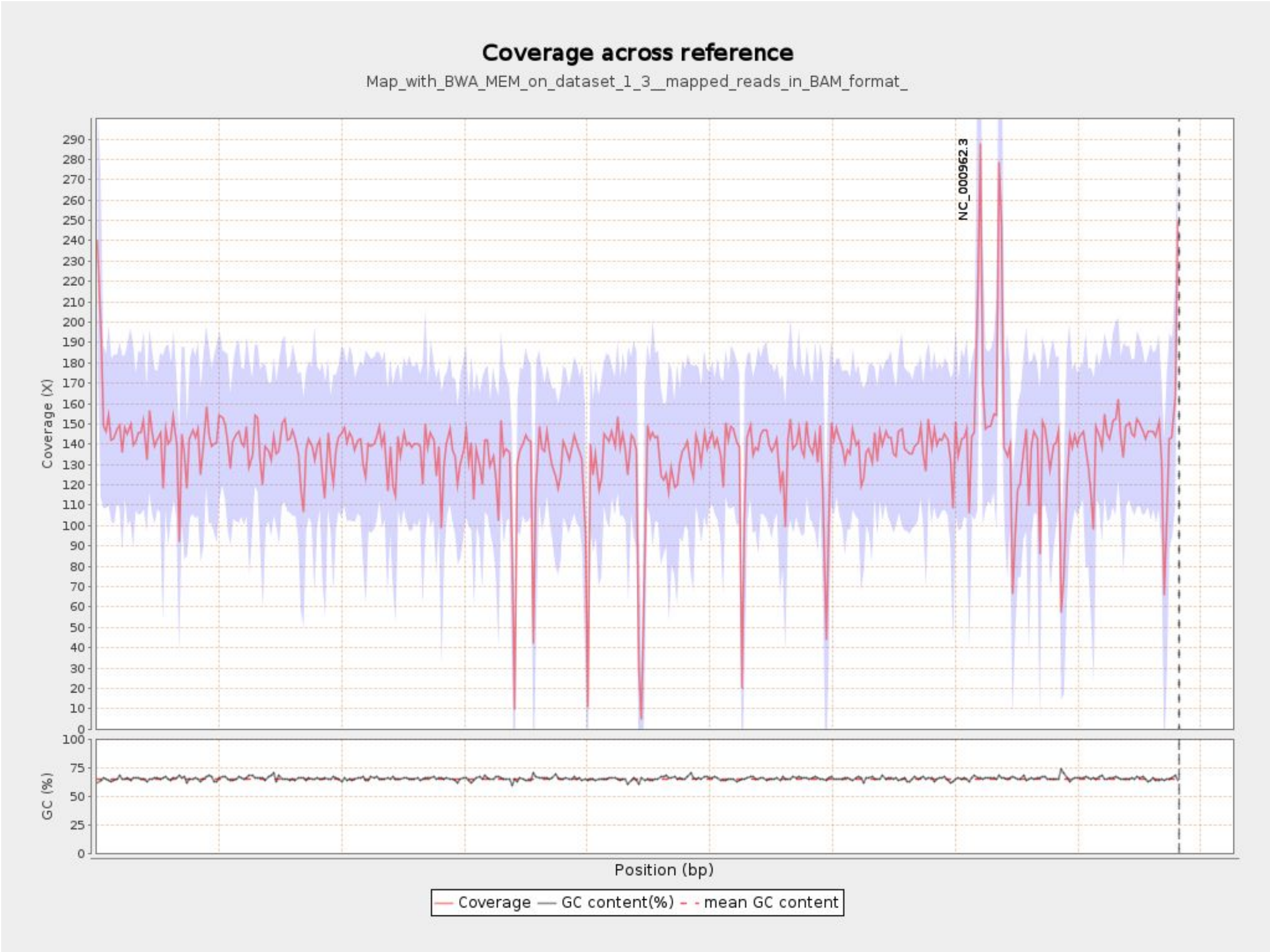

Figure S22: Whole genome coverage for 2nd replicate in the automated test for strain S2

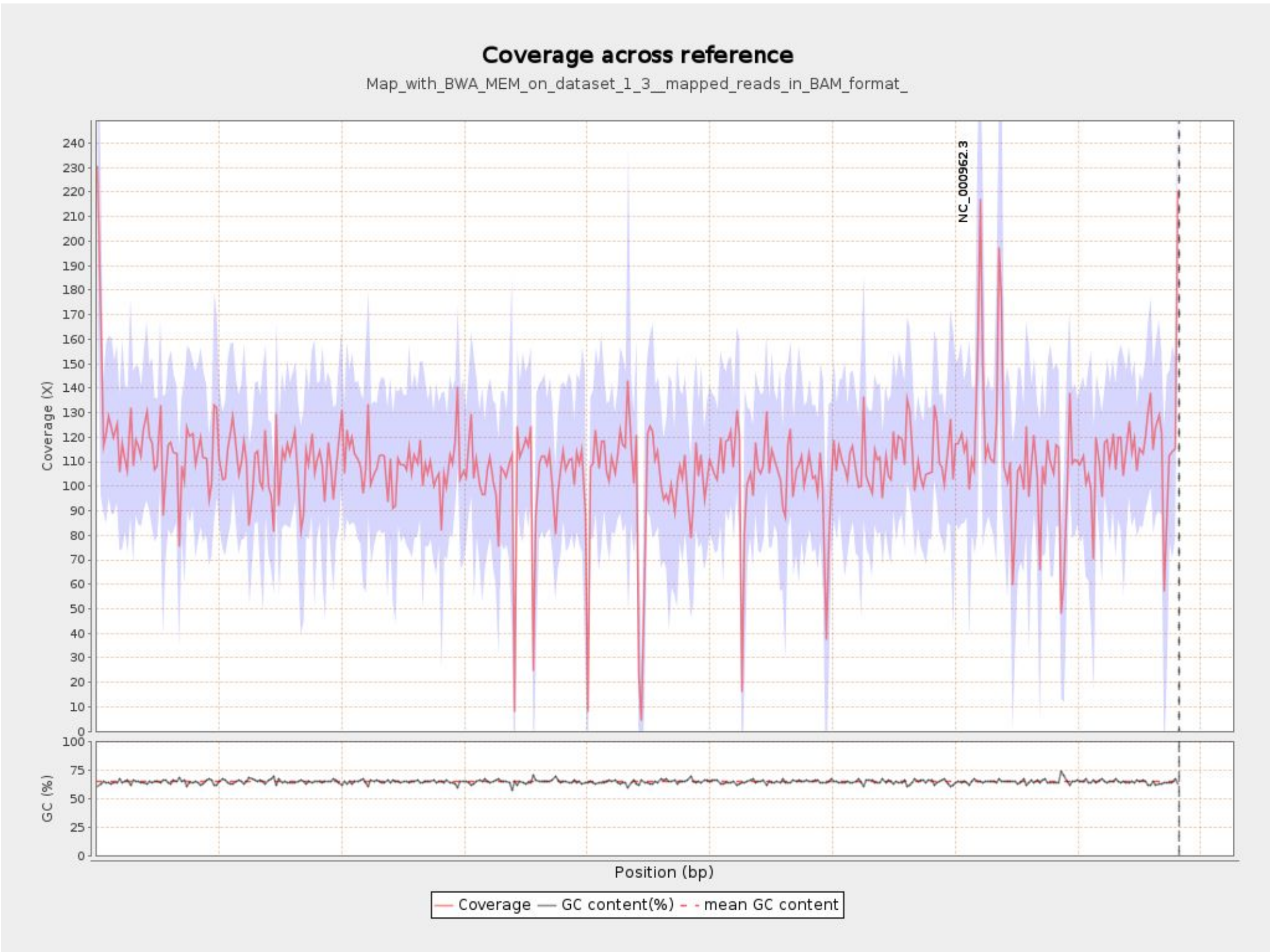

Figure S23: Whole genome coverage for 3rd replicate in the manual test for strain S2

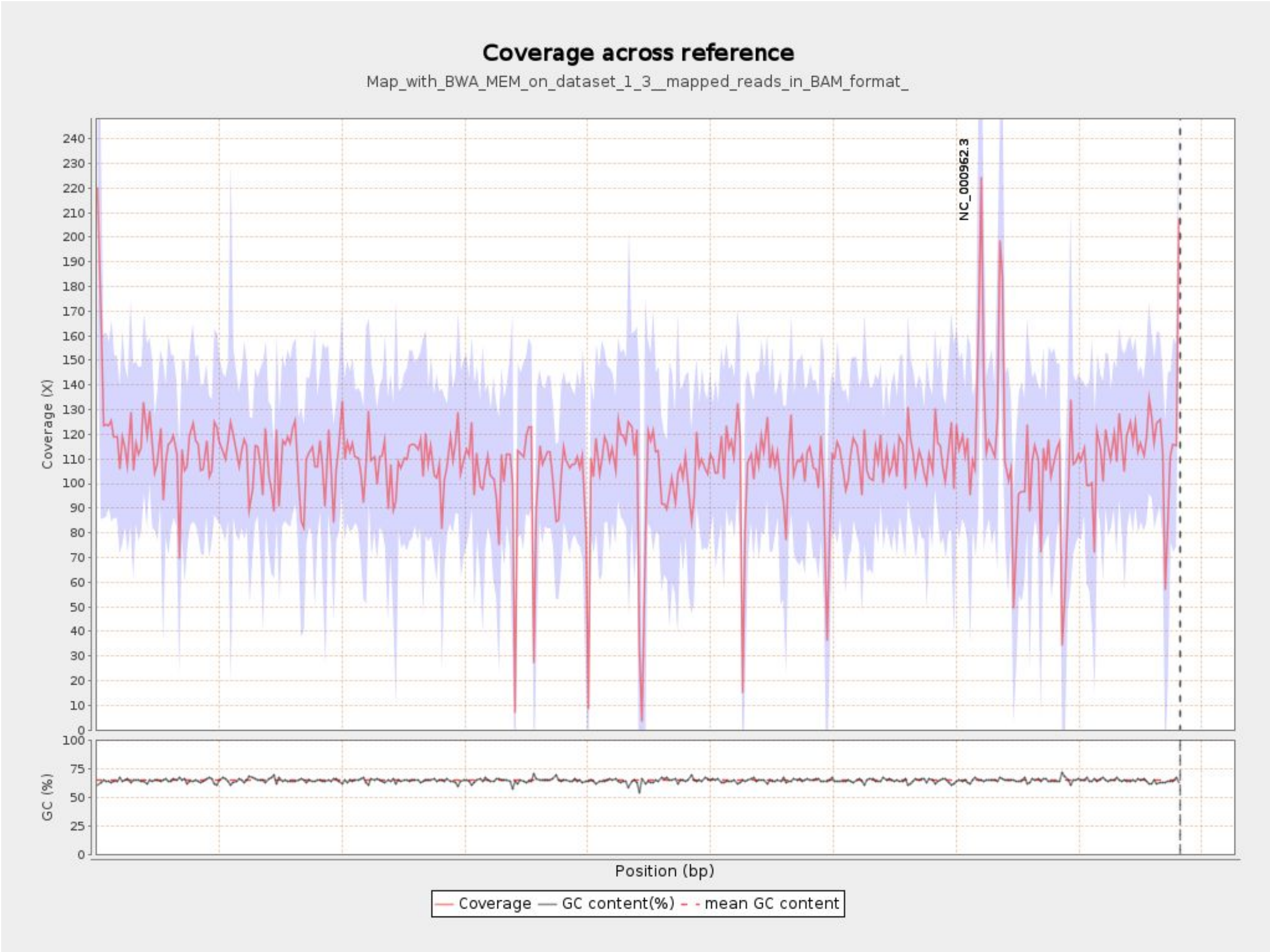

Figure S24: Whole genome coverage for 3rd replicate in the automated test for strain S2

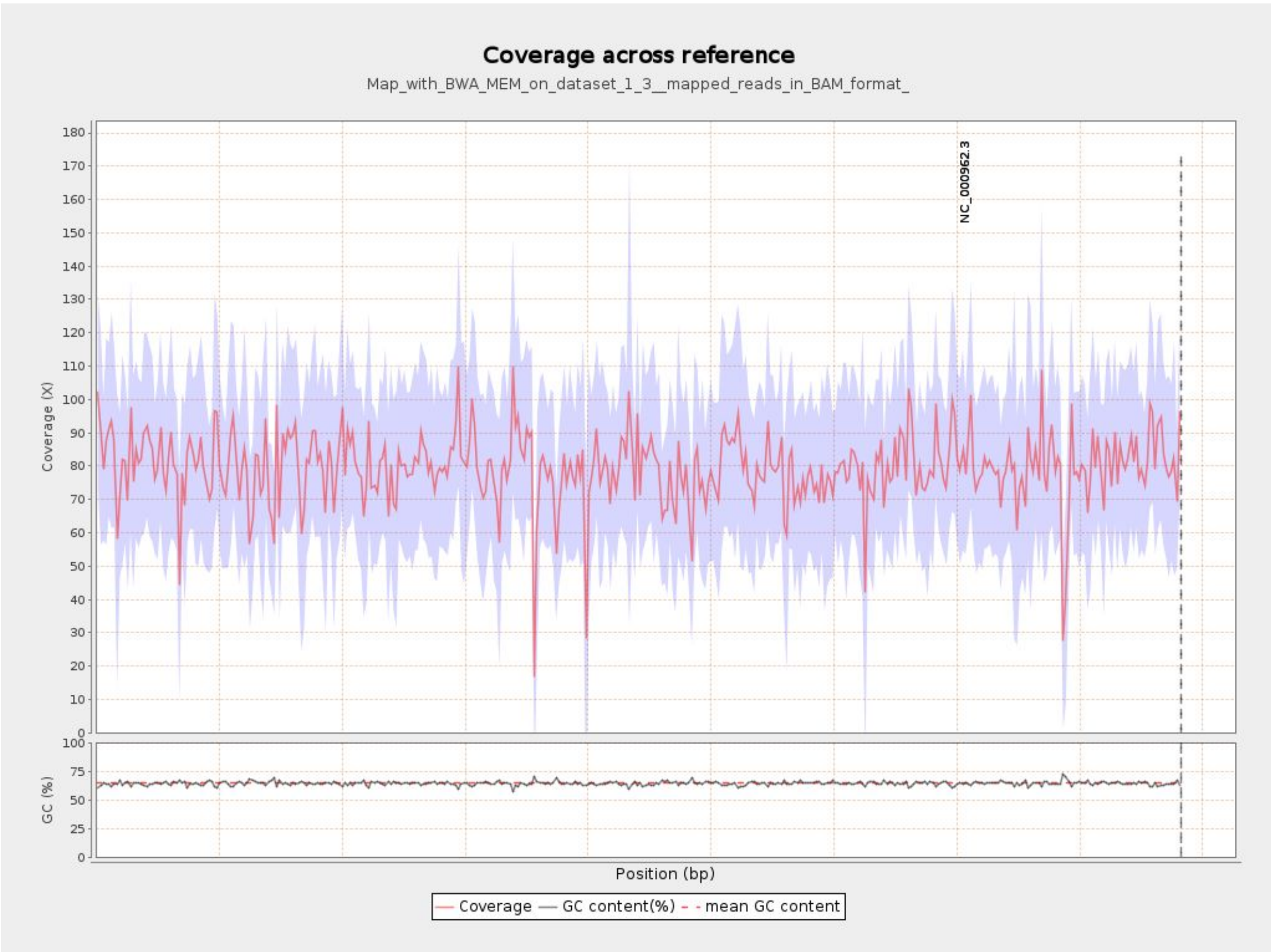

Figure S25: Whole genome coverage for 1st replicate in the manual test for strain S3

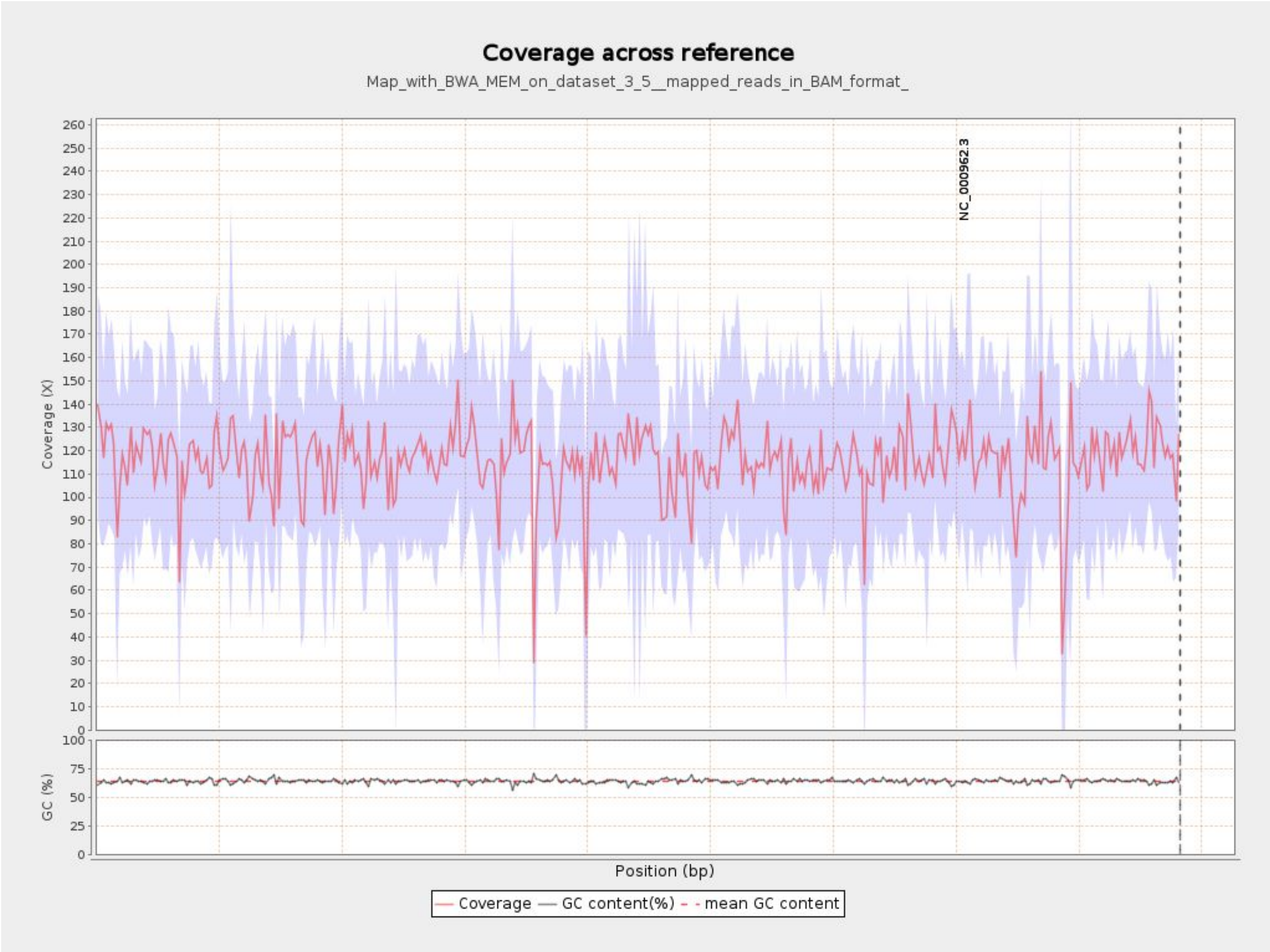

Figure S26: Whole genome coverage for 1st replicate in the automated test for strain S3

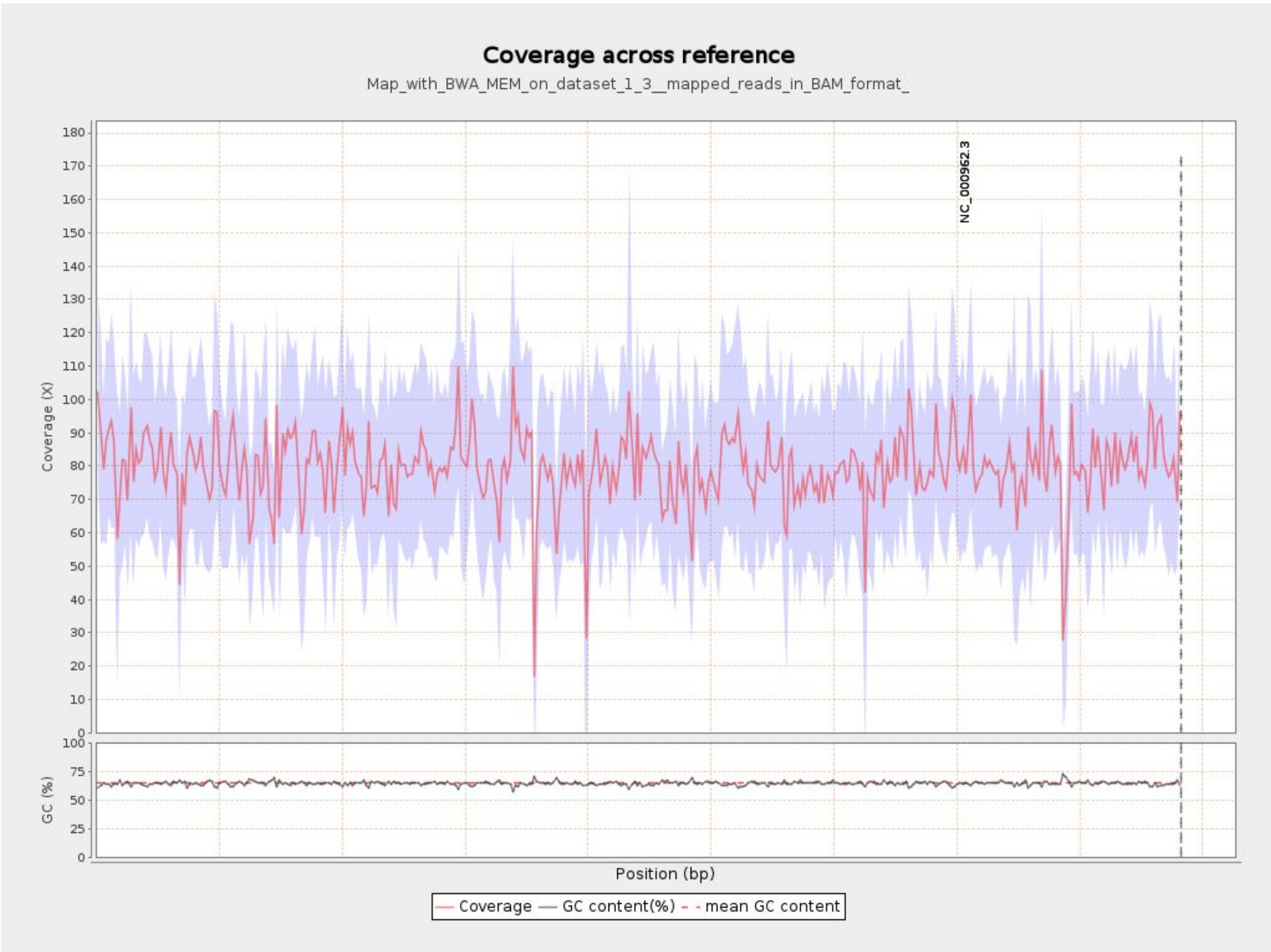

Figure S27: Whole genome coverage for 2nd replicate in the manual test for strain S3

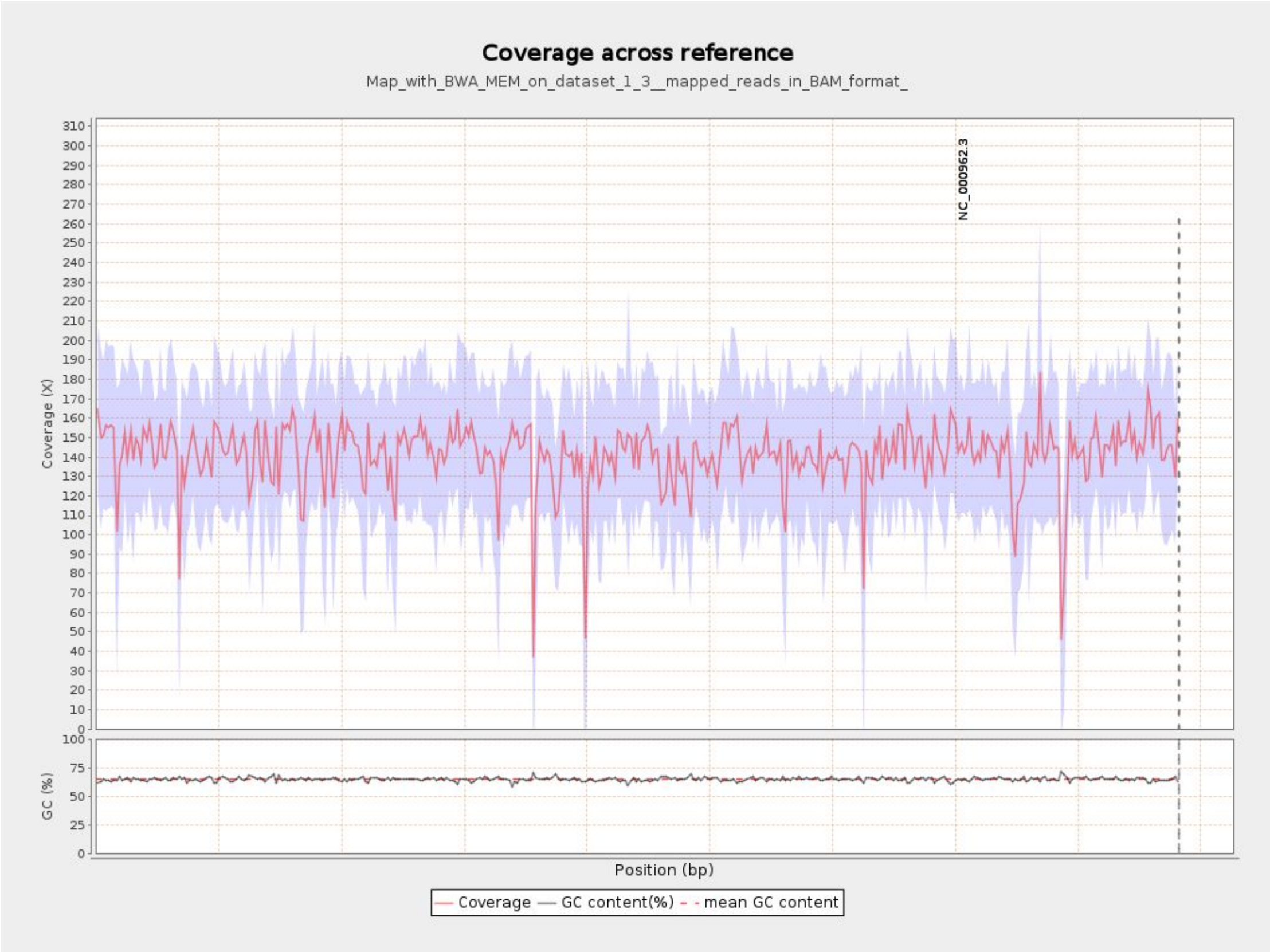

Figure S28: Whole genome coverage for 2nd replicate in the automated test for strain S3

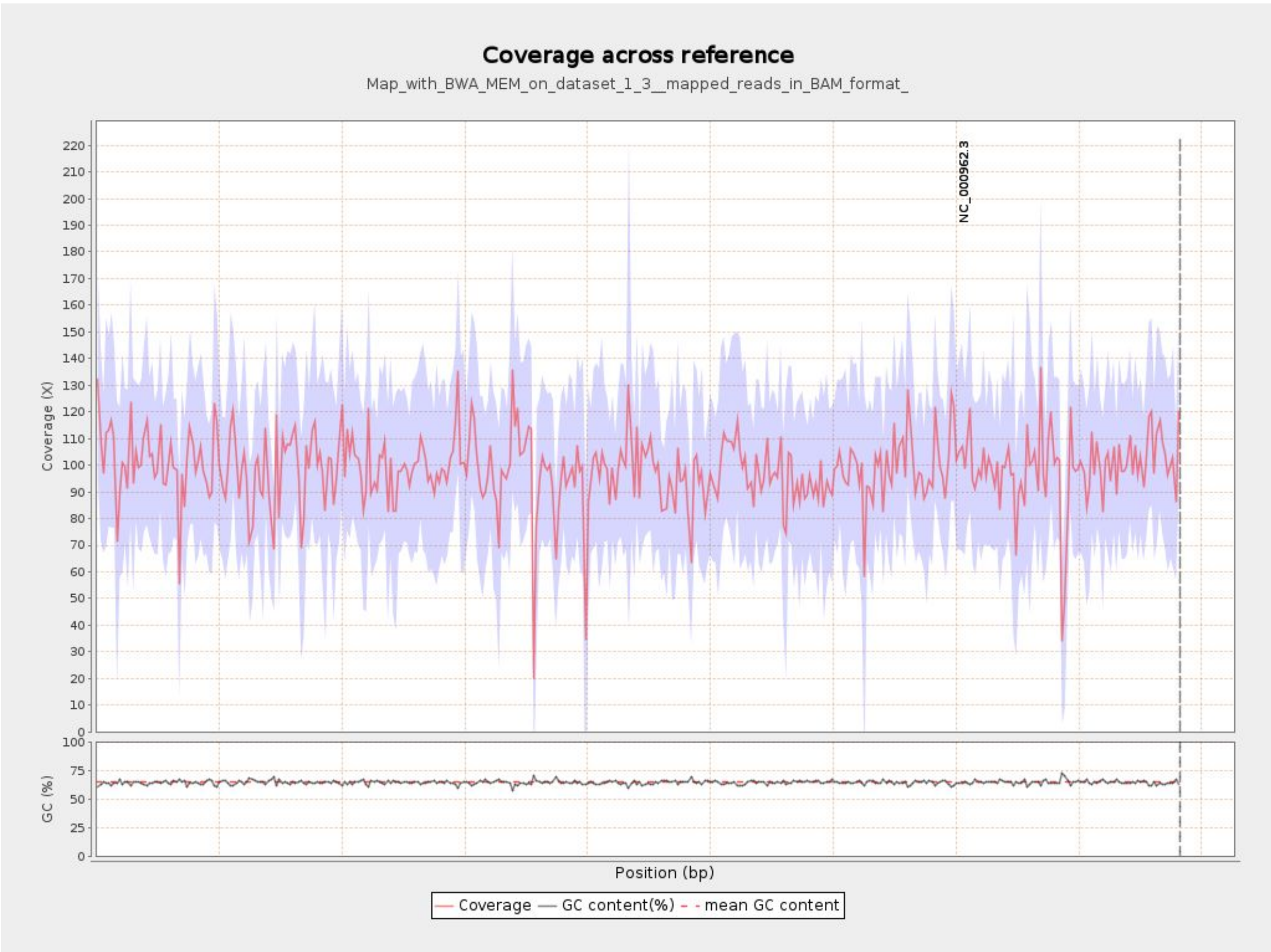

Figure S29: Whole genome coverage for 3rd replicate in the manual test for strain S3

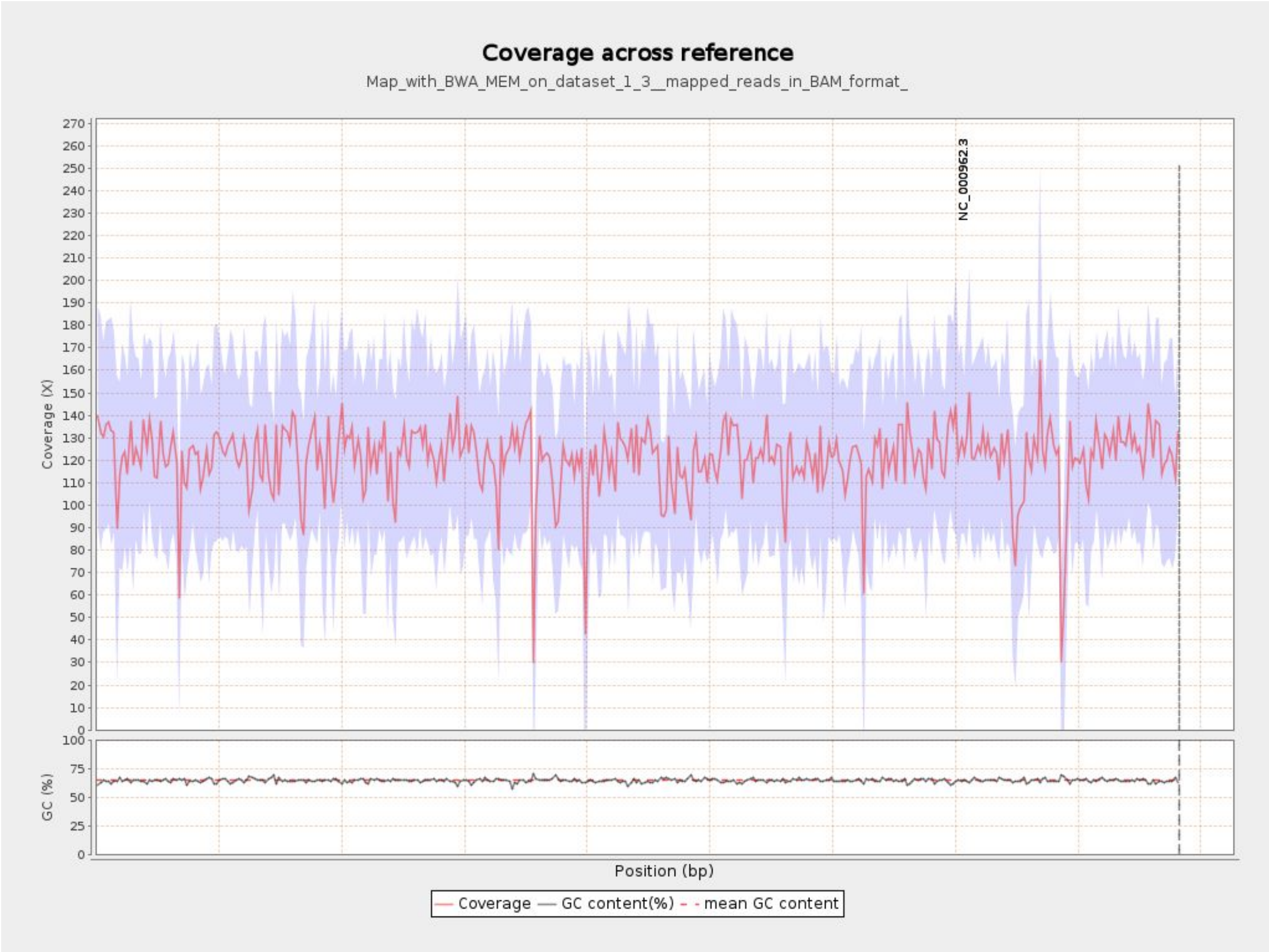

Figure S30: Whole genome coverage for 3rd replicate in the automated test for strain S3

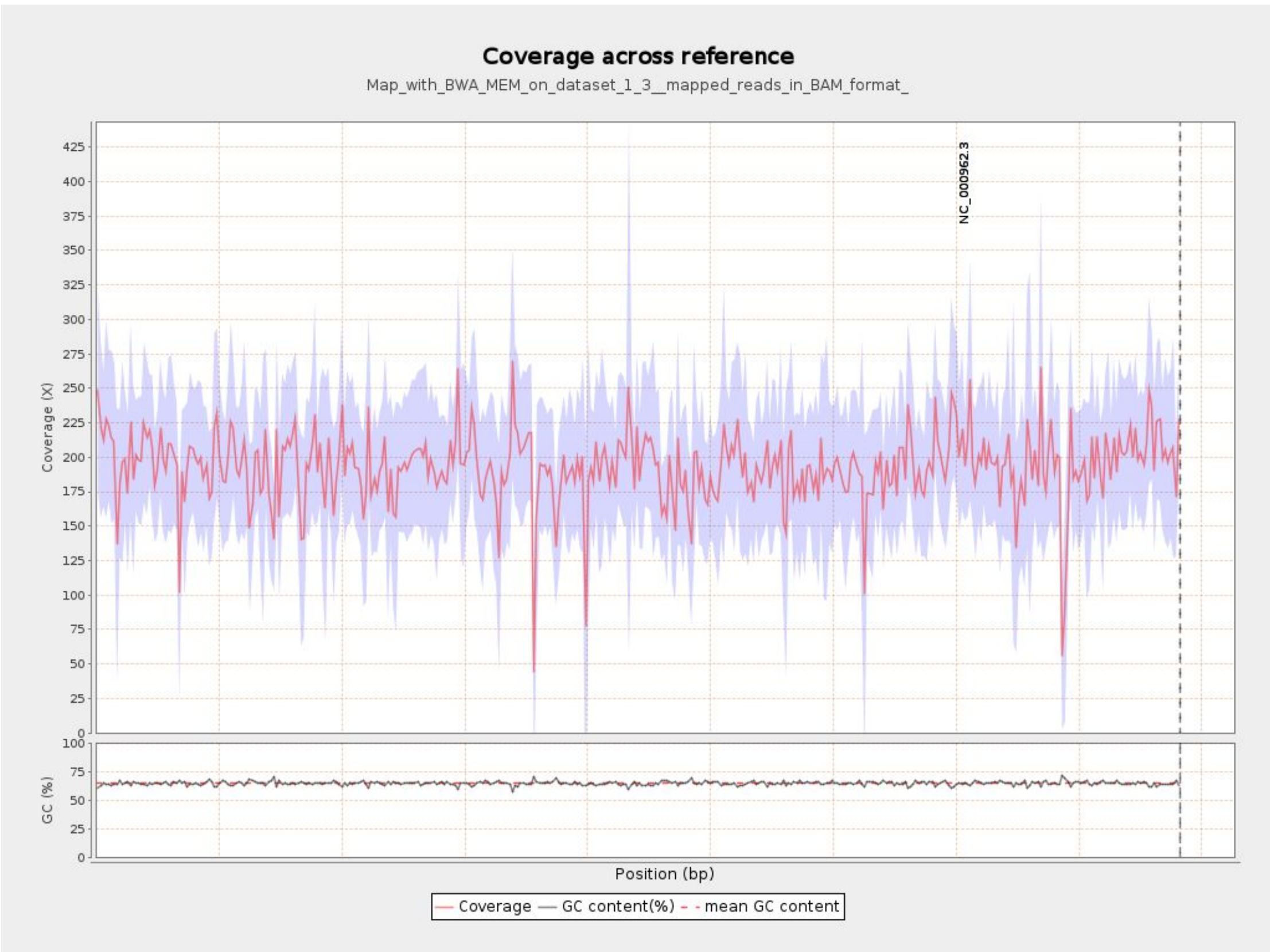

Figure S31: Whole genome coverage for 1st replicate in the manual test for strain S4

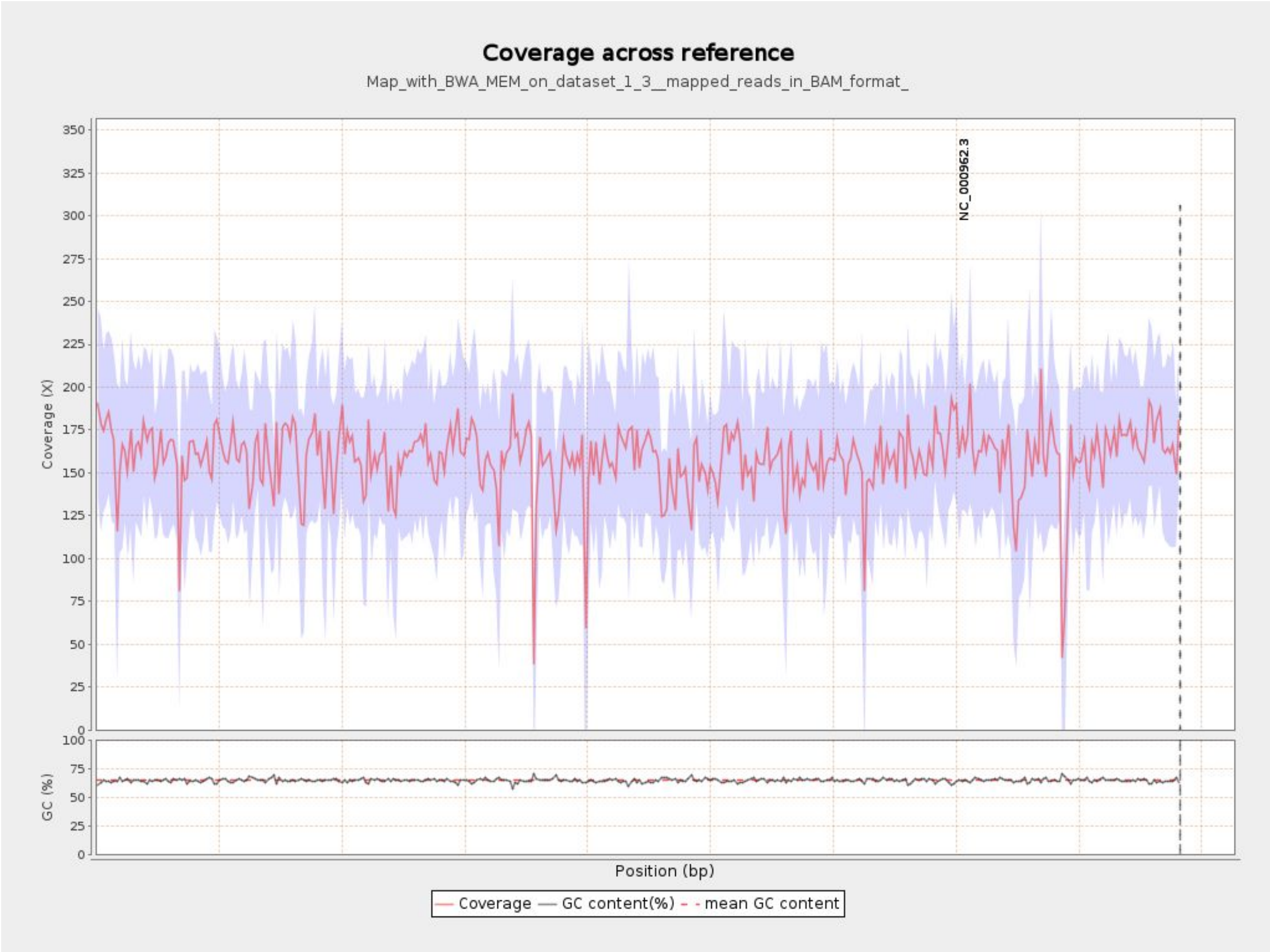

Figure S32: Whole genome coverage for 1st replicate in the automated test for strain S4

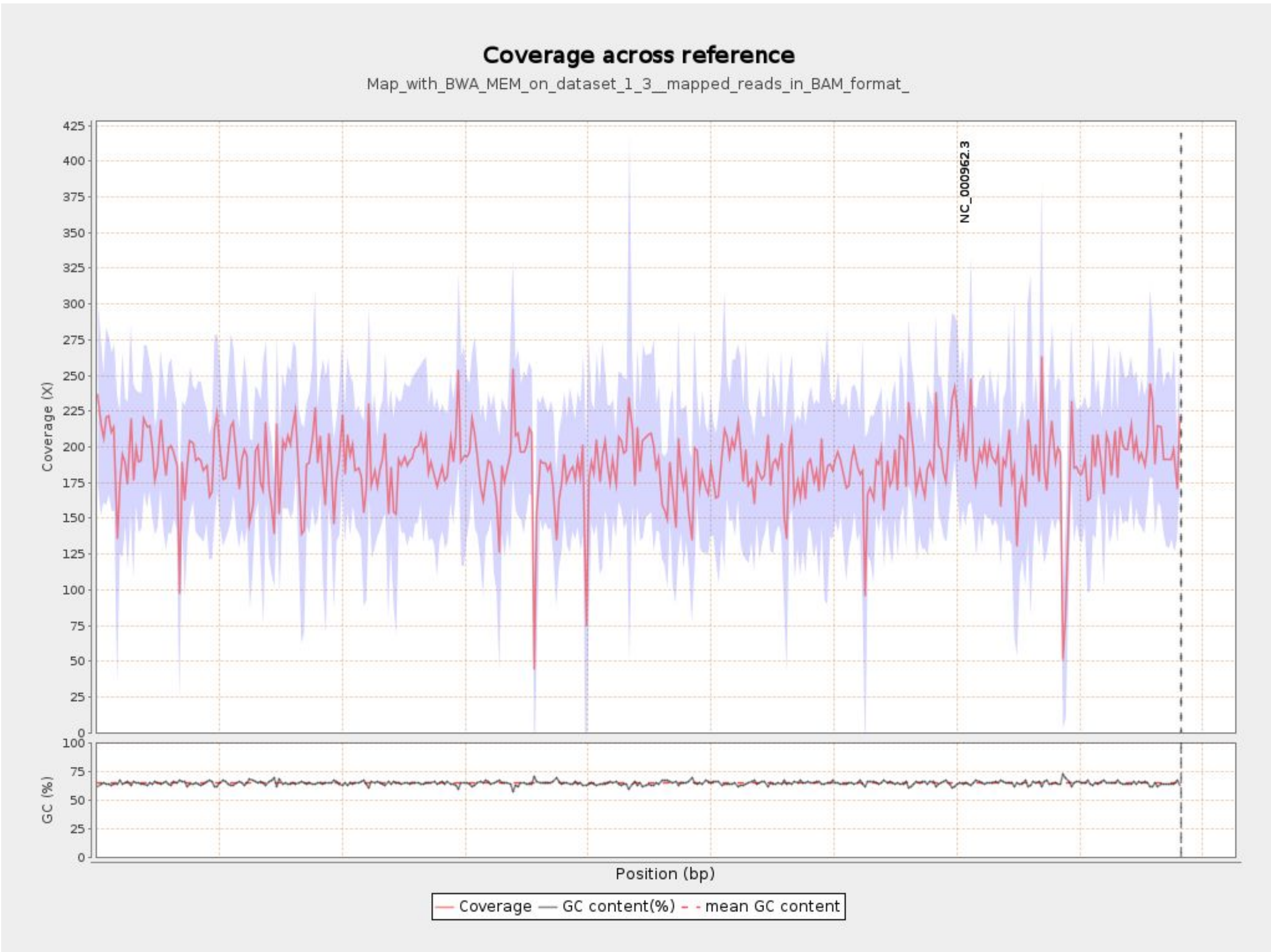

Figure S33: Whole genome coverage for 2nd replicate in the manual test for strain S4

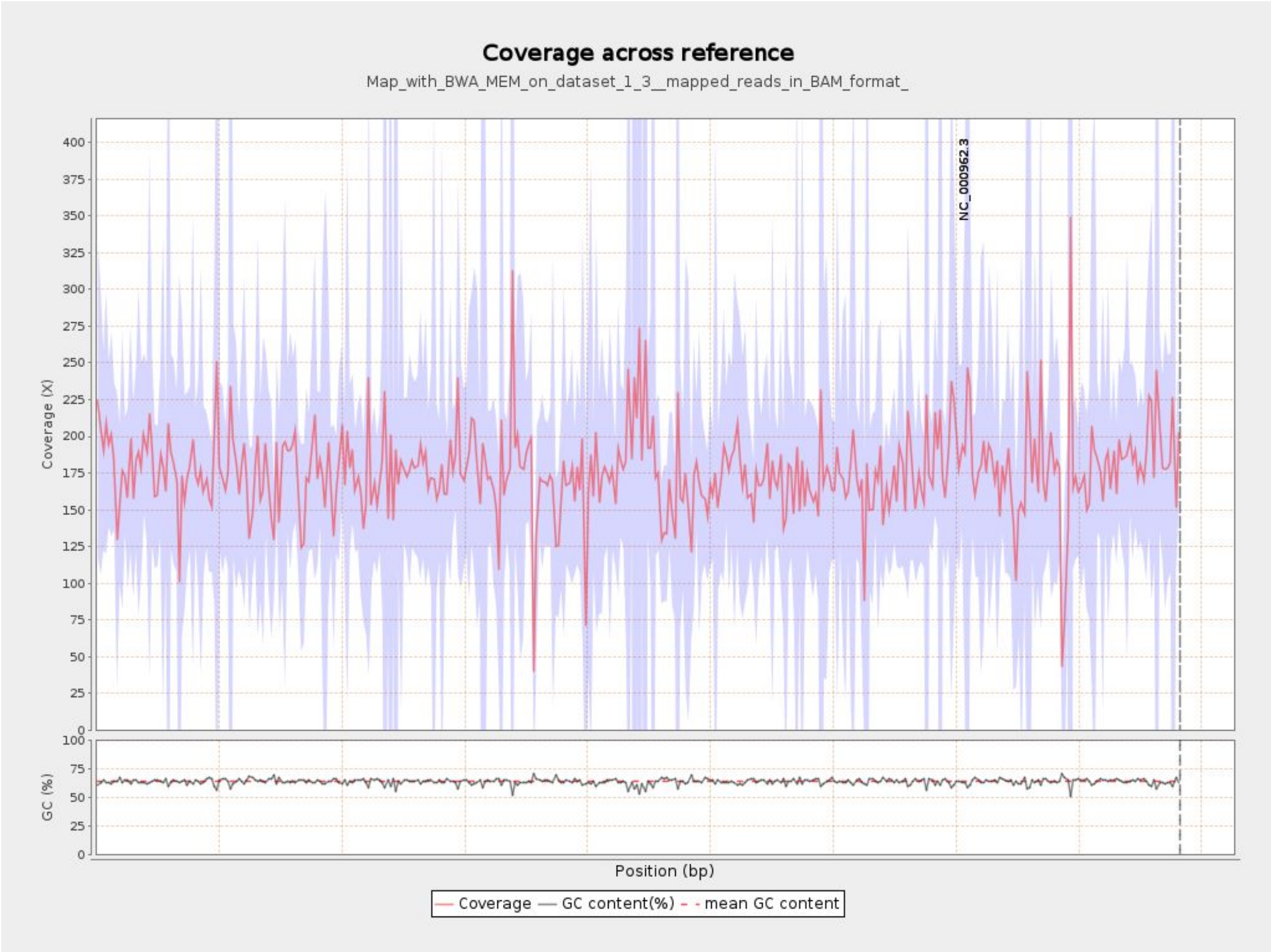

Figure S34: Whole genome coverage for 2nd replicate in the automated test for strain S4

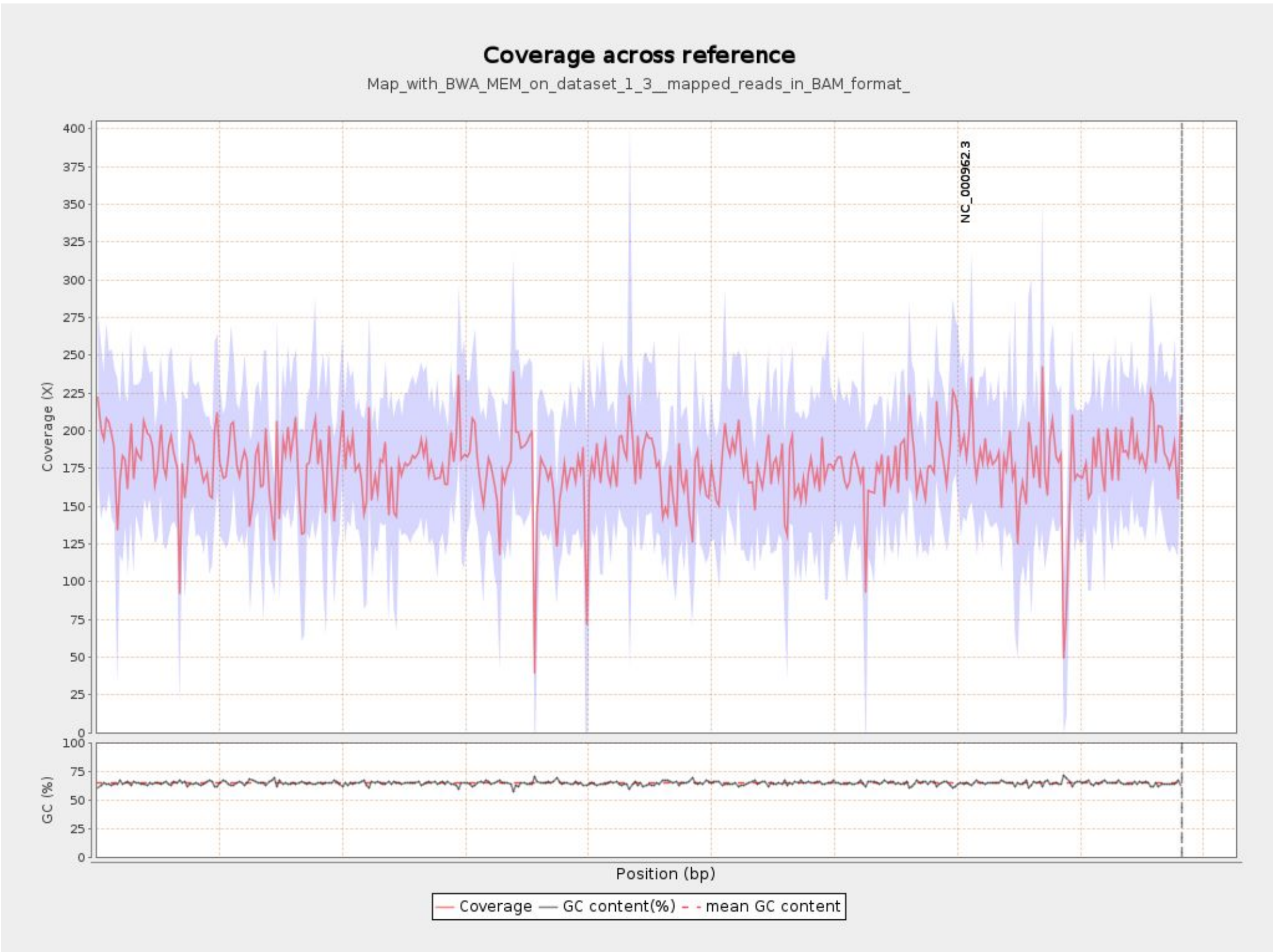

Figure S35: Whole genome coverage for 3rd replicate in the manual test for strain S4

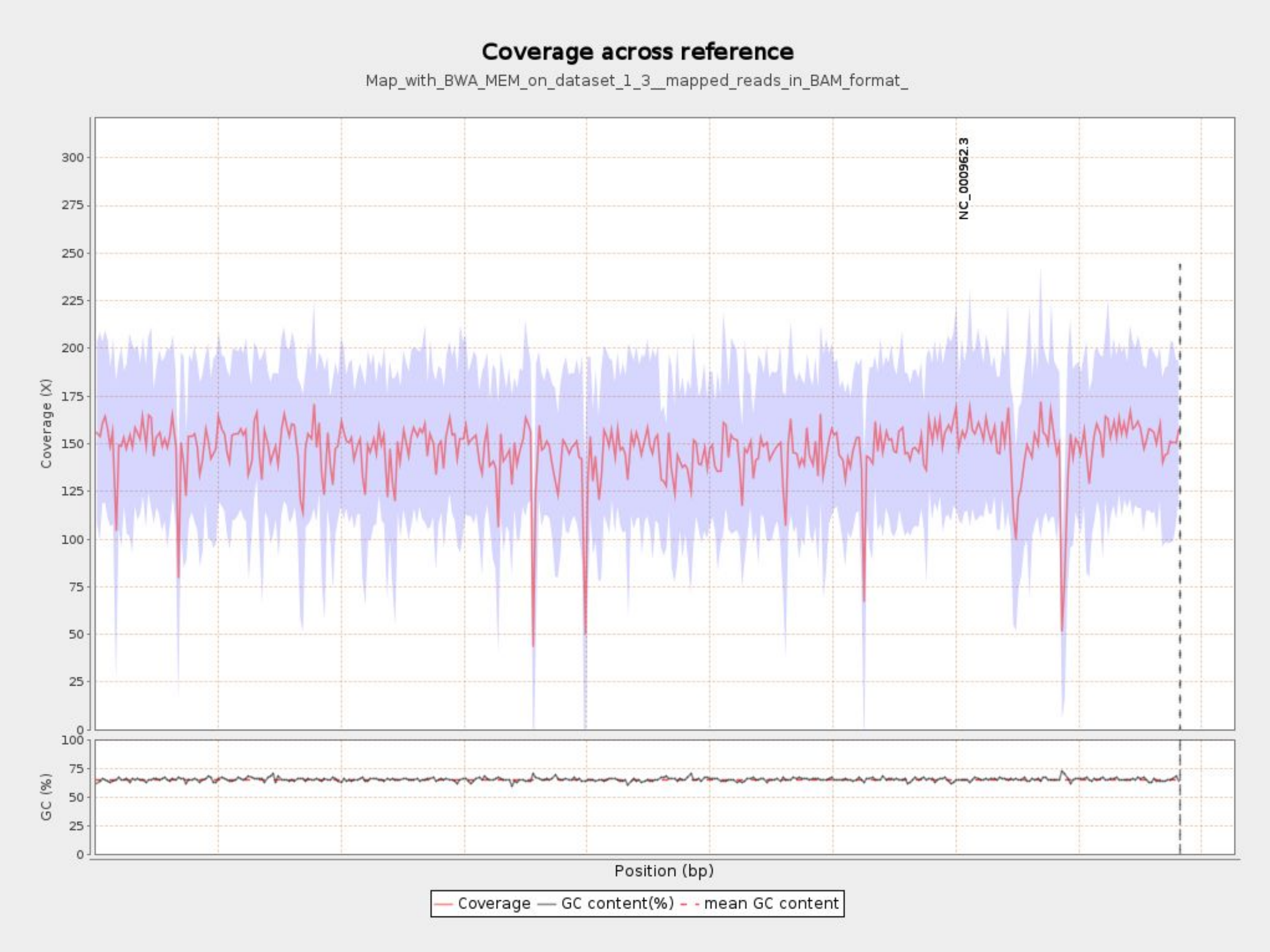

Figure S36: Whole genome coverage for 3rd replicate in the automated test for strain S4

Reference link:

(a) Lee, U. N.; Su, X.; Guckenberger, D. J.; Dostie, A. M.; Zhang, T.; Berthier, E.; Theberge, A. B. *Fundamentals of Rapid Injection Molding for Microfluidic Cell-Based Assays*. Lab Chip **2018**, 18 (3), 496–504. https://doi.org/10.1039/C7LC01052D.

(b) Made-in-China.com. *COC Chemicals Apel APL5014CL Cyclo Olefin Copolymer Resins.* https://sysh888.en.made-in-china.com/product/WajRnQCHgchk/China-COC-Chemicals-Apl5014cl-Cyclo-Olefin-Copolymer-Resins.html (accessed 2026-08-05).

(c) Öffentlicher-Dienst.info. *Entgeltrechner TV-L 2026*. https://oeffentlicher-dienst.info/c/t/rechner/tv-l/allg?id=tv-l-2026&matrix=1 (accessed 2026-08-05).

(d) Meridian Medical. *Medical Device Manufacturing Costs*. https://www.meridian-medical.com/resources/medical-device-manufacturing-costs/ (accessed 2026-08-05).

(e) Mair, D. A.; Geiger, E.; Pisano, A. P.; Fréchet, J. M. J.; Svec, F. Injection Molded Microfluidic Chips Featuring Integrated Interconnects. *Lab Chip* **2006**, 6 (10), 1346–1354. https://doi.org/10.1039/b605911b.

(f) Guan, T.; Yuket, S.; Cong, H.; Carton, D. W.; Zhang, N. Permanent Hydrophobic Surface Treatment Combined with Solvent Vapor-Assisted Thermal Bonding for Mass Production of Cyclic Olefin Copolymer Microfluidic Chips. *ACS Omega* **2022**, 7 (23), 20104–20117. https://doi.org/10.1021/acsomega.2c01948.

(g) MedDeviceGuide. *Cost of Quality in Medical Devices: Prevention, Appraisal, and Failure Costs*. https://meddeviceguide.com/blog/cost-of-quality-medical-devices-guide (accessed 2026-08-05).
(h) Nextera XT DNA Library Prep Kit. Illumina, Inc. https://www.illumina.com/products/by-type/sequencing-kits/library-prep-kits/nextera-xt-dna.html (accessed 2026-08-05).
